# Effectiveness of a Digital Therapy on 6-Month Weight Loss in People With Obesity: The Digital Therapy to Promote Weight Loss in Patients With Obesity by Increasing Their Adherence to Treatment (DEMETRA) Randomized Clinical Trial

**DOI:** 10.2196/72054

**Published:** 2025-10-21

**Authors:** Simona Bertoli, Paolo Capodaglio, Santo Colosimo, Ramona Silvana De Amicis, Luisa Gilardini, Amalia Bruno, Sara Paola Mambrini, Giada Pietrabissa, Luca Cavaggioni, Gianluca Castelnuovo, Giuseppina Piazzolla

**Affiliations:** 1 Obesity Center and Research Lab on Nutrition and Obesity IRCCS Istituto Auxologico Italiano Milan Italy; 2 Department of Food, Environmental and Nutritional Sciences (DeFENS) University of Milan Milan Italy; 3 Musculoskeletal and Metabolic Rehabilitation Unit IRCCS Istituto Auxologico Italiano Milan Italy; 4 Department of Biomedical, Surgical and Dentistry Sciences University of Milan Milan Italy; 5 Clinical Psychology Research Lab, Piancavallo IRCCS Istituto Auxologico Italiano Milan Italy; 6 Department of Psychology Catholic University of Milan Milan Italy; 7 Department of Biotechnology and Life Sciences University of Insubria Varese Italy; 8 University of Bari Aldo Moro Bari Italy

**Keywords:** obesity, digital therapeutics, weight loss, diet, physical activity

## Abstract

**Background:**

Obesity is a chronic, relapsing disease influenced by environmental, lifestyle, biological, and genetic factors, affecting over 1 billion people globally. Treatment for adults typically involves multicomponent lifestyle interventions—diet, physical activity, and behavior change—for at least 6-12 months. However, adherence is often low, and in-person sessions can be time-consuming and costly. Digital therapeutics (DTx), which enhance patient engagement and support long-term outcomes, have proven effective in managing chronic and mental health conditions. DTx offer scalable, evidence-based solutions with the potential to improve obesity management.

**Objective:**

The Digital Therapy to Promote Weight Loss in Patients With Obesity by Increasing Their Adherence to Treatment (DEMETRA) study is a prospective, multicenter, pragmatic, randomized, double-arm, single-blind, placebo-controlled trial evaluating the 6-month efficacy of an innovative, multicomponent digital intervention for obesity, which combines dietary, physical activity, and behavioral strategies in people with obesity (primary objective). Secondary objectives were assessing changes in BMI, waist circumference, blood pressure, glucose metabolism, lipid profile, adherence, and factors associated with absolute 6-month weight loss.

**Methods:**

The trial was conducted at 2 obesity centers in Italy with 246 participants aged 18-65 years (BMI 30-45 kg/m2), randomly assigned to either the Digital Therapeutics for Obesity (DTxO) app or a placebo app. DTxO offered personalized diet plans, exercise routines, and psycho-behavioral support, while the placebo app only allowed users to log data without feedback. Both groups followed a Mediterranean-style low-calorie diet with an 800 kcal/day deficit. On average, participants used the DTxO app for 42 minutes/day and the placebo app for 35 minutes, primarily for physical activity tracking. Univariable and multivariable generalized linear models were used to assess associations with 6-month absolute weight change (primary end point) and percent weight change (secondary end point).

**Results:**

Overall, 207 participants (84.1%) completed the 6-month visit. Both arms achieved a statistically significant absolute (DtxO: –3.2 kg, IQR –6.0 kg to –0.9 kg; placebo: –4.0 kg, IQR –6.9 kg to –0.5 kg; *P*<.001) and percent loss in body weight (DtxO: –3.0%, IQR –5.7% to –0.8%; placebo: –4.0%, IQR –8.5% to –0.5%; *P*<.001) after 6 months, without significant between-group differences (univariable generalized linear models: *P*=.34 and *P*=.17, respectively). Univariable regression analyses showed a significant association between adherence to app use and 6-month absolute weight loss (β=–.06, SE 0.02, *P*=.01) as well as percent weight loss (β=–.05, SE 0.01, *P*=.01). Adherent participants, defined as those with overall adherence at or above the 75th percentile of daily usage, included 35 individuals in the intervention group and 10 in the placebo group. In this subgroup, the estimated 6-month mean absolute weight change was –7.02 kg (95% CI –9.45 to –4.59) in the DTxO-adherent group and –3.50 kg (95% CI –7.01 to 0.01) in the placebo-adherent group (*P*=.02). The estimated 6-month mean percent change in weight was –6.31% (95% CI –8.86 to –3.76) in the DTxO-adherent group and –2.78% (95% CI –6.48 to 0.92) in the placebo-adherent group (*P*=.03). A significantly greater weight loss (*P*=.01 for study arm, either on absolute or percent change in weight from baseline) among adherent participants randomized to the DTxO app was also confirmed by analyses using mixed linear models for repeated measures.

**Conclusions:**

Although overall weight loss did not differ significantly between the DTxO and placebo groups, participants who used the DTxO app for at least 40% of the expected time achieved significantly greater weight loss. These results suggest that higher engagement with DTx can improve obesity outcomes. Further research should explore combining DTxO with pharmacological treatments or bariatric surgery.

**Trial Registration:**

ClinicalTrials.gov NCT05394779; https://clinicaltrials.gov/ct2/show/NCT05394779

**International Registered Report Identifier (IRRID):**

RR2-10.3389/fdgth.2023.1159744

## Introduction

Obesity is a chronic, relapsing disease resulting from the complex interaction of environmental factors, lifestyle choices, and genetically determined metabolic alterations [1]. It is associated with a range of comorbidities that negatively affect life expectancy, including hypertension, dyslipidemia, type 2 diabetes, obstructive sleep apnea, and elevated risks of cardiovascular disease, cancer, and infections [2]. Furthermore, obesity has profound consequences for patients’ quality of life, leading to social and psychological impairments as well as functional limitations [3]. It is considered a global pandemic, currently affecting approximately 1 billion people worldwide, including both adults and children [4].

The treatment of people living with obesity continues to present significant challenges for health care professionals. For adults, guidelines recommend managing obesity as a chronic disease through multidisciplinary teams. These guidelines advocate multicomponent lifestyle interventions—comprising diet, physical activity, and behavior change strategies—for at least 6 to 12 months [5-7]. Dietary recommendations emphasize personalized, calorie-reduced plans combined with nutrition education to support sustainable weight loss and overall health. Physical activity should be tailored to individual abilities and health status, with at least 150 minutes of moderate aerobic exercise plus twice-weekly strength training recommended [8]. Behavioral strategies encourage mindfulness-based interventions to address emotional eating and stress, thereby enhancing coping skills, self-regulation, and psychological well-being [8,9]. Mindful eating practices increase awareness of eating behaviors and support long-term weight maintenance [10]. Together, these strategies provide a holistic, personalized approach to obesity management, emphasizing ongoing support. Notably, even when combined with pharmacotherapy—such as novel gut hormone receptor agonists—or bariatric surgery, lifestyle interventions remain essential for achieving effective and sustained outcomes [11]. However, despite these efforts, a persistent challenge in obesity management—both short and long term—is patient adherence to lifestyle changes. Achieving desired outcomes often requires multiple in-person sessions, which can be time-consuming, costly, and demanding for both patients and health care services [12].

In this context, alternative health care delivery models may be crucial for more effective obesity management, and digital therapeutics (DTx) could play a pivotal role in improving and scaling interventions. DTx refers to “evidence-based therapeutic interventions delivered through high-quality software programs to prevent, manage, or treat medical disorders or diseases,” and must be certified by regulatory bodies as medical devices [13].

A key aspect of DTx is the inclusion of patient engagement modules, comparable to excipients in a drug, designed to enhance patients’ interaction with the software.

While DTx show promise for obesity treatment, only a few randomized controlled trials (RCTs) have evaluated their efficacy, often focusing on isolated behavioral strategies such as self-monitoring or time-restricted eating [14-18]. Most rely on smartphones, with some incorporating web platforms or wearables, but only a few employ comprehensive, multidimensional outcome measures [17]. This variability limits comparability and highlights the need for more robust evaluations.

A recent meta-analysis of smartphone app–based interventions for weight loss reported a modest average reduction of 2.03 kg after 3 months of app use. However, the significant heterogeneity across studies suggests that while these apps can be effective, their success is influenced by various factors, such as the integration of a multidimensional approach [18].

The aim of the Digital Therapy to Promote Weight Loss in Patients With Obesity by Increasing Their Adherence to Treatment (DEMETRA) study is to evaluate the performance (6-month weight loss) and safety of a novel DTx (Digital Therapeutics for Obesity [DTxO], intervention arm) encompassing a comprehensive set of interventions, including a personalized dietary plan, a tailored exercise program, a mindfulness component specifically targeting behaviors related to dietary intake and mindful eating, and reminders for medication adherence. Here, we present the findings at 6 months of follow-up.

## Methods

### Study Design

The DEMETRA study is a prospective, multicenter, pragmatic, randomized, double-arm, single-blind, placebo-controlled trial. The methods have been detailed in a previously published protocol paper [19], and no changes were made to the protocol during the study. The trial was prospectively registered on ClinicalTrials.gov (identifier NCT05394779) on August 23, 2022.

The primary objective of the study was to evaluate weight loss in patients using DTxO compared with control patients after 6 months of use. Secondary objectives included the 6-month assessment of changes in clinical and metabolic parameters (waist circumference, blood pressure, fasting glucose, insulin resistance, and lipid profile), study adherence, and the evaluation of factors associated with 6-month weight loss.

Eligible patients were adults aged 18-65 years with a BMI between 30 and 45 kg/m^2^, and proficient in using mobile apps, as the digital therapy was app based. As all instructions and guidance were provided in Italian, participants were required to be fluent in the language.

Patients were excluded from the study if they had cardiovascular events, severe heart failure, ischemic attack, or stroke within 6 months of screening; chronic kidney failure; type 1 diabetes; previous malignancy within the past 5 years; visual impairments (eg, complete or nearly complete vision loss, glaucoma); secondary obesity related to endocrinopathies, genetic syndromes, or hypothalamic lesions; advanced obesity disease (stage 4 on the Edmonton Obesity Staging System) [20]; uncontrolled psychiatric disorders; active eating disorders or a history of bulimia or anorexia nervosa; active substance abuse; history of bariatric surgery within the past 2 years or plans for surgery (eg, sleeve gastrectomy, gastric banding, gastric bypass); changes in pharmacological treatments affecting appetite or metabolism within 3-6 months of screening; participation in other weight-loss programs or trials; referred pain in lower limb joints (hip, knee, ankle) with a Numeric Rating Scale score ≥5 [21]; Binge Eating Scale score >27 [22]; uncompensated psychiatric disorders, defined as a score ≥2 in the depression, anxiety, or psychoticism domains [23]; or weight loss 10% or over in the 6 months before randomization.

The enrollment process involved adults with obesity who independently sought weight-loss treatment at participating centers. During their initial interaction with clinical staff, they were informed about the research opportunity. Those who expressed interest were invited to a baseline visit, where eligibility was assessed according to predefined inclusion and exclusion criteria. Written informed consent was obtained before any study-related procedures.

The recruitment phase lasted 4 months, followed by a 6-month initial follow-up period. To ensure consistency in assessment and care, all baseline and follow-up visits were conducted by the same clinical team at each participating center.

### Setting

The trial was conducted at 2 obesity care centers in Italy: IRCCS Istituto Auxologico Italiano (Center 1) in Northern Italy and Policlinico di Bari, Giovanni XXIII Hospital (Center 2) in Southern Italy. Center 1 is a Scientific Institute for Hospitalization and Care (IRCCS), operating in collaboration with the National Health System. Its Obesity Unit is a tertiary care facility and a designated Collaborating Centre for Obesity Management by the European Association for the Study of Obesity. A multidisciplinary team of physicians, nurses, dietitians, physical activity trainers, and clinical psychologists is dedicated to both clinical care and research. Patient care is tailored to the severity of obesity and related complications and includes outpatient consultations, outpatient rehabilitation programs, and inpatient functional metabolic rehabilitation. The center treats more than 10,000 patients with obesity annually, 40% of whom are classified as severely obese (BMI >40 kg/m^2^).

Center 2 is an Internal Medicine Unit within the University Hospital Consortium—Bari Aldo Moro. The unit is part of a large academic medical facility with extensive experience in managing metabolic disorders, including obesity, metabolic syndrome, type 2 diabetes, metabolic dysfunction–associated fatty liver disease, and dyslipidemia. The outpatient clinic is coordinated by a multidisciplinary team, including internal medicine specialists, geriatricians, dietitians, clinical psychologists, sonographers, and both junior and senior researchers. Patients are referred by primary care physicians, specialists from other departments, or may self-refer for metabolic evaluation and treatment. Each year, the center sees approximately 5000 patients with metabolic disorders, most seeking support for weight management or obesity-related conditions.

### Timeline

Study procedures included a baseline visit and a face-to-face follow-up visit 6 months after enrollment. During the baseline visit, participants’ medical and family history were collected, along with information on current and past pharmacological treatments, menopausal status, lifestyle habits such as smoking and structured physical activity, and sociodemographic data. Each participant underwent a physical examination, and blood pressure was measured in accordance with international guidelines from the European Society of Hypertension and the European Society of Cardiology [24]. A fasting blood sample was collected between 8:30 and 9:00 AM to measure blood glucose, insulin, triglycerides, total cholesterol, low-density lipoprotein cholesterol, high-density lipoprotein cholesterol, alanine transaminase, aspartate transaminase, gamma-glutamyl transferase, thyroid-stimulating hormone, and free thyroxine. The insulin resistance index and glomerular filtration rate were calculated using validated formulas [25,26].

Anthropometric measurements were assessed by a registered dietitian in accordance with international guidelines [27]. Weight was measured using an electronic scale with 100 g accuracy (Seca 700; Seca Corporation), and height was measured using a vertical stadiometer with 0.1 cm accuracy. Waist circumference was measured to the nearest 0.5 cm using a nonelastic tape placed at the midpoint between the last rib and the iliac crest.

Finally, patients completed the International Physical Activity Questionnaire (IPAQ) to assess physical activity levels [28], and adherence to the Mediterranean dietary pattern was evaluated using a validated 14-item questionnaire [29].

During the follow-up visit, the same parameters collected at baseline were reassessed. Additionally, concomitant medications were recorded, and a safety assessment was performed.

### Randomization

Patients were randomly assigned to the DTxO app or the placebo app (control group) on a 1:1 basis using a predefined, centralized randomization list. To maintain overall balance between groups, block randomization was performed with random block sizes of 8 patients, using the “proc plan” procedure in SAS (version 9.4; SAS Institute).

Blinding of physicians, dietitians, and psychologists was not possible due to the nature of the intervention.

### Evidence-Based Lifestyle Theory for Intervention Strategies

The intervention in this study was designed based on evidence-based strategies recommended by international clinical practice guidelines for the management of overweight and obesity [6-8]. These guidelines advocate a comprehensive, multicomponent lifestyle approach that integrates caloric restriction, aerobic exercise, and behavior change techniques. Central to the behavioral component is the incorporation of mindfulness—a practice defined as the cultivated ability to maintain present-moment awareness with an open, nonjudgmental attitude [9]. Extensive research shows that mindfulness enhances individuals’ awareness of habitual thoughts, emotions, and behaviors, promoting more adaptive and conscious responses to internal and external cues. Clinically, this heightened awareness supports improved emotional regulation, greater self-compassion, and stronger self-control, which are key mechanisms for addressing dysfunctional eating behaviors and fostering long-term adherence to lifestyle changes [9].

We hypothesize that DTxO, by integrating evidence-based components into a single, user-friendly digital platform, can provide continuous, personalized guidance and reinforcement, potentially facilitating weight loss in individuals living with obesity.

The entire therapeutic algorithm was developed in accordance with clinical guidelines promoting a multidisciplinary approach, involving health care professionals such as physicians specializing in nutrition (clinical nutritionists or endocrinologists), dietitians, kinesiologists, and psychologists [6-8].

DTxO was developed by Advice Pharma Group S.r.l. and is classified as a Class IIa medical device software under the European Medical Device Regulation (EU MDR 2017/745, Rule 11 of Annex VIII). The placebo app was developed by the investigative team to simulate the interface and user experience of DTxO, without delivering personalized or adaptive digital therapeutic content.

### Components Received by Both Study Groups

#### Nutritional Prescription

Both groups followed the same energy-restricted diet and regularly self-monitored dietary adherence, physical activity, and weight trends. Specifically, the dietary intervention, based on the Italian Guidelines for Dietary Obesity Management [30], provided a personalized Mediterranean-style low-calorie diet. Daily caloric intake was calculated using a standardized method, applying an 800-kcal deficit relative to estimated total energy expenditure, which was determined using the Mifflin-St Jeor formula [31] for resting energy expenditure. This estimate was further adjusted according to physical activity levels, assessed using the short version of the IPAQ [28].

The diet was designed to provide 45%-50% of total calories from carbohydrates and 30%-35% from fats, sourced from typical Mediterranean foods such as nuts, extra virgin olive oil, fish, and whole grains.

#### Self-Monitoring Procedures

Self-monitoring of adherence to the dietary plan, physical activity, and weight trends required participants to enter data into the platform weekly for diet and physical activity, and biweekly for weight. Table S1 in Multimedia Appendix 1 summarizes the components provided to both study groups.

### Intervention Arm (DTxO)

**Figure 1:**
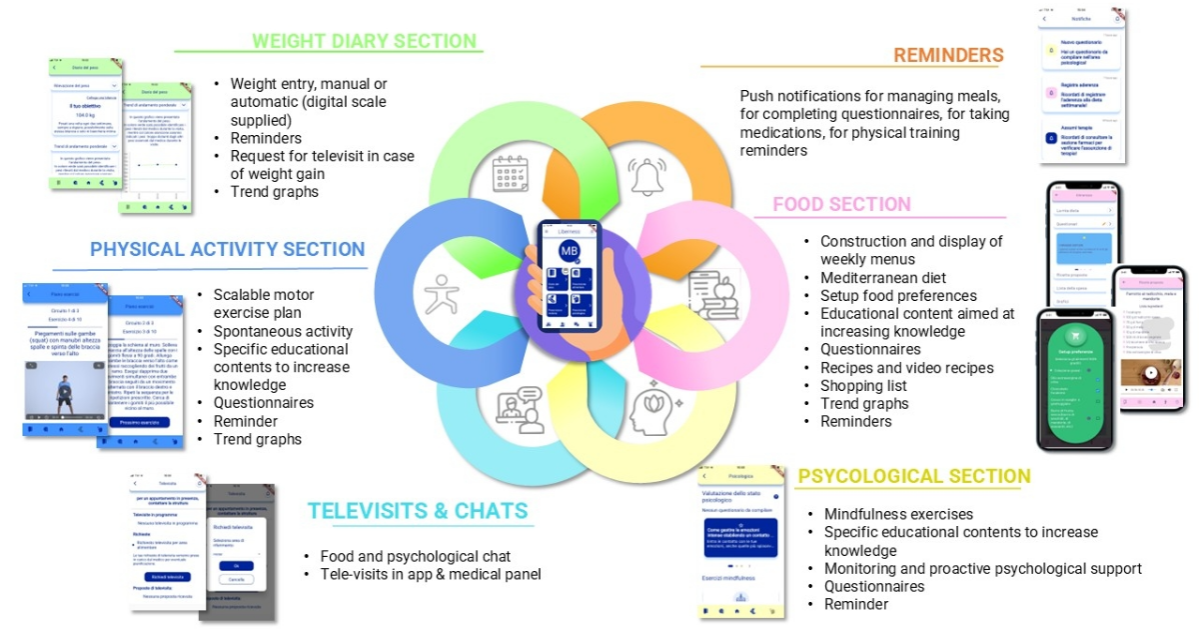
Multidimensional approach: DTxO app offers a personalized diet and exercise plan, cognitive behavioral support, reminders, drug intake tracking, and online communication with health care professionals. DTxO: Digital Therapeutics for Obesity.

Patients in the intervention arm had access to the following interactive and multimedia sections (Figure 1).

### Main DTxO Sections and Features

#### Dietary Section

Patients could personalize their assigned dietary program by selecting the number of meals and food preferences from a list of options for each food category. They could either create their own personalized menu or follow the general diet structure while adhering to prescribed portions and food frequencies. The DTxO app also allowed users to organize meals through a shopping list and provided access to supporting recipes. Additionally, patients received educational modules on food, hydration strategies, and uncommon foods (eg, quinoa or legume derivatives) to encourage variety in their diet.

#### Physical Activity Section

The physical activity intervention was tailored to each individual’s fitness level, based on the baseline IPAQ [28]. Patients received a personalized exercise program, including recommendations on type, duration, frequency, and intensity of activity. After each session, patients rated their perceived fatigue and any pain using a numerical rating scale [12]: 0=no effort/pain (too soft), 3=moderate effort/pain (tolerable), and 10=maximum effort/pain (too hard). Based on this feedback, the DTxO app automatically adjusted the type, duration, frequency, and intensity of subsequent exercises. Physical activity was self-reported, and exercise sequences were accompanied by short explanatory videos and educational content tailored to each activity.

#### Psycho-Behavioral Section

The psycho-behavioral section included educational videos, audio-guided exercises, self-assessments, and dynamic exercises across 5 key areas to support control and containment efforts. These areas were (1) commitment and motivation, (2) openness and availability, (3) awareness and mindfulness, (4) emotional eating, and (5) self-efficacy. This component was specifically designed to target behaviors related to dietary intake and mindful eating, focusing exclusively on these aspects. For each of these 5 areas, patients could complete 2 exercises at any time during the day or week, allowing flexibility and personalization. The exercises were delivered via text or audio, with audio recordings guided by a psychologist. This approach was designed to enhance awareness of both the psychological and physiological aspects of eating by integrating mindful eating, which has been shown to increase awareness of hunger and satiety cues, reduce cravings and emotional eating, and foster greater self-compassion [32,33].

#### Reminders Section

DTxO provided alerts and reminders related to dietary and exercise programs, medication intake, as well as motivational trophies, nutritional education tips, and recipe suggestions**.**

### Placebo Arm (Placebo App)

The placebo app included static content that mimicked the structure of the DTxO interface but did not provide tailored feedback, dynamic adjustments, or interactive features. It was used exclusively to maintain blinding and control for the app usage experience.

Like the intervention group, patients in the placebo group began using the placebo version of DTxO after completing the self-administered IPAQ [28] and psychological questionnaires during their baseline visit. Upon enrollment, patients received a standardized, paper-based dietary plan and a paper-based exercise program. The dietary plan followed general healthy eating principles for weight management and included fixed meal patterns and portion guidance. It was not tailored to individual preferences or dietary needs.

The physical activity section recommended a fixed weekly target of 150 minutes of moderate-intensity aerobic exercise, such as brisk walking, in accordance with current obesity management guidelines [6,34]. This program was nonadaptive, did not change over time, and was not monitored or adjusted based on patient feedback.

Additionally, patients were provided with general suggestions on managing eating behavior during the baseline visit. Unlike the intervention group, no multimedia content or psycho-behavioral exercises were delivered through the placebo app.

Table S2 in Multimedia Appendix 1 provides a summary comparison of the components received by the intervention and placebo groups.

### Digital Dosage

The proposed dose for DTxO was as follows:

- Food section: 10 minutes/week, estimated time for composing the weekly menu and reviewing the proposed content.
- Weight diary section: 2 minutes every 2 weeks, estimated time for recording weight, including measurement on the scale.
- Physical activity section: 35 minutes/day, estimated time needed to complete the prescribed exercises.
- Psycho-behavioral section: 5 minutes/day, estimated time for performing at least one mindfulness exercise from those offered.

When converted to daily usage, the digital device doses were as follows:

- Food section: 1.43 minutes/day
- Weight diary section: 0.14 minutes/day
- Physical activity section: 35 minutes/day
- Psycho-behavioral section: 5 minutes/day.

The total estimated daily adherence for 100% use of DTxO was 42 minutes/day.

For the placebo app, the proposed dosing times were as follows:

- Weight diary section: 2 minutes every 2 weeks for weight recording, including measurement on the scale.
- Physical activity section: 35 minutes/day as a general suggestion for increasing activity.

The total estimated daily adherence for 100% use of the placebo app was 35 minutes/day.

### Statistical Analysis

Sample size was calculated using the “proc power” procedure in SAS software version 9.4 (SAS Institute). A total of 246 patients were randomized 1:1, aiming for 172 completers (86 per treatment group), assuming a 30% dropout rate [35]. This sample size was expected to provide 80% statistical power to detect a 1.5-kg difference in weight loss between groups at 6 months. In previous studies, the efficacy of several pharmacotherapies for obesity was measured as a mean difference of 2 kg or more compared with placebo; in this study, a mean difference of 1.5 kg or more versus placebo was considered clinically meaningful.

The characteristics of enrolled participants were described using median and IQR for continuous variables, or frequency and percentage for categorical variables, both overall and within each study arm.

Comparisons between arms were performed using the Wilcoxon rank sum test for continuous variables, and the chi-square or Fisher exact test for categorical variables, as appropriate.

Absolute and percent changes in weight, BMI, and waist circumference were calculated overall and within each study arm, and tested using the Wilcoxon signed rank test.

Pearson correlation coefficients were calculated to assess the presence of a linear relationship between the 6-month absolute or percent change from baseline in weight and overall adherence.

Overall adherence was calculated as the arithmetic mean of the following parameters:

- The percentage of dietary information completion (once per week was considered 100%).
- The percentage of physical exercise completion (once per day was considered 100%).
- The percentage of weight recording (once every 2 weeks was considered 100%).
- The percentage of mindfulness exercise completion (5 times/week was considered 100%)—this item was only available for the intervention group.

In the analyses, overall adherence was stratified into 2 categories based on its observed effect on weight change: values at or above the 75th percentile were classified as medium-elevated adherence, while values below the 75th percentile were classified as low-scarce adherence. The 75th percentile was calculated from the nontransformed overall adherence values of all enrolled participants.

Univariable and multivariable generalized linear models (GLMs) were fitted to identify factors associated with the 6-month absolute (primary end point) or percent change (secondary end point) in body weight. Slopes with corresponding SEs or 95% CIs were estimated. The multivariable models included the study arm, overall adherence, and covariates with a *P* value of 0.10 or less in univariable regression models, while avoiding multicollinearity among the included covariates.

The 6-month absolute and percent change in weight from baseline was also evaluated using univariable mixed linear models for repeated measures, controlling for clinic sites and with study group as a fixed effect, to account for missing data.

Two-sided *P* values <.05 were considered statistically significant.

All statistical analyses were performed using SAS software (version 9.4; SAS Institute).

### Ethical Considerations

#### Ethics Approval

Ethical approval for the study was granted by the Ethics Committees of both centers (IRCCS Istituto Auxologico Italiano: approval number 2022_04-12_03; Policlinico di Bari, Giovanni XXIII Hospital: approval number 7392).

#### Informed Consent

The informed consent document comprehensively described the study’s objectives, procedures, and participants’ rights and responsibilities to ensure informed and voluntary participation. Participants were encouraged to ask questions and consult health care providers or trusted individuals before enrollment. Importantly, the informed consent explicitly permitted the use of baseline and previously collected data even if participants subsequently withdrew their consent. Participants who declined or withdrew from the study were assured that their care would continue at the highest standard. Written informed consent was obtained from all participants before any study procedures.

#### Privacy and Confidentiality

All participant data were recorded in an electronic case report form, with author SB ensuring accuracy through electronic signatures and maintaining source documentation. Data were pseudonymized through coding, with only the principal investigator and authorized research assistants able to link the code to the participant’s identity when necessary. The investigator permitted monitoring, audits, and regulatory inspections, providing access to source data as required.

#### Compensation

No compensation was provided to participants in this study.

## Results

### Overview

**Figure 2:**
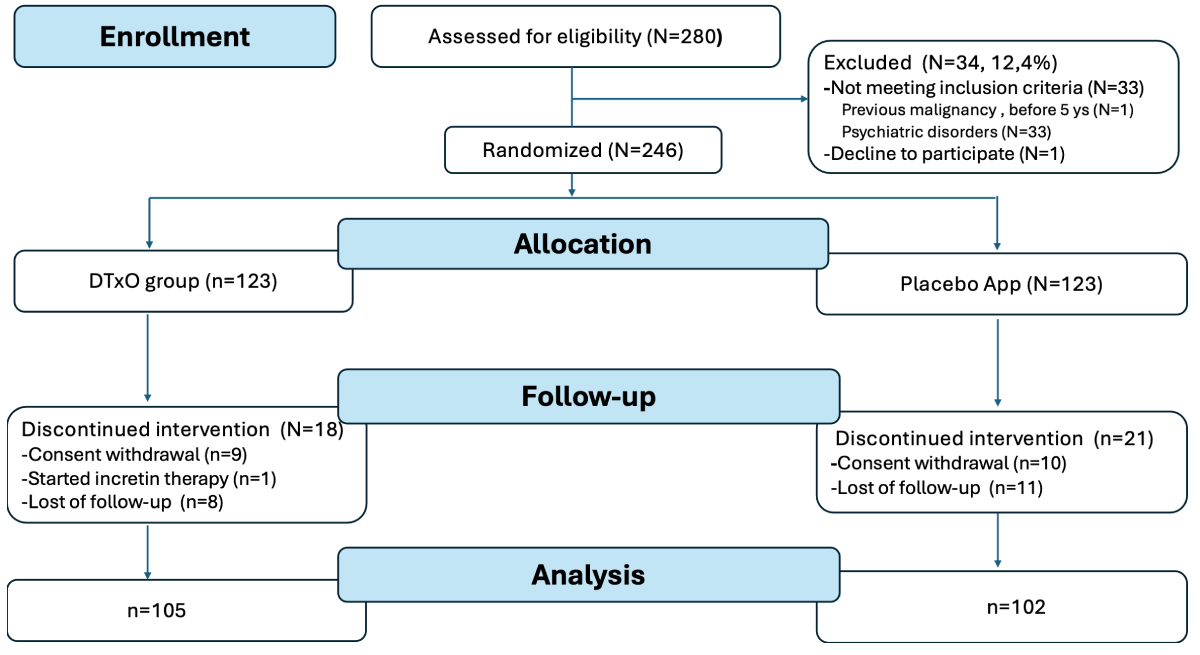
Screening, randomization, and follow-up: The study groups included (1) a placebo app group that received a standardized paper-based diet and exercise plan and used the app solely to complete forms related to diet adherence, exercise, and weight trends, and (2) an intervention group that received the DTxO app with a personalized diet and exercise plan, cognitive behavioral support, reminders, drug intake tracking, and online communication with health care professionals. DTxO: Digital Therapeutics for Obesity.

Of the 280 patients screened for eligibility (Figure 2; also see Multimedia Appendix 2), 246 were randomized to either the DTxO group or the placebo app group (n=123 each). At the 6-month follow-up, 207 participants completed the assessment (105 in the DTxO group and 102 in the Placebo App group), while 39 discontinued: 19 withdrew consent, 1 initiated therapy with a GLP-1 analogue (liraglutide), and 19 were lost to follow-up. Multimedia Appendix 2 presents the CONSORT (Consolidated Standards of Reporting Trials)-EHEALTH checklist.

### Baseline Characteristics

Baseline characteristics of the 246 enrolled participants are summarized in Table 1.

No statistically significant differences between the DTxO and placebo app groups were observed for any baseline characteristics (see Table 1). Most participants were highly educated, consistent with the metropolitan settings of recruitment. Overall (N=246), 1 (0.4%) had completed primary school, 174 (70.7%) had completed high school, 62 (25.2%) held a university degree, and 9 (3.7%) had obtained a master’s degree or PhD, with no differences between study arms. Regarding marital status, 151 (61.4%) participants were married, 78 (31.7%) were single, 16 (6.5%) were separated or divorced, and 1 (0.4%) were widowed, with no significant differences between groups (*P*=.31).

**Table 1:** Baseline characteristics among 246 enrolled patients according to study arm.

Characteristics					Overall (N=246)		DTxO (n=123)		Placebo app (n=123)		*P* value^a^
**Age (years), median (Q1-Q3)**					49.0 (38.0-56.0)		49.0 (40.0-56.0)		51.0 (37.0-56.0)		.69
**Gender, n (%)**											.40
	Male				69 (28.0)		38 (30.9)		31 (25.2)		
	Female				177 (72.0)		85 (69.1)		92 (74.8)		
**Ethnicity, n (%)**											.61
	Black or African American				1 (0.4)		0 (0)		1 (0.8)		
	Hispanic or Latino				2 (0.8)		1 (0.8)		1 (0.8)		
	White				243 (98.8)		122 (99.2)		121 (98.4)		
**Nutritional status and clinical parameters, median (Q1-Q3)**											
	Weight (kg)				98.4 (89.0-107.0)		99.4 (88.5-106.9)		97.5 (89.5-107.0)		.95
	Height (m)				1.7 (1.6-1.7)		1.7 (1.6-1.7)		1.6 (1.6-1.7)		.61
	BMI (kg/m^2^)				35.3 (32.9-38.6)		35.3 (32.8-38.3)		35.3 (33.0-38.8)		.62
	Waist circumference (cm)				111.0 (105.5-118.6)		112.4 (105.5-118.0)		110.0 (105.5-119.2)		.57
	**Degree of obesity, n (%)**										.57
		Grade 1 (BMI<35 kg/m^2^)			116 (47.2)		60 (48.8)		56 (45.5)		
		Grade 2 (BMI>35<40 kg/m^2^)			92 (37.4)		47 (38.2)		45 (36.6)		
		Grade 3 (BMI≥40 kg/m^2^)			38 (15.4)		16 (13.0)		22 (17.9)		
	Systolic blood pressure (mmHg), median (Q1-Q3)				125.0 (120.0-130.0)		125.0 (120.0-130.0)		120.0 (120.0-130.0)		.85
	Diastolic blood pressure (mmHg), median (Q1-Q3)				80.0 (80.0-90.0)		80.0 (80.0-90.0)		80.0 (80.0-85.0)		.53
**Biochemical parameters, median (Q1-Q3)**											
	Fasting glucose (mg/dL)				92.0 (85.0-99.0)		94.0 (86.0-100.0)		91.0 (84.0-98.0)		.07
	Insulin (mU/I)				11.6 (8.7-16.8)		11.7 (8.2-17.0)		11.5 (8.9-16.2)		.99
	Glycated hemoglobin (%)				5.4 (5.2-5.6)		5.4 (5.2-5.7)		5.35 (5.1-5.6)		.45
	HOMA-IR^b^ index				2.6 (1.8-4.0)		2.6 (1.7-4.1)		2.5 (1.9-3.8)		.95
	Total cholesterol (mg/dL)				185.0 (160.0-212.0)		181.0 (160.0-209.0)		186.5 (160.5-216.0)		.34
	High-density lipoprotein cholesterol (mg/dL)				51.0 (43.0-62.0)		50.0 (41.0-59.0)		53.5 (43.5-62.0)		.18
	Low-density lipoprotein cholesterol (mg/dL)				116.0 (96.0-139.0)		116.0 (97.0-134.0)		116.0 (96.0-141.0)		.52
	Triglycerides (mg/dL)				102.0 (74.0-130.0)		103.0 (73.0-126.0)		101.5 (74.5-131.5)		.80
	Estimated glomerular filtration rate (mL/minute)				93.4 (82.8-102.6)		93.5 (84.2-101.9)		93.0 (81.8-104.2)		.83
	Aspartate aminotransferase (U/I)				20.0 (17.0-25.0)		20.0 (16.0-24.0)		21.0 (18.0-26.0)		.03
	Alanine transaminase (U/I)				22.0 (17.0-33.0)		22.0 (16.0-32.0)		23.0 (17.0-36.0)		.42
	Alkaline phosphatase (U/I)				74.0 (62.0-88.0)		70.0 (62.0-85.0)		77.0 (61.5-92.0)		.10
	Gamma-glutamyl transferase (U/I)				20.0 (14.0-32.0)		20.0 (14.0-29.0)		19.0 (14.0-32.5)		.78
	Free thyroxine (pmol/L)				15.2 (14.1-16.9)		15.2 (13.7-17.0)		15.1 (14.2-16.9)		.36
	Thyroid-stimulating hormone (mU/I)				1.8 (1.2-2.5)		1.8 (1.3-2.5)		1.8 (1.2-2.5)		.76
**Lifestyle habits**											
	**Smoke, n (%)**										.95
			Yes	42 (17.1)		21 (17.1)		21 (17.1)			
			No	156 (63.4)		79 (64.2)		77 (62.6)			
			Ex-smoker	48 (19.5)		23 (18.7)		25 (20.3)			
	Adherence to Mediterranean dietary pattern^c^, median (Q1-Q3)				7.0 (6.0-8.0)		7.0 (6.0-8.0)		7.0 (6.0-8.0)		.84
	International Physical Activity Questionnaire (MET-minutes^d^ per week), median (Q1-Q3)				558.0 (350.0-990.0)		630.0 (375.0-1110.0)		525.0 (350.0-900.0)		.08
**Dietary intervention composition, median (Q1-Q3)**											
	Energy (kcal/day)				1398.0 (1277.0-1688.0)		1413.0 (1277.0-1700.0)		1295.0 (1229.0-1688.0)		.22
	Protein (g/kg)				0.7 (0.7-0.8)		0.7 (0.7-0.8)		0.7 (0.7-0.8)		.38
	Carbohydrates (percentage of energy intake)				46.6 (45.7-47.1)		46.6 (45.7-47.1)		46.9 (45.7-47.1)		.42
	Fiber (g/day)				31.0 (29.0-35.0)		31.0 (29.0-34.0)		31.0 (29.0-35.0)		.93
	Lipids (percentage of energy intake)				33.7 (32.8-34.5)		33.7 (32.8-34.5)		34 (32.9-34.4)		.90

^a^By chi-square or Fisher exact test (categorical variables) or Wilcoxon rank sum test (continuous variables).

^b^HOMA-IR index: Homeostatic Model Assessment of Insulin Resistance.

^c^Mediterranean dietary pattern, assessed using a validated 14-item questionnaire [29]. The MeDiet score ranges from 0 to 14. Scores above 9 indicate high adherence, scores below 5 indicate low adherence, and scores between 5 and 9 reflect moderate adherence

^d^MET-minutes: metabolic equivalent of task minutes (calculated as MET value × minutes of activity).

Overall, 95 (38.6%) patients had hypertension (DTxO: 53/123, 43.1%; placebo app: 42/123, 34.1%; *P*=.19), 26 (10.6%) had type 2 diabetes (DTxO: 14/123, 11.4%; placebo app: 12/123, 9.8%; *P*=.84), and 78 (31.7%) had metabolic syndrome (DTxO: 42/123, 34.1%; placebo app: 36/123, 29.3%; *P*=.49), with no significant differences between groups. Furthermore, of the 246 patients, 44 (17.9%) had hepatic steatosis, 11 (4.5%) had obstructive sleep apnea syndrome, and 10 (4.1%) had polycystic ovary syndrome, with no significant differences between groups (hepatic steatosis: *P*=.99; obstructive sleep apnea syndrome: *P*=.99; polycystic ovary syndrome: *P*=.33). Regarding medication use, 91 (37%) patients used antihypertensive drugs (DTxO: 52/123, 42.3%; placebo app: 39/123, 31.7%; *P*=.11), 38 (15.4%) used glucose-lowering drugs (DTxO: 24/123, 19.5%; placebo app: 14/123, 11.4%; *P*=.11), and 18 (7.3%) used lipid-lowering drugs (DTxO: n=10, 8.1%; placebo app: n=8, 6.5%; *P*=.81).

The dietary intervention was comparable between groups in terms of both calorie content and macronutrient composition and distribution.

Table S3 in Multimedia Appendix 1 shows that baseline characteristics were also similar between groups among patients who completed the 6-month follow-up.

### Study Adherence

Table S4 in Multimedia Appendix 1 shows the percentages of overall adherence as well as adherence by dimension, in the overall sample and by study arm.

Overall adherence (all participants: median 24.7%, IQR 11.0%-37.7%) was significantly higher in the DTxO group (median 31.5%, IQR 16.9%-44.5%) compared with that in the placebo app group (median 17.0%, IQR 5.0%-31.9%; *P*<.001). The dimensions with the highest adherence were dietary (DTxO group: median 37.0%, IQR 18.5%-70.4%; placebo app: median 13.0%, IQR 3.7%-44.4%; *P*<.001) and weight recording (DTxO group: median 57.1%, IQR 35.7%-85.7%; placebo app: median 35.7%, IQR 14.3%-64.3%; *P*<.001) in both arms. The mean digital dosage in the DTxO group was 13.3 minutes/day.

### Clinical Outcomes

The absolute and percent changes in weight, BMI, and waist circumference over the 6-month follow-up among the 207 enrolled patients, by study arm, are reported in Table 2.

Both arms achieved a statistically significant absolute and percentage reduction in body weight at the 6-month follow-up, with no significant differences between the DTxO and placebo app groups (*P*=.42 and *P*=.34, respectively). Univariable analysis with GLMs confirmed these findings (estimated mean 6-month absolute change: –3.1, 95% CI –4.3 to –2.3 vs –4.0, 95% CI –5.0 to –3.0 in the DTxO and placebo app groups, respectively, *P*=.34; estimated mean 6-month percent change: –3.3%, 95% CI –4.3% to –2.3% vs –4.3%, 95% CI –5.4% to –3.3% in the DTxO and placebo app groups, respectively; *P*=.17).

A similar trend was observed for the absolute and percent reduction in waist circumference at the 6-month follow-up, although significance was only marginal in the placebo app group (6-month absolute change*: P*=.06; 6-month percentage change: *P*=.08). No significant differences in absolute or percent waist circumference reduction between the DTxO and placebo app groups were detected (*P*=.32 and *P*=.31, respectively).

Similarly, no significant differences were observed in the mean changes of biochemical parameters at 6 months between the 2 groups (Table S5 in Multimedia Appendix 1).

**Table 2:** Weight, BMI, and waist circumference values during the first 6 months of follow-up among 207 enrolled patients with available follow-up according to study arm.

Variable			Overall (N=207)		DTxO (n=105)		Placebo app (n=102)		*P* value^a^		
**Weight (kg), median (Q1-Q3)**											
	Baseline	97.0 (89.0 to 107.1)		99.4 (87.9 to 106.7)		97.3 (89.9 to 107.1)		.72			
	6 months	94.6 (84.0 to 103.1)		96.1 (86.2 to 103.1)		92.6 (82.0 to 103.8)		.20			
	6-month absolute change	–3.4 (–6.0 to –0.7)		–3.2 (–6.0 to –0.9); *P*<.001^b^		–4.0 (–6.9 to –0.5); *P*<.001^b^		.42			
	6-month percentage change	–3.5 (–6.4 to –0.7)		–3.0 (–5.7 to –0.8); *P*<.001^b^		–4.0 (–8.5 to –0.5); *P*<.001^b^		.34			
**BMI (kg/m^2^), median (Q1-Q3)**											
	Baseline	35.0 (32.9 to 38.5)		34.7 (32.6 to 38.2)		35.3 (33.3 to 38.6)		.27			
	6 months	33.8 (30.9 to 36.7)		33.9 (31.1 to 36.2)		33.7 (30.8 to 36.6)		.83			
	6-month absolute change	–1.2 (–2.2 to –0.3)		–1.1 (–2.0 to –0.3); *P*<.001^b^		–1.5 (–2.9 to –0.2); *P*<.001^b^		.33			
	6-month percentage change	–3.4 (–6.4 to –0.7)		–3.0 (–5.7 to –0.8); *P*<.001^b^		–4.0 (–8.5 to –0.5); *P*<.001^b^		.34			
**Waist circumference (cm), median (Q1-Q3)**											
	Baseline	110.0 (105.0 to 118.0)		112.0 (105.5 to 117.6)		109.5 (105.0 to 118.0)		.51			
	6 months	106.9 (100.0 to 114.0)		106.6 (99.1 to 113.2)		108.0 (100.1 to 114.0)		.69			
	6-month absolute change	–5.3 (–13.6 to 4.6)		–6.9 (–14.0 to 4.0); *P*=.002^b^		–5.0 (–12.8 to 5.3); *P*=.06^b^		.32			
	6-month percentage change	–5.0 (–11.6 to 4.2)		–6.4 (–12.4 to 3.8); *P*=.004^b^		–4.6 (–11.3 to 4.7); *P*=.08^b^		.31			

^a^By Wilcoxon rank sum test (continuous variables).

^b^By Wilcoxon signed rank test (continuous variables; test applied only within each study arm).

**Figure 3:**
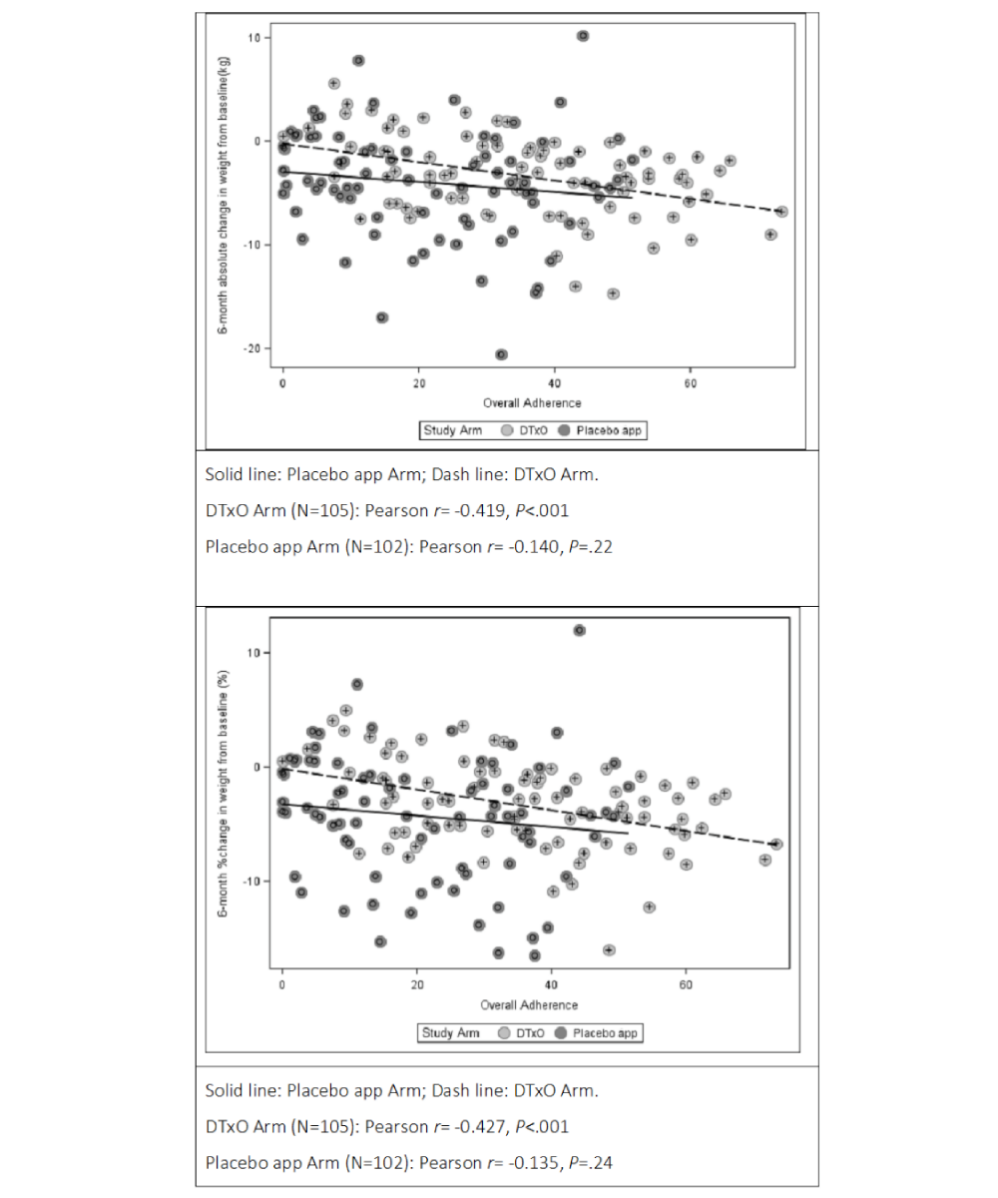
The univariable relationship between adherence and weight loss across study arms, with the corresponding regression lines and correlation coefficients. A high level of treatment adherence was associated with greater clinical performance (ie, greater weight loss). DTxO: Digital Therapeutics for Obesity.

To investigate factors influencing 6-month absolute and percent weight loss, univariable GLMs were calculated, with the findings reported in Tables S6 and S7 in Multimedia Appendix 1. A strong impact of adherence was found on both absolute (β=–.06, SE 0.02, *P*=.01) and percent changes in body weight (β=–.05, SE 0.01, *P*=.01). A marginal effect of the clinical center was also observed for both absolute and percent weight loss. Figure 3 illustrates the univariable relationship between adherence and weight loss according to study arms, with the corresponding regression lines and correlation coefficients; higher treatment adherence was associated with greater clinical performance (ie, greater weight loss).

As these analyses identified adherence as a crucial parameter for treatment success, multivariable analyses considered overall adherence stratified at the third quartile threshold to distinguish truly adherent patients from those with no or modest adherence. The 75th percentile of total app daily usage time corresponded to approximately 40% (equivalent to 16.6 minutes/day): patients with less than 40% daily usage were classified as having low adherence, whereas those with at least 40% of the expected usage time were classified as having medium to high adherence.

Baseline characteristics of patients with overall adherence of 40% or over according to the study arm are described in Table S8 in Multimedia Appendix 1. No statistically significant differences were found between the DTxO-adherent group (n=35) and the placebo app–adherent group (n=10) for any of the considered characteristics.

Among adherent participants, the 6-month mean change in the DTxO group (estimated by a univariable GLM) was –5.4 kg (95% CI –6.8 to –4.0) compared with –1.5 kg (95% CI –4.1 to 1.1) in the placebo app group (*P*=.01). Similarly, the estimated 6-month mean percent change was –5.4% (95% CI –6.8% to –4.0%) in the DTxO group and –1.7% (95% CI –4.3% to 1.0%) in the placebo app group (*P*=.02).

The multivariable GLM (Table S9 in Multimedia Appendix 1), adjusted for clinical site, showed a strong and significant difference in absolute body weight loss at 6 months. The estimated mean absolute change in weight was –7.0 kg (95% CI –9.5 to –4.6) in the DTxO-adherent group and –3.5 kg (95% CI –7.0 to 0.01) in the placebo app–adherent group (*P*=.02). The estimated 6-month mean percent change in weight was –6.3% (95% CI –8.9% to –3.8%) in the DTxO-adherent group and –2.8% (95% CI –6.5% to 0.9%) in the placebo app–adherent group (*P*=.03).

Absolute and percent 6-month changes in weight were also analyzed using mixed linear models for repeated measures (in addition to GLMs), with results summarized in Table S10 in Multimedia Appendix 1. The 6-month weight losses (both absolute and percent) estimated with mixed linear models for repeated measures were consistent with the findings obtained from GLM analyses, both in the overall sample and among adherent patients.

Finally, adherent patients randomized to DTxO showed significant 6-month reductions in BMI and waist circumference (both absolute, *P*=.01, and percent, *P*=.01, changes) compared with the placebo group; however, the analyses did not demonstrate greater reductions in the intervention group relative to the control group (Table S11 in Multimedia Appendix 1).

### Safety and Adverse Events

Overall, 22 of 207 (10.6%) patients reported at least one adverse event (AE): 8 out of 105 (7.6%) in the DTxO group and 14 out of 102 (13.7%) in the placebo app group (*P*=.45).

A total of 14 AEs were reported in the DTxO arm and 18 in the placebo app arm (Table S12 in Multimedia Appendix 1). None of the reported AEs was considered related to the device or the study intervention.

The most frequently reported AE severity was grade 1-2 in both treatment groups. Overall, the most commonly reported AEs were musculoskeletal problems (11/32, 34%), followed by endocrine problems (6/32, 19%), renal and urinary problems (4/32, 13%), respiratory system disorders (4/32, 13%), thromboembolism (3/32, 9%), and other problems (4/32, 13%). In the DTxO group, the most reported AEs were endocrine problems (4/14, 29%), followed by musculoskeletal problems (3/14, 21%), respiratory system disorders (3/14, 21%), and thromboembolism (2/14, 14%). In the placebo app group, the most reported AEs were musculoskeletal problems (8/18, 44%), followed by renal and urinary problems (3/18, 17%), other problems (3/18, 17%), and endocrine problems (2/18, 11%).

Serious AEs were reported in 3 out of 105 (2.9%) patients in the DTxO group (1 renal stone, 1 obstructive renal failure, and 1 renal colic) and 3 out of 102 (2.9%) patients in the placebo app group (1 deep vein thromboses associated with a physical defect, 1 pulmonary embolism [after thrombosis], and 1 COVID-19 infection), for a total of 6 serious AEs.

## Discussion

### Principal Findings

To the best of our knowledge, this is one of the first RCTs to report weight loss in individuals with obesity as a direct result of a multicomponent DTx combining dietary, physical activity, and mindfulness components.

After 6 months, the DTxO group did not show greater weight loss compared with the placebo app group; however, a clear dose-response effect was observed (Figure 3). Patients who engaged with the DTxO for at least 16.7 minutes/day (digital dosage) experienced an average weight loss of 7.0 kg (6.3% reduction), compared with 3.5 kg (2.8% reduction) in the control group. This identified daily digital dosage in the DTxO group led to clinically improved weight loss compared with the placebo app, highlighting the meaningful value of the multicomponent digital algorithm, as digital dosage in the intervention group correlated with meaningful clinical outcomes. Ensuring patient adherence to DTx is intuitively crucial for their effectiveness. The Food and Drug Administration has recently completed a pilot program that outlines guidelines for software certification and highlights the importance of standardized measurements for user adherence, including download counts, installation rates, and activity levels [36]. Our results suggest that while the mindfulness section was included in the digital intervention, it may not have had an impact as significant as the core components of diet, physical activity, and weight monitoring. This indicates that the primary factors driving successful outcomes in the proposed DTxO were diet, physical activity, and weight monitoring, rather than mindfulness. Moreover, while engagement factors in traditional in-person weight loss programs have been extensively studied and considered key to their effectiveness, in this study, the reported outcome can be directly attributed to exposure to DTxO as a standalone intervention. No in-person interaction with the clinical team or personalized coaching (daily, weekly, or monthly) was incorporated alongside the digital intervention.

Weight loss achieved at the 6-month follow-up at the analyzed daily digital dosage of the proposed DTxO as a standalone intervention is clinically significant. Indeed, a 5% reduction in body weight, regardless of BMI category, has been linked to improvements in health metrics such as systolic and diastolic blood pressure, fasting glucose, hemoglobin A_1c_, and high-density lipoprotein cholesterol, and is widely accepted as a clinically significant target for obesity management in recent guidelines [37]. It is also known that even a 3% weight loss can lead to improvements in cardiovascular, metabolic, renal, hepatic, inflammatory, ovulatory, and psychosocial measures that are likely to result in meaningful health benefits [38].

In addition to the weight loss findings, we did not observe significant changes in BMI, waist circumference, or laboratory parameters between groups.

Our findings suggest that people with obesity can achieve greater weight loss compared with a standard paper-based approach with a standalone digital intervention, and that the DTxO described here, when used at a minimum daily digital dosage of 16.6 minutes, is clinically effective. Future studies are needed to explore which factors must be addressed to further enhance patient engagement and broaden the results reported here.

### Comparison With Prior Work

While several RCTs have explored digital support within lifestyle interventions, evidence on the standalone effectiveness of interactive mobile apps—particularly in the absence of in-person clinical interaction—remains inconclusive. A recent review by Kim and Choi [17] of RCTs on digital-based obesity interventions reported mixed results. One study used evidence-based psychological strategies (cognitive behavioral therapy) through a 24-week digital platform in 70 women, with a personalized daily coaching program led by a psychologist. Participants in the digital cognitive behavioral therapy group showed significant weight loss at 8 weeks, but this effect was not sustained at 24 weeks [15]. Similarly, Spring et al [39] found in a 3-group RCT of a smartphone-supported weight loss program with 96 adults with obesity that weight loss did not differ significantly between groups, and a higher percentage of individuals who recorded their progress on paper achieved at least 5% weight loss [39]. This contradicts the assumption that technology-supported weight loss interventions always outperform standard treatments [40]. As discussed by Lugones-Sanchez et al [41], the lack of effectiveness may depend on the type or duration of the intervention. However, we believe that effectiveness in these outcomes, as in weight outcomes, mainly depends on the level of app use, which may encourage users to adopt new, healthier behaviors over time. As the rate of users who sufficiently adhered to the study app was very low, this may explain the lack of effectiveness in showing improved results in key outcomes at 6 months in our study.

Finally, 2 recent RCTs in Europe have evaluated digital interventions for obesity. The Zanadio trial, conducted in Germany, tested a 12-month multimodal app-based intervention incorporating validated components from behavioral science, exercise therapy, and nutrition [42]. Participants in the intervention arm achieved a mean weight loss of approximately 4.5 kg, with a dropout rate of about 15% (18/123, 14.6%). However, the control group continued with usual care without access to a digital tool, limiting the ability to isolate the specific effect of the digital intervention and reducing internal comparability. The Vitadio trial, conducted in the Czech Republic, evaluated a CE-marked Class I medical device that provided a 3-month intensive digital program followed by a 3-month maintenance phase, supported by in-app chat access to a dietitian and gamified educational content [43]. The intervention group achieved a mean weight loss of 5.2 kg along with improvements in metabolic markers. These outcomes were comparable to those in the control group, which received 5 structured in-person counseling sessions and ongoing professional support. However, the presence of intensive human interaction in both arms makes it difficult to isolate the specific contribution of the digital modality.

By contrast, our study addresses a key gap in the literature by employing a rigorously designed, placebo-controlled trial to evaluate the standalone effect of a multicomponent DTx. While differences in follow-up durations prevent direct comparisons of weight loss magnitudes, the use of a digital placebo and the absence of in-person contact minimize confounding from human interaction or device-enhanced motivation. A recent study systematically reviewed the effectiveness of smartphone apps for weight loss at 3 and 6 months, showing that smartphone app–based interventions led to significant weight loss: –1.99 kg at 3 months and –2.80 kg at 6 months. The study also revealed that when a human-based behavioral intervention was added, participants experienced substantially greater weight loss. Additionally, the number or type of app features did not correlate with weight loss outcomes [44].

In our study, we observed high enrollment, with only 1 participant declining to participate, and a low dropout rate (18/123, 14.6%, in the DTxO group and 21/123, 17.1%, in the placebo group), both notably lower than typical dropout rates in standard care interventions. This may reflect a good level of acceptability of digital health tools among people with obesity. A previous survey conducted at IRCCS Auxologico Italiano, which included participants with demographic characteristics similar to those in the DEMETRA study, also reported high patient engagement with digital interventions [45]. While the study did not demonstrate significant differences in weight loss between groups in the overall sample, these findings—particularly the low dropout rates over a 6-month period—suggest that DTxO may hold potential as supportive tools in obesity management, warranting further investigation in longer-term studies. Compliance with lifestyle interventions is known to be a major challenge in treating people living with obesity, even in the context of RCTs [46]. Digital interventions have been associated with lower dropout rates compared with in-person programs [15,17,41]. In our study, dropout rates were similar between the DTxO and placebo groups, possibly due to the presence of self-monitoring features (eg, dietary adherence, physical activity, and weight tracking) within the placebo app, which may have supported sustained participant engagement. While this remains speculative, previous evidence suggests that self-monitoring plays a critical role in promoting adherence and reducing dropout in weight management interventions [47,48].

Physical activity is a cornerstone of obesity management [49,50], and increasing physical activity provides numerous health benefits. Exercise prescriptions should be individualized based on a patient’s physical capacity, exercise history, motivation, and overall health. The recommended dosage is at least 150 minutes/week of moderate-intensity activity, adjusted for physical limitations. In our study, participants in the DTxO group were encouraged to engage in physical activity for an average of 35 minutes/day, with exercise intensity tailored to their baseline fitness levels. The placebo app, by contrast, suggested only general increases in physical activity without specific recommendations. Participants in the DTxO group demonstrated better adherence to the physical activity program, although both groups achieved the recommended activity levels.

Given the insufficient resources available to national health care systems and the increasing demand for obesity treatment due to the obesity “pandemic,” alternative, scalable care delivery models are needed [1]. A growing body of evidence supports the use of digital care interventions to bridge these gaps [17].

This shift will have significant cultural, clinical, and organizational implications for health care providers. DTxO can enhance patient engagement, promote timely access to care, and provide valuable data on patient progress and outcomes. However, the concept of “digital dosage” and the identification of a “list of digital excipients” are essential for ensuring its effectiveness.

It can be hypothesized that the same digital active ingredient—the core algorithm underlying the DTx—may exert varying therapeutic effects depending on the “excipients” included in the intervention. These excipients may include (1) the user interface, (2) the set of modules delivering the therapy, (3) reminders to support adherence, and (4) modules facilitating patient-doctor and patient-patient interactions.

### Strengths and Limitations

#### Strengths

A key strength of this study lies in its robust methodology—a prospective, multicenter, randomized, double-arm, placebo-controlled, single-blind trial comparing a digital therapeutic customized for obesity treatment with a placebo app following a standard care approach. One of the most critical and challenging aspects of designing RCTs for DTx is the creation of an appropriate control group. Specifically, selecting the primary active ingredient of a DTx and designing a corresponding sham control group (an identical DTx platform without the primary active ingredient) presents significant methodological challenges. In this study, the placebo app was designed to mirror the DTxO in terms of user interface and self-monitoring systems but excluded active algorithms or excipients, consistent with previous studies investigating the role of digital support in obesity interventions [17].

Another methodological strength is the study’s adequate statistical power, which enabled the detection of meaningful differences between the 2 arms in 6-month weight loss outcomes.

At randomization, the 2 groups were comparable in sociodemographic, clinical, and obesity-related variables, ensuring the reliability and lack of bias in the results. Moreover, the dropout rate was low and, importantly, well balanced between the 2 study arms.

The innovative design of the DTx intervention is another key strength of this study. The DTxO integrated customized menu options, nutrition education sessions, recipe videos, physical activity exercises tailored to habitual activity levels, and mindfulness modules—an approach not previously developed in combination for obesity management. These components were designed by a multidisciplinary team of endocrinologists, dietitians, psychologists, physiotherapists, and kinesiologists, all with extensive expertise in obesity treatment. This collaborative design is consistent with international guidelines and recommendations for obesity management [6,51].

#### Limitations

This study has several limitations. First, blinding of the researchers was not possible due to the nature of the intervention. Second, the generalizability of the findings may be limited by the recruitment strategy, which targeted individuals with social media accounts and proficiency in the Italian language, as the content was not available in other languages. Additionally, the sample was predominantly middle-aged, with a majority of female participants, all recruited from 2 second-level obesity outpatient clinics located in large cities in northern and southern Italy. We also acknowledge that most participants were White (243/246, 98.7%), which may limit the generalizability of the findings to other ethnic groups. Furthermore, when evaluated only among adherent patients, the primary end point included a very small number of participants in both study groups (35/105, 33.3%, in the DTxO group and 10/102, 9.8%, in the placebo app group). Although the characteristics of adherent versus nonadherent patients did not differ (data not shown), this analysis cannot rule out the possibility of statistical bias that might influence the interpretation of the results.

Finally, participants were required to have a score of 2 or less on the “Depression,” “Anxiety,” and “Psychoticis” subscales of the Symptom Checklist-90-R, a Numeric Rating Scale score of 5 or less for reported pain in the lower limb joints [21], and an Edmonton Obesity Stage of less than 4 [20]. Consequently, it remains unclear whether these findings can be generalized to the broader population of people with obesity, particularly those with significant psychiatric disorders, severe cardiometabolic comorbidities, or physical impairments [52-54].

### Conclusions

This trial demonstrated greater 6-month weight loss in people with obesity who adhered to using the new DTxO for at least 16 minutes/day, compared with patients randomized to the placebo app.

DTxO, integrating dietary, physical activity, and mindfulness components into a multicomponent digital health intervention, offers a promising new approach for weight loss management as a standalone treatment. It could also be combined with other emerging strategies for obesity management, such as new incretin-mimetic drugs and bariatric surgery. Given the importance of adherence as a key factor for therapeutic success in DTx, it will be crucial to focus on strategies that enhance user engagement to maximize the effectiveness of DTxO.

Further research is needed to identify the most suitable candidates for each treatment modality—digital, in-person, or hybrid—taking into account clinical characteristics, demographics, and patient preferences.

Finally, the widespread adoption of DTxO will require health care professionals, including obesity specialists and dietitians, to acquire new competencies, necessitating updates to training curricula. In addition, long-term follow-up studies and cost-effectiveness analyses are essential to fully evaluate the benefits and sustainability of this intervention.

## Appendix

Multimedia Appendix 1 Additional analysis.

Multimedia Appendix 2 CONSORT-eHEALTH checklist (V 1.6.1).

## Abbreviations

AE: adverse event
CONSORT: Consolidated Standards of Reporting Trials
DEMETRA: Digital Therapy to Promote Weight Loss in Patients With Obesity by Increasing Their Adherence to Treatment
DTx: digital therapeutics
DTxO: Digital Therapeutics for Obesity
GLM: generalized linear model
IPAQ: International Physical Activity Questionnaire
RCT: randomized controlled trial

## Data Availability

The study will be registered on ClinicalTrials.gov upon publication and will remain available for at least five years. Aggregated data from the study will be available upon substantiated request to the corresponding author. Code for all statistical analyses may be made available upon substantiated request to the corresponding author.

## References

[1] Bray G, Kim K, Wilding J, World Obesity Federation (2017). Obesity: a chronic relapsing progressive disease process. A position statement of the World Obesity Federation. Obes Rev.

[2] Bray GA (2025). Obesity: a 100 year perspective. Int J Obes (Lond).

[3] Capodaglio P, Castelnuovo G, Brunani A, Vismara L, Villa V, Capodaglio Edda Maria (2010). Functional limitations and occupational issues in obesity: a review. Int J Occup Saf Ergon.

[4] Okunogbe A, Nugent R, Spencer G, Powis J, Ralston J, Wilding J (2022). Economic impacts of overweight and obesity: current and future estimates for 161 countries. BMJ Glob Health.

[5] Yumuk V, Tsigos C, Fried M, Schindler K, Busetto L, Micic D, Toplak H, Obesity Management Task Force of the European Association for the Study of Obesity (2015). European Guidelines for obesity management in adults. Obes Facts.

[6] Gaskin CJ, Cooper K, Stephens LD, Peeters A, Salmon J, Porter J (2024). Clinical practice guidelines for the management of overweight and obesity published internationally: a scoping review. Obes Rev.

[7] Cornier MA (2022). A review of current guidelines for the treatment of obesity. Am J Manag Care.

[8] Hassapidou M, Vlassopoulos A, Kalliostra M, Govers E, Mulrooney H, Ells L, Salas XR, Muscogiuri G, Darleska TH, Busetto L, Yumuk VD, Dicker D, Halford J, Woodward E, Douglas P, Brown J, Brown T (2023). European Association for the Study of Obesity Position Statement on Medical Nutrition Therapy for the Management of Overweight and Obesity in Adults Developed in Collaboration with the European Federation of the Associations of Dietitians. Obes Facts.

[9] Hooker AR, Sagui-Henson SJ, Daubenmier J, Moran PJ, Hartogensis W, Acree M, Kristeller J, Epel ES, Mason AE, Hecht FM (2022). Effects of a mindfulness-based weight loss intervention on long-term psychological well-being among adults with obesity: secondary analyses from the Supporting Health by Integrating Nutrition and Exercise (SHINE) Trial. Mindfulness (N Y).

[10] O'Reilly GA, Cook L, Spruijt-Metz D, Black DS (2014). Mindfulness-based interventions for obesity-related eating behaviours: a literature review. Obes Rev.

[11] Vidal J, Flores L, Jiménez Amanda, Pané Adriana, de Hollanda A (2025). What is the evidence regarding the safety of new obesity pharmacotherapies. Int J Obes (Lond).

[12] Lemstra M, Bird Y, Nwankwo C, Rogers M, Moraros J (2016). Weight loss intervention adherence and factors promoting adherence: a meta-analysis. Patient Prefer Adherence.

[13] Bélisle-Pipon Jean-Christophe, David P (2023). Digital therapies (DTx) as new tools within physicians' therapeutic arsenal: key observations to support their effective and responsible development and use. Pharmaceut Med.

[14] Spring B, Pellegrini CA, Pfammatter A, Duncan JM, Pictor A, McFadden HG, Siddique J, Hedeker D (2017). Effects of an abbreviated obesity intervention supported by mobile technology: the ENGAGED randomized clinical trial. Obesity (Silver Spring).

[15] Kim M, Kim Y, Go Y, Lee S, Na M, Lee Y, Choi S, Choi HJ (2020). Multidimensional cognitive behavioral therapy for obesity applied by psychologists using a digital platform: open-label randomized controlled trial. JMIR Mhealth Uhealth.

[16] Lowe DA, Wu N, Rohdin-Bibby L, Moore AH, Kelly N, Liu YE, Philip E, Vittinghoff E, Heymsfield SB, Olgin JE, Shepherd JA, Weiss EJ (2020). Effects of time-restricted eating on weight loss and other metabolic parameters in women and men with overweight and obesity: the TREAT randomized clinical trial. JAMA Intern Med.

[17] Kim M, Choi HJ (2021). Digital therapeutics for obesity and eating-related problems. Endocrinol Metab (Seoul).

[18] Barteit S, Boudo V, Ouedraogo A, Zabré Pascal, Ouremi L, Sié Ali, Munga S, Obor D, Kwaro D, Huhn S, Bunker A, Sauerborn R, Gunga H, Maggioni MA, Bärnighausen Till (2021). Feasibility, acceptability and validation of wearable devices for climate change and health research in the low-resource contexts of Burkina Faso and Kenya: study protocol. PLoS One.

[19] Castelnuovo G, Capodaglio P, De Amicis R, Gilardini L, Mambrini SP, Pietrabissa G, Cavaggioni L, Piazzolla G, Galeone C, Garavaglia G, Bertoli S, DEMETRA Study Group (2023). Study protocol of a clinical randomized controlled trial on the efficacy of an innovative Digital thErapy to proMote wEighT loss in patients with obesity by incReasing their Adherence to treatment: the DEMETRA study. Front Digit Health.

[20] Sharma AM, Kushner RF (2009). A proposed clinical staging system for obesity. Int J Obes (Lond).

[21] Jensen M, McFarland C (1993). Increasing the reliability and validity of pain intensity measurement in chronic pain patients. Pain.

[22] Gormally J, Black S, Daston S, Rardin D (1982). The assessment of binge eating severity among obese persons. Addict Behav.

[23] Derogatis L (1994). Symptom Checklist-90-Revised SCL-90-R: Administration, Scoring and Procedures Manual.

[24] Stergiou G, Palatini P, Parati G, O'Brien E, Januszewicz A, Lurbe E, Persu A, Mancia G, Kreutz Reinhold, European Society of Hypertension Council and the European Society of Hypertension Working Group on Blood Pressure MonitoringCardiovascular Variability (2021). 2021 European Society of Hypertension practice guidelines for office and out-of-office blood pressure measurement. J Hypertens.

[25] Matthews DR, Hosker JP, Rudenski AS, Naylor BA, Treacher DF, Turner RC (1985). Homeostasis model assessment: insulin resistance and beta-cell function from fasting plasma glucose and insulin concentrations in man. Diabetologia.

[26] Levey AS, Stevens LA, Schmid CH, Zhang Y(, Castro AF, Feldman HI, Kusek JW, Eggers P, Van Lente F, Greene T, Coresh J, CKD-EPI (Chronic Kidney Disease Epidemiology Collaboration) (2009). A new equation to estimate glomerular filtration rate. Ann Intern Med.

[27] Dettwyler KA (2005). Anthropometric standardization reference manual, abridged edition. Edited by Timothy G. Lohman, Alex F. Roche, and Reynaldo Martoll. Champaign, Illinois: Human Kinetic Books. 1991. 90 pp. $16.00 (paper). American J Phys Anthropol.

[28] Mannocci A, Di Thiene D, Del Cimmuto A, Masala D, Boccia A, De Vito E (2010). International Physical Activity Questionnaire: validation and assessment in an Italian sample. ijph.

[29] Martínez-González Miguel Angel, García-Arellano Ana, Toledo E, Salas-Salvadó Jordi, Buil-Cosiales P, Corella D, Covas MI, Schröder Helmut, Arós Fernando, Gómez-Gracia Enrique, Fiol M, Ruiz-Gutiérrez Valentina, Lapetra J, Lamuela-Raventos RM, Serra-Majem L, Pintó Xavier, Muñoz Miguel Angel, Wärnberg Julia, Ros E, Estruch R (2012). A 14-Item Mediterranean Diet Assessment Tool and obesity indexes among high-risk subjects: the PREDIMED trial. PLoS ONE.

[30] (2012). Standard Italiani per la Cura dell' Obesità. Società Italiana di Obesità & Associazione Dietologi Italiani.

[31] Mifflin M, St Jeor S, Hill L, Scott B, Daugherty S, Koh Y (1990). A new predictive equation for resting energy expenditure in healthy individuals. Am J Clin Nutr.

[32] Dunn C, Haubenreiser M, Johnson M, Nordby K, Aggarwal S, Myer S, Thomas C (2018). Mindfulness approaches and weight loss, weight maintenance, and weight regain. Curr Obes Rep.

[33] Morillo-Sarto Héctor, López-Del-Hoyo Yolanda, Pérez-Aranda Adrián, Modrego-Alarcón Marta, Barceló-Soler Alberto, Borao L, Puebla-Guedea Marta, Demarzo M, García-Campayo Javier, Montero-Marin Jesús (2023). 'Mindful eating' for reducing emotional eating in patients with overweight or obesity in primary care settings: a randomized controlled trial. Eur Eat Disord Rev.

[34] Jensen MD, Ryan DH, Apovian CM, Ard JD, Comuzzie AG, Donato KA, Hu FB, Hubbard VS, Jakicic JM, Kushner RF, Loria CM, Millen BE, Nonas CA, Pi-Sunyer FX, Stevens J, Stevens VJ, Wadden TA, Wolfe BM, Yanovski SZ, Jordan HS, Kendall KA, Lux LJ, Mentor-Marcel R, Morgan LC, Trisolini MG, Wnek J, Anderson JL, Halperin JL, Albert NM, Bozkurt B, Brindis RG, Curtis LH, DeMets D, Hochman JS, Kovacs RJ, Ohman EM, Pressler SJ, Sellke FW, Shen W, Smith SC, Tomaselli GF, American College of Cardiology/American Heart Association Task Force on Practice Guidelines, Obesity Society (2014). 2013 AHA/ACC/TOS guideline for the management of overweight and obesity in adults: a report of the American College of Cardiology/American Heart Association Task Force on Practice Guidelines and The Obesity Society. Circulation.

[35] Perna S, Salman M, Gasparri C, Cavioni A, Faliva MA, Mansueto F, Naso M, Patelli Z, Peroni G, Tartara A, Riva A, Petrangolini G, Rondanelli M (2022). Two, six, and twelve-month dropout rate and predictor factors after a multidisciplinary residential program for obesity treatment. a prospective cohort study. Front Nutr.

[36] Lee TT, Kesselheim AS (2018). U.S. Food and Drug Administration Precertification Pilot Program for digital health software: weighing the benefits and risks. Ann Intern Med.

[37] Williamson DA, Bray GA, Ryan DH (2015). Is 5% weight loss a satisfactory criterion to define clinically significant weight loss?. Obesity (Silver Spring).

[38] Dhar D, Packer J, Michalopoulou S, Cruz J, Stansfield C, Viner RM, Mytton OT, Russell SJ (2025). Assessing the evidence for health benefits of low-level weight loss: a systematic review. Int J Obes (Lond).

[39] Spring B, Schneider K, McFadden HG, Vaughn J, Kozak AT, Smith M, Moller AC, Epstein LH, Demott A, Hedeker D, Siddique J, Lloyd-Jones DM (2012). Multiple behavior changes in diet and activity: a randomized controlled trial using mobile technology. Arch Intern Med.

[40] Antoun J, Itani H, Alarab N, Elsehmawy A (2022). The effectiveness of combining nonmobile interventions with the use of smartphone apps with various features for weight loss: systematic review and meta-analysis. JMIR Mhealth Uhealth.

[41] Lugones-Sanchez C, Recio-Rodriguez JI, Agudo-Conde C, Repiso-Gento I, G Adalia E, Ramirez-Manent JI, Sanchez-Calavera MA, Rodriguez-Sanchez E, Gomez-Marcos MA, Garcia-Ortiz L, EVIDENT 3 Investigators (2022). Long-term effectiveness of a smartphone app combined with a smart band on weight loss, physical activity, and caloric intake in a population with overweight and obesity (Evident 3 Study): randomized controlled trial. J Med Internet Res.

[42] Roth L, Ordnung M, Forkmann K, Mehl N, Horstmann A (2023). A randomized-controlled trial to evaluate the app-based multimodal weight loss program zanadio for patients with obesity. Obesity (Silver Spring).

[43] Moravcová Katarína, Sovová Markéta, Ožana J, Karbanová Martina, Klásek Jan, Kolasińska AB, Sovová Eliška (2024). Comparing the efficacy of digital and in-person weight loss interventions for patients with obesity and glycemic disorders: evidence from a randomized non-inferiority trial. Nutrients.

[44] Chew HSJ, Koh WL, Ng JSHY, Tan KK (2022). Sustainability of weight loss through smartphone apps: systematic review and meta-analysis on anthropometric, metabolic, and dietary outcomes. J Med Internet Res.

[45] Carrera A, Zoccarato F, Mazzeo M, Lettieri E, Toletti G, Bertoli S, Castelnuovo G, Fresa E (2023). What drives patients' acceptance of digital therapeutics? Establishing a new framework to measure the interplay between rational and institutional factors. BMC Health Serv Res.

[46] Moroshko I, Brennan L, O'Brien P (2011). Predictors of dropout in weight loss interventions: a systematic review of the literature. Obesity Reviews.

[47] Patel ML, Wakayama LN, Bennett GG (2021). Self-monitoring via digital health in weight loss interventions: a systematic review among adults with overweight or obesity. Obesity (Silver Spring).

[48] Burke LE, Styn MA, Sereika SM, Conroy MB, Ye L, Glanz K, Sevick MA, Ewing LJ (2012). Using mHealth technology to enhance self-monitoring for weight loss: a randomized trial. Am J Prev Med.

[49] Jakicic JM, Rogers RJ, Davis KK, Collins KA (2018). Role of physical activity and exercise in treating patients with overweight and obesity. Clin Chem.

[50] Oppert J, Bellicha A, Ciangura C (2021). Physical activity in management of persons with obesity. Eur J Intern Med.

[51] Chianelli M, Busetto L, Vettor R, Annibale B, Paoletta A, Papini E, Albanese A, Carabotti M, Casarotto D, De Pergola G, Disoteo O E, Grandone I, Medea G, Nisoli E, Raffaelli M, Schiff S, Vignati F, Cinquini M, Gonzalez-Lorenzo M, Fittipaldo V A, Minozzi S, Monteforte M, Tralongo A C, Novizio R, Persichetti A, Samperi I, Scoppola A, Borretta G, Carruba M, Carbonelli M G, De Luca M, Frontoni S, Corradini S G, Muratori F, Attanasio R (2024). Italian guidelines for the management of adult individuals with overweight and obesity and metabolic comorbidities that are resistant to behavioral treatment. J Endocrinol Invest.

[52] Perry C, Guillory TS, Dilks SS (2021). Obesity and psychiatric disorders. Nurs Clin North Am.

[53] Weiss F, Barbuti M, Carignani G, Calderone A, Santini F, Maremmani I, Perugi G (2020). Psychiatric aspects of obesity: a narrative review of pathophysiology and psychopathology. J Clin Med.

[54] Lin Hung-Yen, Huang Chih-Kun, Tai Chi-Ming, Lin Hung-Yu, Kao Yu-Hsi, Tsai Ching-Chung, Hsuan Chin-Feng, Lee Su-Long, Chi Shu-Ching, Yen Yung-Chieh (2013). Psychiatric disorders of patients seeking obesity treatment. BMC Psychiatry.

