# Supplementary material for: Effectiveness of a Digital Therapy on 6-Month Weight Loss in People With Obesity: The Digital Therapy to Promote Weight Loss in Patients With Obesity by Increasing Their Adherence to Treatment (DEMETRA) Randomized Clinical Trial
Source: J Med Internet Res. 2025 Oct 21;27:e72054. doi: 10.2196/72054 (PMC12587017; doi:10.2196/72054)
Supplement: Multimedia Appendix 1 [file jmir_v27i1e72054_app1.docx]

Table S1. Summary of components received by both study groups.

Table S2. Comparison of intervention and placebo group components.

Table S3. Baseline characteristics among 207 enrolled patients with available follow-up according to study arm.

| **Characteristics** | | | **Overall**  **(N=207)** | **DTxO**  **(N=105)** | **Placebo App**  **(N=102)** | ***P* value^a^** |
| --- | --- | --- | --- | --- | --- | --- |
| **Age (years), median (Q1-Q3) ^b^** | | | 50.0 (39.0 - 57.0) | 49.0 (41.0 - 56.0) | 51.0 (37.0 - 57.0) | .79 |
| **Gender, n (%)** | |  |  |  |  | .23 |
|  | Male | | 61 (29.5%) | 35 (33.3%) | 26 (25.5%) |  |
|  | Female | | 146 (70.5%) | 70 (66.7%) | 76 (74.5%) |  |
| **Ethnicity, n (%)** | |  |  |  |  | 1.00 |
|  | Hispanic or Latino | | 2 (1.0%) | 1 (1.0%) | 1 (1.0%) |  |
|  | White | | 205 (99.0%) | 104 (99.0%) | 101 (99.0%) |  |
| **Nutritional Status and Clinical parameters, median (Q1-Q3) ^b^** | | | | | | |
|  | Weight (kg) | | 97.0 (89.0-107.1) | 99.4 (87.9-106.7) | 97.3 (89.9-107.1) | .72 |
|  | Height (m) | | 1.7 (1.6-1.7) | 1.7 (1.6-1.7) | 1.6 (1.6-1.7) | .26 |
|  | Body Mass Index (kg/m^2^) | | 35.0 (32.9-38.5) | 34.7 (32.6-38.2) | 35.3 (33.3-38.6) | .27 |
|  | Waist circumference (cm) | | 110.0 (105.0 - 118.0) | 112.0 (105.5 - 117.6) | 109.5 (105.0 - 118.0) | .51 |
|  | Degree of obesity, n (%) | |  |  |  | .52 |
|  | | Grade 1 (BMI<35 kg/m^2^) | 101 (48.8%) | 54 (51.4%) | 47 (46.1%) |  |
|  | | Grade 2 (BMI>35 - <40 kg/m^2^) | 77 (37.2%) | 39 (37.1%) | 38 (37.3%) |  |
|  | | Grade 3 (BMI≥40 kg/m^2^) | 29 (14.0%) | 12 (11.4%) | 17 (16.7%) |  |
|  | Sistolic blood pressure (mm Hg) | | 120.0 (120.0 - 130.0) | 125.0 (120.0 - 130.0) | 120.0 (120.0 - 130.0) | .88 |
|  | Diastolic blood pressure (mm Hg) | | 80.0 (80.0 - 85.0) | 80.0 (80.0 - 90.0) | 80.0 (80.0 - 85.0) | .46 |
| **Biochemical parameters, median (Q1-Q3) ^b^** | | | | | | |
|  | Fasting glucose (mg/dL) | | 92.0 (86.0 – 99.0) | 94.0 (87.0 - 100.0) | 91.0 (84.0 - 97.0) | .14 |
|  | Insulin (mU/I) | | 11.4 (8.2 - 15.8) | 11.6 (8.0 - 16.5) | 11.3 (8.7 - 15.5) | .96 |
|  | Glycated hemoglobin (%) | | 5.4 (5.2 - 5.6) | 5.4 (5.2 - 5.6) | 5.4 (5.2 - 5.6) | .85 |
|  | HOMA-IR index | | 2.6 (1.7 - 3.9) | 2.6 (1.7 – 4.0) | 2.5 (1.8 - 3.8) | .91 |
|  | Total cholesterol (mg/dL) | | 183.0 (157.0 - 210.0) | 179.0 (158.0 - 206.0) | 185.0 (157.0 - 214.0) | .44 |
|  | High-density lipoprotein cholesterol (mg/dL) | | 51.0 (42.0 - 61.0) | 49.0 (41.0 - 59.0) | 52.0 (44.0 - 62.0) | .19 |
|  | Low-density lipoprotein cholesterol (mg/dL) | | 114.0 (93.0 - 138.0) | 112.0 (96.0 - 132.0) | 115.0 (93.0 - 139.0) | .71 |
|  | Triglycerides (mg/dL) | | 101.0 (73.0 - 126.0) | 101.0 (73.0 - 125.0) | 100.0 (73.0 - 126.0) | .76 |
|  | Estimated Glomerular Filtration Rate (ml/min) | | 93.0 (82.8 – 102.5) | 93.3 (83.0 – 101.6) | 92.5 (82.8 – 104.5) | .67 |
|  | Aspartate aminotransferase (U/I) | | 20.0 (17.0 - 25.0) | 19.0 (16.0 - 24.0) | 23.0 (19.0 - 28.0) | .002 |
|  | Alanine transaminase (U/I) | | 23.0 (17.0 - 35.0) | 22.0 (16.0 - 31.0) | 25.0 (17.0 - 39.0) | .08 |
|  | Alkaline phosphatase (U/I) | | 71.0 (61.0 - 88.0) | 69.0 (61.0 - 85.0) | 77.0 (61.0 - 92.0) | .18 |
|  | Gamma-glutamyl transferase (U/I) | | 19.5 (14.0 - 33.0) | 20.0 (13.0 - 28.0) | 19.0 (14.0 - 34.0) | .65 |
|  | Free Thyroxine (pmol/L) | | 15.4 (14.2 – 17.0) | 15.4 (13.9 - 17.0) | 15.2 (14.2 - 17.0) | .67 |
|  | Thyroid stimulating hormone (mU/I) | | 1.8 (1.2 - 2.6) | 1.8 (1.2 - 2.6) | 1.8 (1.3 - 2.5) | .89 |
| **Lifestyle Habits, median (Q1-Q3) ^b^** | | | | | | |
|  | Smoke, n (%) | |  |  |  | .68 |
|  | | Yes | 32 (15.5%) | 15 (14.3%) | 17 (16.7%) |  |
|  | | No | 134 (64.7%) | 71 (67.6%) | 63 (61.8%) |  |
|  | | Ex-smoker | 41 (19.8%) | 19 (18.1%) | 22 (21.6%) |  |
|  | Adherence to Mediterranean dietary pattern^c^  (score 0-14) | | 7.0 (6.0-8.0) | 7.0 (6.0-8.0) | 7.0 (6.0-8.0) | .78 |
|  | Physical Activity Leveld (MET-min per week) | | 540.0 (350.0-984.0) | 630.0 (375.0-1060.0) | 480.0 (350.0-840.0) | .09 |
| **Dietary information composition, median (Q1-Q3) ^b^** | | | | | | |
|  | Energy (kcal/day) | | 1413.0 (1277.0 – 1688.0) | 1472.0 (1233.0 – 1711.0) | 1398.0 (1277.0 – 1586.0) | .56 |
|  | Protein (g/kg) | | 0.7 (0.7 - 0.8) | 0.7 (0.7 - 0.8) | 0.7 (0.7 - 0.8) | .86 |
|  | Carbohydrates (%/EI) | | 46.7 (45.7 - 47.1) | 46.9 (45.9 - 47.7) | 46.6 (45.7 - 47.1) | .04 |
|  | Fiber (g/day) | | 31.0 (29.0 - 35.0) | 32.0 (29.0 - 35.0) | 31 (29.0 - 34.0) | .53 |
|  | Lipids (%/EI) | | 33.7 (32.9 - 34.5) | 33.7 (32.8 - 34.5) | 34.0 (32.9 - 34.5) | .32 |

^a^ by chi-square or Fisher’s exact test (categorical variables) or Wilcoxon rank-sum test (continuous variables).

^b^ Q1, first quartile; Q3, third quartile.

^C^ Mediterranean dietary pattern, assessed using a validated 14-item questionnaire [29]. The MeDiet score ranges from 0 to 14. Scores above 9 indicate high adherence, scores below 5 indicate low adherence, and scores between 5 and 9 reflect moderate adherence

Abbreviations

BMI: body mass index (kg/m^2^)

HOMA-IR index: Homeostatic Model Assessment of Insulin Resistance

MET-min: Metabolic Equivalent of Task minutes (calculated as MET value × minutes of activity)

%EI: % of energy intake

**Table S4. Study adherence in the overall sample and according to study arm.**

| **Adherence, median (Q1-Q3) ^a^** | **Overall**  **(N = 207)** | **DTxO**  **(N = 105)** | **Placebo App**  **(N = 102)** | ***P* value ^b^** |
| --- | --- | --- | --- | --- |
| Dietary adherence (%) | 29.6 (7.4 - 51.9) | 37.0 (18.5 - 70.4) | 13.0 (3.7 - 44.4) | <.001 |
| Physical activity adherence (%) | 8.7 (1.6 - 22.8) | 15.2 (6.0 - 34.2) | 3.3 (0.5 - 15.1) | <.001 |
| Weight information recording adherence (%) | 50 (28.6 - 78.6) | 57.1 (35.7 - 85.7) | 35.7 (14.3 - 64.3) | <.001 |
| Mindfulness exercises adherence (%) | -^c^ | 0 (0 - 3.7) | -^c^ | - |
| Overall adherence ^d^ (%) | 24.7 (11.0 - 37.7) | 31.5 (16.9 - 44.5) | 17.0 (5.0 - 31.9) | <.001 |

^a^ Q1, first quartile; Q3, third quartile.

^b^ Wilcoxon rank-sum test.

^c^ Overall adherence was defined by the arithmetic mean of the previous 4 adherence components.

^d^ This item was available only for the DTxO group.

**Table S5. Laboratory parameters values during the first 6 months of follow-up among 207 enrolled patients with available follow-up according to study arm.**

| **Characteristic, median (Q1-Q3) ^a^** | | | **Overall**  **(N=207)** | **DtxO**  **(N=105)** | **Placebo App**  **(N=102)** | ***P* value ^b^** |
| --- | --- | --- | --- | --- | --- | --- |
| Baseline fasting glucose (mg/dL) | |  | 92.0 (86.0 - 99.0) | 94.0 (87.0 - 100.0) | 91.0 (84.0 - 97.0) | .14 |
| 6-month fasting glucose (mg/dL) | |  | 90.0 (86.0 - 97.0) | 89.0 (84.0 - 96.0) | 91.0 (87.0 - 97.0) | .24 |
| 6-month change fasting glucose (mg/dL) | |  | -1.0 (-10.0 - 8.0)  *P*=.47 ^c^ | -3.0 (-11.0 - 8.0)  *P*=.21 ^c^ | 1.0 (-8.0 - 8.0)  *P*=.71 ^c^ | .22 |
| Baseline insulin (mU/I) | |  | 11.4 (8.2 - 15.8) | 11.6 (8.0 - 16.5) | 11.3 (8.7 - 15.5) | .96 |
| 6-month insulin (mU/I) | |  | 12.0 (8.7 – 17.0) | 12.0 (9.0 - 17.9) | 11.9 (8.6 - 16.1) | .47 |
| 6-month change insulin (mU/I) | |  | -0.2 (-5.3 - 5.0)  *P*=.61 ^c^ | -0.4 (-5.1 - 6.6)  *P*=.60 ^c^ | 0 (-5.3 - 4.6)  *P*=.83 ^c^ | .85 |
| Baseline glycated hemoglobin (%) | |  | 5.4 (5.2 - 5.6) | 5.4 (5.2 - 5.6) | 5.4 (5.2 - 5.6) | .85 |
| 6-month glycated hemoglobin (%) | |  | 5.4 (5.2 - 5.6) | 5.4 (5.1 - 5.5) | 5.4 (5.2 - 5.6) | .27 |
| 6-month change glycated hemoglobin (%) | |  | 0 (-0.5 - 0.4)  *P*=.59 ^c^ | -0.1 (-0.6 - 0.4)  *P*=.26 ^c^ | 0.1 (-0.3 - 0.4)  *P*=.77 ^c^ | .40 |
| Baseline HOMA-IR index | |  | 2.6 (1.7 - 3.9) | 2.6 (1.7 – 4.0) | 2.5 (1.8 - 3.8) | .91 |
| Baseline HOMA-IR index, n (%) | |  |  |  |  | .88 |
|  | ≤3.5 | | 149 (72.0%) | 75 (71.4%) | 74 (72.5%) |  |
|  | >3.5 | | 58 (28.0%) | 30 (28.6%) | 28 (27.5%) |  |
| 6-month HOMA-IR index | |  | 2.7 (1.9 - 3.8) | 2.7 (1.9 - 3.9) | 2.7 (1.8 - 3.6) | .61 |
| 6-month HOMA-IR index, n (%) | |  |  |  |  | .17 |
|  | ≤3.5 | | 107 (68.2%) | 49 (62.8%) | 58 (73.4%) |  |
|  | >3.5 | | 50 (31.8%) | 29 (37.2%) | 21 (26.6%) |  |
| 6-month change HOMA-IR index | |  | -0.1 (-1.2 - 1.5)  *P*=.94 ^c^ | -0.2 (-1.2 – 2.0)  *P*=.57 ^c^ | 0.02 (-1.4 - 0.9)  *P*=.70 ^c^ | .62 |
| Baseline AST (U/I) | |  | 20.0 (17.0 - 25.0) | 19.0 (16.0 - 24.0) | 23.0 (19.0 - 28.0) | .002 |
| 6-month AST (U/I) | |  | 19.0 (16.0 - 23.0) | 18.0 (16.0 - 22.0) | 20.0 (16.0 - 24.0) | .24 |
| 6-month change AST (U/I) | |  | -1.0 (-7.0 - 2.0)  *P*=.005 ^c^ | -1.0 (-5.0 - 2.0)  *P*=.13 ^c^ | -2.0 (-9.0 - 2.0)  *P*=.02 ^c^ | .39 |
| Baseline ALT (U/I) | |  | 23.0 (17.0 - 35.0) | 22.0 (16.0 - 31.0) | 25.0 (17.0 - 39.0) | .08 |
| 6-month ALT (U/I) | |  | 20.0 (15.5 - 27.0) | 18.0 (15.0 - 27.0) | 22.0 (16.0 - 27.0) | .16 |
| 6-month change ALT (U/I) | |  | -3.0 (-14.0 - 6.0)  *P*=.008 ^c^ | -4.0 (-10.0 - 6.0)  *P*=.09 ^c^ | -2.5 (-17.0 - 6.0)  *P*=.04 ^c^ | .72 |
| Baseline alkaline phosphatase (U/I) | |  | 71.0 (61.0 - 88.0) | 69.0 (61.0 - 85.0) | 77.0 (61.0 - 92.0) | .18 |
| 6-month alkaline phosphatase (U/I) | |  | 72.5 (60.0 - 89.5.0) | 68.0 (56.0 - 84.0) | 77.0 (63.0 - 94.0) | .04 |
| 6-month change alkaline phosphatase (U/I) | |  | -1.0 (-18.0 - 18.0)  *P*=.772 ^c^ | -1.0 (-14.0 - 15.0)  *P*=.96 ^c^ | -2.0 (-20.0 - 25.0)  *P*=.64 ^c^ | .79 |
| Baseline GGT (U/I) | |  | 19.5 (14.0 - 33.0) | 20.0 (13.0 - 28.0) | 19.0 (14.0 - 34.0) | .65 |
| 6-month GGT (U/I) | |  | 19.0 (13.0 - 25.5.0) | 18.0 (13.0 - 24.0) | 19.0 (13.0 - 26.0) | .30 |
| 6-month change GGT (U/I) | |  | -2.0 (-11.0 - 5.0)  *P*=.04 ^c^ | -2.0 (-8.0 - 3.0)  *P*=.12 ^c^ | -3.5 (-13.0 - 6.0)  *P*=.16 ^c^ | .82 |
| Baseline total cholesterol (mg/dL) | |  | 183.0 (157.0 - 210.0) | 179.0 (158.0 - 206.0) | 185.0 (157.0 - 214.0) | .44 |
| 6-month total cholesterol (mg/dL) | |  | 185.0 (156.5 - 205.5) | 191.0 (160.0 - 212.0) | 177.0 (154.0 - 199.0) | .08 |
| 6-month change total cholesterol (mg/dL) | |  | -4.0 (-40.0 - 33.0)  *P*=.54 ^c^ | 13.0 (-28.0 - 43.0)  *P*=.16 ^c^ | -14.0 (-56.0 - 20.0)  *P*=.04 ^c^ | .01 |
| Baseline HDL cholesterol (mg/dL) | |  | 51.0 (42.0 - 61.0) | 49.0 (41.0 - 59.0) | 52.0 (44.0 - 62.0) | .19 |
| 6-month HDL cholesterol (mg/dL) | |  | 52.0 (44.0 - 61.0) | 54.0 (45.0 - 64.0) | 51.0 (44.0 - 58.0) | .11 |
| 6-month change HDL cholesterol (mg/dL) | |  | 3.0 (-13.0 - 13.0)  *P*=.85 ^c^ | 5.0 (-11.0 - 14.0)  *P*=.08 ^c^ | -6.5 (-17.0 - 11.0)  *P*=.15^c^ | .04 |
| Baseline LDL cholesterol (mg/dL) | |  | 114.0 (93.0 - 138.0) | 112.0 (96.0 - 132.0) | 115.0 (93.0 - 139.0) | .71 |
| 6-month LDL cholesterol (mg/dL) | |  | 113.0 (91.5 - 134.5.0) | 120.0 (94.0 - 139.0) | 111.0 (86.0 - 130.0) | .18 |
| 6-month change LDL cholesterol (mg/dL) | |  | -2.0 (-34.0 - 32.0)  *P*=.77 ^c^ | 5.0 (-20.0 - 33.0)  *P*=.20 ^c^ | -14.5 (-38.0 - 25.0)  *P*=.12 ^c^ | .04 |
| Baseline triglycerides (mg/dL) | |  | 101.0 (73.0 - 126.0) | 101.0 (73.0 - 125.0) | 100.0 (73.0 - 126.0) | .76 |
| 6-month triglycerides (mg/dL) | |  | 92.5 (70.5 - 138.5) | 93.0 (67.0 - 138.0) | 92.0 (74.0 - 139.0) | .74 |
| 6-month change triglycerides (mg/dL) | |  | 0 (-43.0 - 42.0)  *P*=.64 ^c^ | -5.0 (-45.0 - 32.0)  *P*=.35 ^c^ | 5.0 (-41.0 - 44.0)  *P*=.80 ^c^ | .42 |
| Baseline eGFR (ml/min) | |  | 93.0 (82.8 – 102.5) | 93.3 (83.0 – 101.6) | 92.5 (82.8 – 104.5) | .67 |
| 6-month eGFR (ml/min) | |  | 90.6 (77.7 - 99.4) | 90.5 (76.1 - 97.9) | 90.6 (79.9 - 100.8) | .64 |
| 6-month change eGFR (ml/min) | |  | -2.5 (-18.7 – 14.0)  *P*=.206 ^c^ | -2.0 (-18.7 – 14.0)  *P*=.44 ^c^ | -2.6 (-19.9 - 14.6)  *P*=.33 ^c^ | 1.00 |
| Baseline FT4 (pmol/L) | |  | 15.4 (14.2 – 17.0) | 15.4 (13.9 - 17.0) | 15.2 (14.2 - 17.0) | .67 |
| 6-month FT4 (pmol/L) | |  | 15.6 (13.9 - 17.1) | 15.6 (13.8 - 17.0) | 15.5 (14.3 - 17.1) | .66 |
| 6-month change FT4 (pmol/L) | |  | -0.1 (-2.1 - 2.1)  *P*=.92 ^c^ | 0.3 (-1.8 - 1.8)  *P*=.71 ^c^ | -0.2 (-2.2 - 2.1)  *P*=.80 ^c^ | .66 |
| Baseline TSH (mU/I) | |  | 1.8 (1.2 - 2.6) | 1.8 (1.2 - 2.6) | 1.8 (1.3 - 2.5) | .89 |
| 6-month TSH (mU/I) | |  | 2 (1.4 - 2.8) | 2.1 (1.5 - 2.7) | 2.0 (1.3 - 2.9) | .38 |
| 6-month change TSH (mU/I) | |  | 0.3 (-0.8 - 1.2)  *P*=.09 ^c^ | 0.3 (-0.7 - 1.2)  *P*=.13 ^c^ | 0.02 (-0.8 - 1.3)  *P*=.35 ^c^ | .70 |

^a^ Q1, first quartile; Q3, third quartile.

^b^ by Wilcoxon rank-sum test (continuous variables).

^c^ by Wilcoxon signed-rank test (continuous variables).

**Table S6. Univariable generalized linear model on the association between potential risk factors and absolute change in body weight after 6 months among 207 patients.**

| **Characteristic** | **Category** | **β-coefficient (slope) with Standard error** | ***P* value** |
| --- | --- | --- | --- |
| Study Arm | DtxO | 0.69 (0.72) | .34 |
|  | Placebo App | Ref^a^ |  |
| Clinical centre | Center 1 | 1.55 (0.84) | .07 |
|  | Center 2 | Ref^a^ |  |
| Gender | Male | -1.12 (0.77) | .15 |
|  | Female | Ref^a^ |  |
| Ethnicity | Latin or Hispanic | 2.83 (3.28) | .39 |
|  | White | Ref^a^ |  |
| Education degree | High school first level | 3.25 (3.34) | .33 |
|  | High school second level | -4.05 (3.27) | .22 |
|  | University degree | -4.94 (3.30) | .14 |
|  | Master | -0.35 (4.18) | .93 |
|  | PhD | Ref^a^ |  |
| Marital status | Married | -0.65 (4.65) | .89 |
|  | Single | 0.11 (4.67) | .98 |
|  | Separated or divorced | -1.35 (4.83) | .78 |
|  | Widow | Ref^a^ |  |
| Smoke | Yes | 1.43 (1.25) | .25 |
|  | No | -0.54 (0.89) | .54 |
|  | Ex-smoker | Ref^a^ |  |
| Diabetes mellitus | No | -0.28 (0.96) | .77 |
|  | Yes | Ref^a^ |  |
| Metabolic syndrome | No | 0.94 (0.78) | .23 |
|  | Yes | Ref^a^ |  |
| Overall adherence (4 items) | Per 1 more unit | -0.06 (0.02) | .005 |
| Baseline BMI | Per 1 kg/m^2^ higher | 0.23 (0.10) | .03 |
| Baseline waist circumference | Per 1 cm greater | -0.01 (0.04) | .70 |
| Age | Per 1 year older | -0.05 (0.03) | .11 |
| Baseline fasting glucose | Per 1mg/dl higher | 0.03 (0.04) | .44 |
| Baseline insulin | Per 1 mU/I higher | 0.04 (0.07) | .54 |
| Baseline HOMA-IR index | Per 1 more unit | 0.22 (0.24) | .35 |
| Baseline aspartate aminotransferase | Per 1 U/I higher | -0.19 (0.06) | .001 |
| Baseline alanine transaminase | Per 1 U/I higher | -0.03 (0.03) | .20 |
| Baseline alkaline phosphatase | Per 1 U/I higher | 0.008 (0.02) | .66 |
| Baseline gamma-glutamyl transferase | Per 1 U/I higher | -0.01 (0.03) | .69 |
| Baseline Estimated Glomerular Filtration Rate | Per 1 ml/min higher | 0.03 (0.03) | .20 |
| Baseline total cholesterol | Per 1mg/dl higher | 0.004 (0.01) | .71 |
| Baseline triglycerides | Per 1mg/dl higher | 0.003 (0.007) | .68 |
| Baseline free thyroxine | Per 1 pmol/L higher | 0.22 (0.16) | .15 |
| Baseline thyroid stimulating hormone | Per 1 mU/I higher | -0.14 (0.33) | .68 |

^a^ Ref, reference.

**Table S7. Univariable generalized linear model on the association between potential risk factors with % change in body weight after 6 months among 207 patients.**

| **Characteristic** | **Category** | **β-coefficient (slope) with Standard error** | ***P* value** |
| --- | --- | --- | --- |
| Study Arm | DtxO | 1.02 (0.74) | .17 |
|  | Placebo App | Ref^a^ |  |
| Clinical centre | Center 1 | 1.63 (0.87) | .06 |
|  | Center2 | Ref^a^ |  |
| Gender | Male | -0.55 (0.80) | .49 |
|  | Female | Ref^a^ |  |
| Ethnicity | Latin or Hispanic | 3.86 (3.39) | .26 |
|  | White | Ref^a^ |  |
| Education degree | High school first level | -3.39 (3.45) | .33 |
|  | High school second level | -4.26 (3.39) | .21 |
|  | University degree | -5.12 (3.41) | .14 |
|  | Master | -0.42 (4.32) | .92 |
|  | PhD | Ref^a^ |  |
| Marital status | Married | -1.29 (4.81) | .79 |
|  | Single | -0.57 (4.83) | .91 |
|  | Separated or divorced | -1.83 (5.00) | .72 |
|  | Widow | Ref^a^ |  |
| Smoke | Yes | 1.50 (1.29) | .25 |
|  | No | -0.28 (0.93) | .77 |
|  | Ex-smoker | Ref^a^ |  |
| Diabetes mellitus | No | -0.17 (0.99) | .86 |
|  | Yes | Ref^a^ |  |
| Metabolic syndrome | No | 1.28 (0.80) | .11 |
|  | Yes | Ref^a^ |  |
| Overall adherence (4 items) | Per 1 more unit | -0.05 (0.01) | .01 |
| Baseline BMI | Per 1 kg/m^2^ higher | 0.31 (0.10) | .004 |
| Baseline waist circumference | Per 1 cm greater | 0.02 (0.04) | .51 |
| Age | Per 1 year older | -0.06 (0.03) | .08 |
| Baseline fasting glucose | Per 1mg/dl higher | 0.03 (0.04) | .54 |
| Baseline insulin | Per 1 mU/I higher | 0.06 (0.07) | .41 |
| Baseline HOMA-IR index | Per 1 more unit | 0.22 (0.24) | .36 |
| Baseline aspartate aminotransferase | Per 1 U/I higher | -0.17 (0.06) | .003 |
| Baseline alanine transaminase | Per 1 U/I higher | -0.03 (0.03) | .27 |
| Baseline alkaline phosphatase | Per 1 U/I higher | 0.006 (0.02) | .73 |
| Baseline gamma-glutamyl transferase | Per 1 U/I higher | -0.01 (0.03) | .72 |
| Baseline Estimated Glomerular Filtration Rate | Per 1 ml/min higher | 0.04 (0.03) | .13 |
| Baseline total cholesterol | Per 1mg/dl higher | 0.005 (0.01) | .65 |
| Baseline triglycerides | Per 1mg/dl higher | 0.004 (0.008) | .63 |
| Baseline free thyroxine | Per 1 pmol/L higher | 0.21 (0.16) | .20 |
| Baseline thyroid stimulating hormone | Per 1 mU/I higher | -0.14 (0.35) | .69 |

^a^ Ref, reference.

**Table S8. Baseline characteristics among 45 enrolled patients with overall adherence ≥40% according to study arm.**

| **Characteristic** | | | | **Overall**  **(n=45)** | **DtxO**  **(n=35)** | **Placebo App**  **(n=10)** | ***P* value ^a^** |
| --- | --- | --- | --- | --- | --- | --- | --- |
| **Age (years), median (Q1-Q3) ^b^** | | | | 52.0 (42.0 – 57.0) | 53.0 (45.0 – 58.0) | 46.0 (37.0 – 55.0) | .17 |
| **Gender, n (%)** | | | |  |  |  | .66 |
|  | | Male | | 9 (20.0%) | 8 (22.9%) | 1 (10.0%) |  |
|  | | Female | | 36 (80.0%) | 27 (77.1%) | 9 (90.0%) |  |
| **Ethnicity, n (%)** | | | |  |  |  | - |
|  | | White | | 45 (100%) | 35 (100%) | 10 (100%) |  |
| **Nutritional Status and Clinical Paramaters, median (Q1-Q3) ^b^** | | | | | | | |
|  | | Weight (kg) | | 98.4 (88.9 – 108.9) | 98.5 (90 - 110.3) | 91.5 (85.6 – 104.5) | .52 |
|  | | Height (m) | | 1.7 (1.6-1.7) | 1.7 (1.6-1.7) | 1.6 (1.6-1.7) | .27 |
|  | | Body Mass Index (kg/m^2^) | | 35.4 (32.8 – 38.3) | 34.5 (32.6 – 38.3) | 35.9 (33.5 – 40.5) | .56 |
|  | | Waist circumference (cm) | | 112.3 (104.2 – 117.0) | 113.0 (104.2 - 117.6) | 109.2 (100.4 - 116.5) | .56 |
|  | | Degree of obesity, n (%) | |  |  |  | .21 |
|  | | | Grade 1 (BMI<35 kg/m^2^) | 22 (48.9%) | 18 (51.4%) | 4 (40.0%) |  |
|  | | | Grade 2 (BMI>35 - <40 kg/m^2^) | 17 (37.8%) | 14 (40.0%) | 3 (30.0%) |  |
|  | | | Grade 3 (BMI≥40 kg/m^2^) | 6 (13.3%) | 3 (8.6%) | 3 (30.0%) |  |
|  | | Sistolic blood pressure (mm Hg) | | 125.0 (120.0 – 135.0) | 125.0 (120.0 – 135.0) | 125.0 (120.0 – 140.0) | .48 |
|  | | Diastolic blood pressure (mm Hg) | | 80.0 (80.0 – 90.0) | 80.0 (80.0 – 90.0) | 82.5 (80.0 – 90.0) | .59 |
| **Biochemical parameters, median (Q1-Q3) ^b^** | | | | | | | |
|  | | Fasting glucose (mg/dL) | | 92.0 (84.0 - 96.5) | 92.0 (84.0 – 96.0) | 91.5 (84.0 – 105.0) | .77 |
|  | | Insulin (mU/I) | | 10.8 (7.5 - 15.5) | 12.1 (7.0 - 16.3) | 9.8 (8.3 - 13.7) | .87 |
|  | | Glycated hemoglobin (%) | | 5.4 (5.2 - 5.7) | 5.5 (5.2 - 5.7) | 5.4 (5.1 - 5.6) | .66 |
|  | | HOMA-IR index | | 2.4 (1.6 - 3.9) | 2.7 (1.5 - 3.9) | 2.2 (1.8 - 4.2) | .73 |
|  | | Total cholesterol (mg/dL) | | 186.5 (164.0 – 206.0) | 182.5 (167.0 – 204.0) | 198.5 (153.0 – 225.0) | .40 |
|  | | High-density lipoprotein cholesterol (mg/dL) | | 55.0 (43.5 - 60.5) | 54.5 (40.0 – 59.0) | 55.0 (46.0 – 62.0) | .40 |
|  | | Low-density lipoprotein cholesterol (mg/dL) | | 116.0 (101.0 - 128.5) | 115.5 (101.0 – 128.0) | 118.5 (102.0 – 153.0) | .59 |
|  | | Triglycerides (mg/dL) | | 96.0 (69.5 – 133.0) | 87.5 (68.0 – 114.0) | 129.5 (88.0 – 140.0) | .28 |
|  | | Estimated Glomerular Filtration Rate (ml/min) | | 92.9 (82.8 – 97.5) | 91.3 (78.8 – 97.5) | 94.1 (91.4 – 98.1) | .26 |
|  | | Aspartate aminotransferase (U/I) | | 20.5 (16.0 – 24.0) | 20.5 (16.0 – 24.0) | 21.5 (16.0 – 28.0) | .61 |
|  | | Alanine transaminase (U/I) | | 22.0 (16.0 – 35.0) | 22.0 (16.0 – 32.0) | 21.5 (15.0 – 42.0) | .78 |
|  | | Alkaline phosphatase (U/I) | | 71.0 (59.0 - 87.5) | 71.0 (57.0 – 85.0) | 69.0 (59.0 – 90.0) | .83 |
|  | | Gamma-glutamyl transferase (U/I) | | 19.5 (13.5 - 27.5) | 20.0 (14.0 – 27.0) | 19.5 (13.0 – 38.0) | .92 |
|  | | Free Thyroxine (pmol/L) | | 16.4 (15.0 - 17.8) | 16.1 (14.7 - 17.9) | 16.7 (16.3 - 17.6) | .32 |
|  | | Thyroid stimulating hormone (mU/I) | | 1.7 (1.2 - 2.7) | 1.6 (1.1 - 2.6) | 1.92 (1.6 - 3.2) | .28 |
| **Lifestyle Habits, median (Q1-Q3) ^b^** | | | | | | | |
|  | | Smoke, n (%) | |  |  |  | .78 |
|  | | | Yes | 6 (13.3%) | 4 (11.4%) | 2 (20.0%) |  |
|  | | | No | 29 (64.4%) | 23 (65.7%) | 6 (60.0%) |  |
|  | | | Ex-smoker | 10 (22.2%) | 8 (22.9%) | 2 (20.0%) |  |
|  | Adherence to Mediterranean dietary patternc (score 0-14) | | | 7.0 (7.0 – 8.0) | 7.0 (6.0 – 8.0) | 8.0 (8.0 – 8.0) | .02 |
|  | Physical Activity Leveld (MET-min per week) | | | 630.0 (420.0 – 1260.0) | 630.0 (450.0 – 1260.0) | 848.0 (420.0 – 1260.0) | .94 |
| **Dietary intervention composition, median (Q1-Q3) ^b^** | | | | | | | |
|  | | Energy (kcal/day) | | 1413.0 (1233.0 - 1688.0) | 1398.0 (1233.0 - 1495.0) | 1975.0 (1495.0 - 2096.0) | .21 |
|  | | Protein (g/kg) | | 0.7 (0.7 - 0.8) | 0.7 (0.7 - 0.8) | 0.8 (0.7 - 0.8) | .44 |
|  | | Carbohydrates (%/EI) | | 47.1 (46.2 - 47.9) | 47.1 (46.3 - 47.9) | 46.5 (45.6 - 47.6) | .37 |
|  | | Fiber (g/day) | | 31.0 (29.0 - 34.0) | 31.0 (29.0 - 32.0) | 37.0 (30.0 - 39.5) | .17 |
|  | | Lipids (%/EI) | | 33.5 (32.8 - 34.2) | 33.5 (32.8 - 34.2) | 33.9 (32.9 - 34.4) | .51 |

^a^ by chi-square or Fisher’s exact test (categorical variables) or Wilcoxon rank-sum test (continuous variables).

^b^ Q1, first quartile; Q3, third quartile.

^C^ Mediterranean dietary pattern, assessed using a validated 14-item questionnaire [29]. The MeDiet score ranges from 0 to 14. Scores above 9 indicate high adherence, scores below 5 indicate low adherence, and scores between 5 and 9 reflect moderate adherence

Abbreviations

BMI: body mass index (kg/m^2^)

HOMA-IR index: Homeostatic Model Assessment of Insulin Resistance

MET-min: Metabolic Equivalent of Task minutes (calculated as MET value × minutes of activity)

%EI: % of energy intake

**Table S9.** Multivariable generalized linear model on change in weight after 6 months among 45 patients with overall adherence ≥40%.

| **Covariate** | **Category** | **Model 1 on 6-month mean absolute change (kg)** | | | | **Model 2 on 6-month mean % change (%)** | | | | |
| --- | --- | --- | --- | --- | --- | --- | --- | --- | --- | --- |
|  |  | **β-coefficient (slope)** | ***P* value** | **Estimated 6-month mean change (95%CI) ^b^** | **Estimated mean difference between groups** | **β-coefficient (slope)** | ***P* value** | **Estimated 6-month mean change (95%CI) ^b^** | **Estimated mean difference between groups** |  |
| **Study Arm** | **DTxO** | -3.5 | .02 | -7.0 (-9.5/4.6) | -3.5(-6.4/0.6) | -3.5 | .03 | -6.3(-8.9/-3.8) | -3.5(-6.6/-0.5) |  |
|  | **Placebo App** | Ref^a^ | - | -3.5 (-7.0/0.01) | - | Ref^a^ | - | -2.8(-6.5/0.9) | - |  |
| **Clinical Site** | **Center 1** | 4.0 | .11 | -3.3(-4.7/-1.8) | 4.0(-0.9/ 8.8) | 2.2 | .38 | -3.4(-5.0/-1.9) | 2.2(-2.9/ 7.3) |  |
|  | **Center 2** | Ref^a^ | - | -7.2(-12.1/-2.4) | - | Ref^a^ | - | -5.7(-10.8/0.6) | - |  |

^a^ Ref, reference;

^b^ 95%CI, 95% confidence interval.

**Table S10.** Trend of absolute and percent 6-month change in weight by univariable Mixed Linear Models for Repeated Measures (MLMRM).

| **Panel A**  **N=207 (overall sample)** | DtxO | | | Placebo App | | | Difference between study arms | | |
| --- | --- | --- | --- | --- | --- | --- | --- | --- | --- |
|  | Estimated mean | 95% Confidence Interval | *P* value | Estimated mean | 95% Confidence Interval | p-value | Estimated mean | 95% Confidence Interval | *P* value |
| Absolute change in weight from baseline (Kg) | -3.21 | -3.88, -2.56 | <.001^a^ | -3.62 | -4.55, -2.69 | <.001^a^ | 0.34 | -0.51, 1.20 | .42^b^ |
| Percent change in weight from baseline | -3.05 | -3.75, -2.35 | <.001^a^ | -3.41 | -4.39, -2.44 | <.001^a^ | 0.64 | 0.27, 1.48 | .12^b^ |
|  |  |  |  |  |  |  |  |  |  |
| **Panel B**  **N=45 (adherent patients)** | DtxO | | | Placebo App | | | Difference between study arms | | |
|  | Estimated mean | 95% Confidence Interval | *P* value | Estimated mean | 95% Confidence Interval | p-value | Estimated mean | 95% Confidence Interval | *P* value |
| Absolute change in weight from baseline (Kg) | -5.38 | -6.66, -4.10 | <.001^a^ | -1.52 | -5.26, 2.22 | <.38^a^ | -2.38 | -4.18, -0.57 | .01^b^ |
| Percent change in weight from baseline | -4.53 | -5.67, -3.39 | <.001^a^ | -3.62 | -6.38, -0.41 | <.03^a^ | -2.32 | -4.15, -0.48 | .01^b^ |

^a^ controlling for clinic sites

^b^ controlling for clinic sites and with study arm as fixed effect.

**Table S11.** Weight, BMI, waist circumference values during the first 6 months of follow-up among 45 enrolled patients with overall adherence ≥40% according to study arm.

| **Variable** | | **Overall**  **(N=45)** | **DtxO**  **(N=35)** | **Placebo APP**  **(N=10)** | ***P* value ^a^** |
| --- | --- | --- | --- | --- | --- |
| **Weight (kg), median (Q1-Q3) ^c^** | | | | | |
|  | Baseline | 98.4 (88.9 – 108.9) | 98.5 (90.0 - 110.3) | 91.5 (85.6 – 104.5) | .52 |
|  | 6-month | 94.8 (83.7 – 102.0) | 94.8 (83.9 – 101.7) | 93.1 (83.5 – 102.7) | .89 |
|  | 6-month  absolute change | -4 (-7.3 - -2) | -4 (-7.4 - -2.8)  *P*<.001 ^b^ | -2.8 (-4.5 - 0.3)  *P*=.28 ^b^ | .08 |
|  | 6-month % change | -4.4 (-6.9 - -2.2) | -4.6 (-7.6 - -2.7)  *P*<.0001 ^b^ | -3.0 (-4.3 - 0.3)  *P*=.23 ^b^ | .06 |
| **BMI (kg/m^2^), median (Q1-Q3) ^c^** | | | | | |
|  | Baseline | 35.4 (32.8 – 38.3) | 34.5 (32.6 – 38.3) | 35.9 (33.5 – 40.5) | .56 |
|  | 6-month | 33.9 (30.8 – 36.3) | 33.2 (30.7 – 35.9) | 34.8 (31.4 – 40.1) | .20 |
|  | 6-month  absolute change | -1.5 (-2.5 - -0.8) | -1.6 (-2.6 - -1.1)  *P*<.0001 ^b^ | -1.1 (-1.6 - 0.1)  *P*=.23 ^b^ | .06 |
|  | 6-month  % change | -4.4 (-6.9 - -2.3) | -4.6 (-7.6 - -2.7)  *P*<.0001 ^b^ | -3.0 (-4.3 - 0.3)  *P*=.23 ^b^ | .06 |
| **Waist circumference (cm), median (Q1-Q3) ^c^** | | | | | |
|  | Baseline | 112.3 (104.2 – 117.0) | 113.0 (104.2 - 117.6) | 109.2 (100.4 - 116.5) | .56 |
|  | 6-month | 103.0 (99.0 – 109.0) | 104.3 (99.0 - 111.0) | 100.2 (97.8 - 109.0) | .74 |
|  | 6-month absolute change | -9.0 (-16.2 - 3.2) | -9.3 (-17.4 – 2.0)  *P*=.006 ^b^ | -8.2 (-16.2 - 9.8)  *P*=.69 ^b^ | .59 |
|  | 6-month % change | -8.2 (-14.1 - 3.0) | -8.4 (-15.1 - 1.7)  *P*=.008 ^b^ | -7.7 (-14.1 - 10.2)  *P*=.81 ^b^ | .56 |

^a^ by Wilcoxon rank-sum test (continuous variables).

^b^ by Wilcoxon signed rank test.

^c^ Q1, first quartile; Q3, third quartile.

**Table S12.** Adverse events in the overall sample and according to study arm.

| **AE category, n (.0%)** | | **Overall (N = 32)** | | | **DtxO (N = 14)** | | | **Placebo App (N = 18)** | | |
| --- | --- | --- | --- | --- | --- | --- | --- | --- | --- | --- |
|  |  | **Any grade** | **Grade 1-2** | **Grade 3-4** | **Any grade** | **Grade 1-2** | **Grade 3-4** | **Any grade** | **Grade 1-2** | **Grade 3-4** |
|  |  |  |  |  |  |  |  |  |  |  |
| **Musculoskeletal Problems** | | 11 (34.4%) | 10 | 1 | 3 (21.0%) | 3 | 0 | 8 (44.0%) | 7 | 1 |
|  | Acute lumbosciatica | 1 (3.0%) | 1 | 0 | 1 (7.0%) | 1 | 0 | 0 (0.0%) | 0 | 0 |
|  | Bilateral knee pain | 1 (3.0%) | 1 | 0 | 0 (0.0%) | 0 | 0 | 1 (6.0%) | 1 | 0 |
|  | Cervical protrusion | 1 (3.0%) | 0 | 1 | 0 (0.0%) | 0 | 0 | 1 (6.0%) | 0 | 1 |
|  | Cervicobrachialgia | 1 (3.0%) | 1 | 0 | 0 (0.0%) | 0 | 0 | 1 (6.0%) | 1 | 0 |
|  | Left ankle fracture | 1 (3.0%) | 1 | 0 | 0 (0.0%) | 0 | 0 | 1 (6.0%) | 1 | 0 |
|  | Left hand scaphoid fracture | 1 (3.0%) | 1 | 0 | 0 (0.0%) | 0 | 0 | 1 (6.0%) | 1 | 0 |
|  | Left knee pain | 1 (3.0%) | 1 | 0 | 1 (7.0%) | 1 | 0 | 0 (0.0%) | 0 | 0 |
|  | Left sciatica from paramedian L4-L5 disc herniation | 1 (3.0%) | 1 | 0 | 0 (0.0%) | 0 | 0 | 1 (6.0%) | 1 | 0 |
|  | Low back pain | 1 (3.0%) | 1 | 0 | 0 (0.0%) | 0 | 0 | 1 (6.0%) | 1 | 0 |
|  | Right knee pain | 1 (3.0%) | 1 | 0 | 1 (7.0%) | 1 | 0 | 0 (0.0%) | 0 | 0 |
|  | Sciatica | 1 (3.0%) | 1 | 0 | 0 (0.0%) | 0 | 0 | 1 (6.0%) | 1 | 0 |
| **Endocrine Problems** | | 6 (18.8%) | 5 | 1 | 4 (29.0%) | 3 | 1 | 2 (11.0%) | 2 | 0 |
|  | Chronic myeloproliferative neoplasm of the nose | 1 (3.0%) | 1 | 0 | 1 (7.0%) | 1 | 0 | 0 (0.0%) | 0 | 0 |
|  | Diagnosis of diabetes | 1 (3.0%) | 1 | 0 | 0 (0.0%) | 0 | 0 | 1 (6.0%) | 1 | 0 |
|  | Elevated response atrial fibrillation | 1 (3.0%) | 0 | 1 | 1 (7.0%) | 0 | 1 | 0 (0.0%) | 0 | 0 |
|  | Hypothyroidism | 1 (3.0%) | 1 | 0 | 0 (0.0%) | 0 | 0 | 1 (6.0%) | 1 | 0 |
|  | Hypothyroidism finding | 1 (3.0%) | 1 | 0 | 1 (7.0%) | 1 | 0 | 0 (0.0%) | 0 | 0 |
|  | Subacute thyroiditis due to viral infection | 1 (3.0%) | 1 | 0 | 1 (7.0%) | 1 | 0 | 0 (0.0%) | 0 | 0 |
| **Renal and Urinary Problems** | | 4 (12.5%) | 4 | 0 | 1 (7.0%) | 1 | 0 | 3 (17.0%) | 3 | 0 |
|  | Bilateral calculosis | 1 (3.0%) | 1 | 0 | 0 (0.0%) | 0 | 0 | 1 (6.0%) | 1 | 0 |
|  | Obstructive renal failure | 1 (3.0%) | 1 | 0 | 0 (0.0%) | 0 | 0 | 1 (6.0%) | 1 | 0 |
|  | Renal colic | 1 (3.0%) | 1 | 0 | 0 (0.0%) | 0 | 0 | 1 (6.0%) | 1 | 0 |
|  | Right renal colic | 1 (3.0%) | 1 | 0 | 1 (7.0%) | 1 | 0 | 0 (0.0%) | 0 | 0 |
| **Respiratory System Disorders** | | 4 (12.5%) | 3 | 1 | 3 (21.0%) | 2 | 1 | 1 (6.0%) | 1 | 0 |
|  | Acute bronchitis | 1 (3.0%) | 1 | 0 | 0 (0.0%) | 0 | 0 | 1 (6.0%) | 1 | 0 |
|  | Asthma | 1 (3.0%) | 0 | 1 | 1 (7.0%) | 0 | 1 | 0 (0.0%) | 0 | 0 |
|  | COVID | 1 (3.0%) | 1 | 0 | 1 (7.0%) | 1 | 0 | 0 (0.0%) | 0 | 0 |
|  | Laryngotracheitis | 1 (3.0%) | 1 | 0 | 1 (7.0%) | 1 | 0 | 0 (0.0%) | 0 | 0 |
| **Thromboembolism** | | 3 (9.4%) | 2 | 1 | 2 (14.0%) | 1 | 1 | 1 (6.0%) | 1 | 0 |
|  | Deep vein thrombosis associated with physical defect | 1 (3.0%) | 1 | 0 | 0 (0.0%) | 0 | 0 | 1 (6.0%) | 1 | 0 |
|  | Deep vein thrombosis of the lower right limb | 1 (3.0%) | 0 | 1 | 1 (7.0%) | 0 | 1 | 0 (0.0%) | 0 | 0 |
|  | Subsequent pulmonary embolism (after thrombosis) | 1 (3.0%) | 1 | 0 | 1 (7.0%) | 1 | 0 | 0 (0.0%) | 0 | 0 |
| **Others** | | 4 (12.5%) | 3 | 1 | 1 (7.0%) | 1 | 0 | 3 (17.0%) | 2 | 1 |
|  | Angioedema | 1 (3.0%) | 1 | 0 | 0 (0.0%) | 0 | 0 | 1 (6.0%) | 1 | 0 |
|  | CPK elevation | 1 (3.0%) | 1 | 0 | 1 (7.0%) | 1 | 0 | 0 (0.0%) | 0 | 0 |
|  | Proctorrhagia | 1 (3.0%) | 1 | 0 | 0 (0.0%) | 0 | 0 | 1 (6.0%) | 1 | 0 |
|  | Voluntary termination of pregnancy (pharmacological) | 1 (3.0%) | 0 | 1 | 0 (0.0%) | 0 | 0 | 1 (6.0%) | 0 | 1 |
