# Supplementary material for: Effectiveness of a Digital Therapy on 6-Month Weight Loss in People With Obesity: The Digital Therapy to Promote Weight Loss in Patients With Obesity by Increasing Their Adherence to Treatment (DEMETRA) Randomized Clinical Trial
Source: J Med Internet Res. 2025 Oct 21;27:e72054. doi: 10.2196/72054 (PMC12587017; doi:10.2196/72054)
Supplement: Multimedia Appendix 2 [file jmir_v27i1e72054_app2.pdf]

# CONSORT-EHEALTH (V 1.6.1) - Submission/Publication Form

The CONSORT-EHEALTH checklist is intended for authors of randomized trials evaluating web-based and Internet-based applications/interventions, including mobile interventions, electronic games (incl multiplayer games), social media, certain telehealth applications, and other interactive and/or networked electronic applications. Some of the items (e.g. all subitems under item 5 - description of the intervention) may also be applicable for other study designs.

The goal of the CONSORT EHEALTH checklist and guideline is to be
a) a guide for reporting for authors of RCTs,
b) to form a basis for appraisal of an ehealth trial (in terms of validity)

CONSORT-EHEALTH items/subitems are MANDATORY reporting items for studies published in the Journal of Medical Internet Research and other journals / scientific societies endorsing the checklist.

Items numbered 1., 2., 3., 4a., 4b etc are original CONSORT or CONSORT-NPT (non-pharmacologic treatment) items.
Items with Roman numerals (i., ii, iii, iv etc.) are CONSORT-EHEALTH extensions/clarifications.

As the CONSORT-EHEALTH checklist is still considered in a formative stage, we would ask that you also RATE ON A SCALE OF 1-5 how important/useful you feel each item is FOR THE PURPOSE OF THE CHECKLIST and reporting guideline (optional).

Mandatory reporting items are marked with a red *.
In the textboxes, either copy & paste the relevant sections from your manuscript into this form - please include any quotes from your manuscript in QUOTATION MARKS,
or answer directly by providing additional information not in the manuscript, or elaborating on why the item was not relevant for this study.

YOUR ANSWERS WILL BE PUBLISHED AS A SUPPLEMENTARY FILE TO YOUR PUBLICATION IN JMIR AND ARE CONSIDERED PART OF YOUR PUBLICATION (IF ACCEPTED).
Please fill in these questions diligently. Information will not be copyedited, so please use proper spelling and grammar, use correct capitalization, and avoid abbreviations.

DO NOT FORGET TO SAVE AS PDF _AND_ CLICK THE SUBMIT BUTTON SO YOUR ANSWERS ARE IN OUR DATABASE !!!

Citation Suggestion (if you append the pdf as Appendix we suggest to cite this paper in the caption):
Eysenbach G, CONSORT-EHEALTH Group
CONSORT-EHEALTH: Improving and Standardizing Evaluation Reports of Web-based and Mobile Health Interventions
J Med Internet Res 2011;13(4):e126

URL: http://www.jmir.org/2011/4/e126/
doi: 10.2196/jmir.1923
PMID: 22209829

**simona.bertoli2013@gmail.com** Cambia account

Bozza non salvata

Non condiviso

* Indica una domanda obbligatoria

Your name *

First Last

Simona Bertoli

Primary Affiliation (short), City, Country *

University of Toronto, Toronto, Canada

University of Milan, Italy

Your e-mail address *

abc@gmail.com

simona.bertoli@unimi.it

Title of your manuscript *

Provide the (draft) title of your manuscript.

Effectiveness of a digital therapy on 6-month weight loss in people living with obesity: the DEMETRA Randomized Clinical Trial

Name of your App/Software/Intervention *

If there is a short and a long/alternate name, write the short name first and add the long name in brackets.

Demetra study

Evaluated Version (if any)

e.g. "V1", "Release 2017-03-01", "Version 2.0.27913"

V1 2025-04-15

Language(s) *

What language is the intervention/app in? If multiple languages are available, separate by comma (e.g. "English, French")

Italian

URL of your Intervention Website or App

e.g. a direct link to the mobile app on app in appstore (itunes, Google Play), or URL of the website. If the intervention is a DVD or hardware, you can also link to an Amazon page.

La tua risposta

URL of an image/screenshot (optional)

La tua risposta

Accessibility *

Can an enduser access the intervention presently?

- ○ access is free and open
- ◉ access only for special usergroups, not open
- ○ access is open to everyone, but requires payment/subscription/in-app purchases
- ○ app/intervention no longer accessible
- ○ Altro:

Primary Medical Indication/Disease/Condition *

e.g. "Stress", "Diabetes", or define the target group in brackets after the condition, e.g. "Autism (Parents of children with)", "Alzheimers (Informal Caregivers of)"

Obesity

Primary Outcomes measured in trial *

comma-separated list of primary outcomes reported in the trial

Weight loss

Secondary/other outcomes

Are there any other outcomes the intervention is expected to affect?

Waist circumference, Body mass index

Recommended "Dose" *

What do the instructions for users say on how often the app should be used?

- (selected) Approximately Daily
- Approximately Weekly
- Approximately Monthly
- Approximately Yearly
- "as needed"
- Altro:

Approx. Percentage of Users (starters) still using the app as recommended after 3 months *

- (selected) unknown / not evaluated
- 0-10%
- 11-20%
- 21-30%
- 31-40%
- 41-50%
- 51-60%
- 61-70%
- 71%-80%
- 81-90%
- 91-100%
- Altro:

Overall, was the app/intervention effective? *

- ○ yes: all primary outcomes were significantly better in intervention group vs control
- ◉ partly: SOME primary outcomes were significantly better in intervention group vs control
- ○ no statistically significant difference between control and intervention
- ○ potentially harmful: control was significantly better than intervention in one or more outcomes
- ○ inconclusive: more research is needed
- ○ Altro:

Article Preparation Status/Stage *

At which stage in your article preparation are you currently (at the time you fill in this form)

- ○ not submitted yet - in early draft status
- ○ not submitted yet - in late draft status, just before submission
- ○ submitted to a journal but not reviewed yet
- ◉ submitted to a journal and after receiving initial reviewer comments
- ○ submitted to a journal and accepted, but not published yet
- ○ published
- ○ Altro:

Journal *

If you already know where you will submit this paper (or if it is already submitted), please provide the journal name (if it is not JMIR, provide the journal name under "other")

- ( ) not submitted yet / unclear where I will submit this
- (x) Journal of Medical Internet Research (JMIR)
- ( ) JMIR mHealth and UHealth
- ( ) JMIR Serious Games
- ( ) JMIR Mental Health
- ( ) JMIR Public Health
- ( ) JMIR Formative Research
- ( ) Other JMIR sister journal
- ( ) Altro:

Is this a full powered effectiveness trial or a pilot/feasibility trial? *

- ( ) Pilot/feasibility
- (x) Fully powered

Manuscript tracking number *

If this is a JMIR submission, please provide the manuscript tracking number under "other" (The ms tracking number can be found in the submission acknowledgement email, or when you login as author in JMIR. If the paper is already published in JMIR, then the ms tracking number is the four-digit number at the end of the DOI, to be found at the bottom of each published article in JMIR)

- ( ) no ms number (yet) / not (yet) submitted to / published in JMIR
- (x) Altro: JMIR ms#72054

## TITLE AND ABSTRACT

### 1a) TITLE: Identification as a randomized trial in the title

1a) Does your paper address CONSORT item 1a? *

I.e does the title contain the phrase "Randomized Controlled Trial"? (if not, explain the reason under "other")

- (selected) yes
- Altro:

1a-i) Identify the mode of delivery in the title

Identify the mode of delivery. Preferably use “web-based” and/or “mobile” and/or “electronic game” in the title. Avoid ambiguous terms like “online”, “virtual”, “interactive”. Use “Internet-based” only if Intervention includes non-web-based Internet components (e.g. email), use “computer-based” or “electronic” only if offline products are used. Use “virtual” only in the context of “virtual reality” (3-D worlds). Use “online” only in the context of “online support groups”. Complement or substitute product names with broader terms for the class of products (such as “mobile” or “smart phone” instead of “iphone”), especially if the application runs on different platforms.

| | 1 | 2 | 3 | 4 | 5 | |
|---|---|---|---|---|---|---|
| subitem not at all important | ○ | ○ | ● | ○ | ○ | essential |

Cancella selezione

Does your paper address subitem 1a-i? *

Copy and paste relevant sections from manuscript title (include quotes in quotation marks "like this" to indicate direct quotes from your manuscript), or elaborate on this item by providing additional information not in the ms, or briefly explain why the item is not applicable/relevant for your study

Digital Therapeutic Intervention

1a-ii) Non-web-based components or important co-interventions in title

Mention non-web-based components or important co-interventions in title, if any (e.g., “with telephone support”).

| | 1 | 2 | 3 | 4 | 5 | |
|---|---|---|---|---|---|---|
| subitem not at all important | ○ | ○ | ● | ○ | ○ | essential |

Cancella selezione

Does your paper address subitem 1a-ii?

Copy and paste relevant sections from manuscript title (include quotes in quotation marks "like this" to indicate direct quotes from your manuscript), or elaborate on this item by providing additional information not in the ms, or briefly explain why the item is not applicable/relevant for your study

Digital Therapeutic Intervention

1a-iii) Primary condition or target group in the title

Mention primary condition or target group in the title, if any (e.g., “for children with Type I Diabetes”) Example: A Web-based and Mobile Intervention with Telephone Support for Children with Type I Diabetes: Randomized Controlled Trial

| | 1 | 2 | 3 | 4 | 5 | |
|---|---|---|---|---|---|---|
| subitem not at all important | ○ | ○ | ○ | ○ | ○ | essential |

Does your paper address subitem 1a-iii? *

Copy and paste relevant sections from manuscript title (include quotes in quotation marks "like this" to indicate direct quotes from your manuscript), or elaborate on this item by providing additional information not in the ms, or briefly explain why the item is not applicable/relevant for your study

In people living with obesity

1b) ABSTRACT: Structured summary of trial design, methods, results, and conclusions

NPT extension: Description of experimental treatment, comparator, care providers, centers, and blinding status.

1b-i) Key features/functionalities/components of the intervention and comparator in the METHODS section of the ABSTRACT

Mention key features/functionalities/components of the intervention and comparator in the abstract. If possible, also mention theories and principles used for designing the site. Keep in mind the needs of systematic reviewers and indexers by including important synonyms. (Note: Only report in the abstract what the main paper is reporting. If this information is missing from the main body of text, consider adding it)

| | 1 | 2 | 3 | 4 | 5 | |
|---|---|---|---|---|---|---|
| subitem not at all important | ○ | ○ | ○ | ○ | ◉ | essential |

Cancella selezione

Does your paper address subitem 1b-i? *

Copy and paste relevant sections from the manuscript abstract (include quotes in quotation marks "like this" to indicate direct quotes from your manuscript), or elaborate on this item by providing additional information not in the ms, or briefly explain why the item is not applicable/relevant for your study

The DTxO App offered a holistic approach by integrating personalized diet plans, exercise routines, and psycho-behavioral interventions, offering features such as customizable meal plans, exercise regimens, and motivational tools. The Placebo App, on the other hand, was designed only for completing forms related to diet adherence, exercise, and weight trends, without providing any feedback. Both groups followed a Mediterranean-based low-calorie diet, with a caloric deficit of 800 kcal from their total energy expenditure. The DTxO App triggered an estimated 41.57 minutes of daily usage, while the Placebo App group engaged the users only for 35.1 minutes of activity, mostly for physical exercise.

1b-ii) Level of human involvement in the METHODS section of the ABSTRACT

Clarify the level of human involvement in the abstract, e.g., use phrases like “fully automated” vs. “therapist/nurse/care provider/physician-assisted” (mention number and expertise of providers involved, if any). (Note: Only report in the abstract what the main paper is reporting. If this information is missing from the main body of text, consider adding it)

| | 1 | 2 | 3 | 4 | 5 | |
|---|---|---|---|---|---|---|
| subitem not at all important | ○ | ○ | ○ | ◉ | ○ | essential |

Cancella selezione

Does your paper address subitem 1b-ii?

Copy and paste relevant sections from the manuscript abstract (include quotes in quotation marks "like this" to indicate direct quotes from your manuscript), or elaborate on this item by providing additional information not in the ms, or briefly explain why the item is not applicable/relevant for your study

During the initial visit, participants underwent a comprehensive clinical history assessment and physical examination by a physician. Fasting blood sample was collected for the measurement of various biomarkers and anthropometric measurements were taken by a dietitian. A follow-up visit was scheduled 6 months after enrollment, during which the same measurements were reassessed, and a safety evaluation was performed.

1b-iii) Open vs. closed, web-based (self-assessment) vs. face-to-face assessments in the METHODS section of the ABSTRACT

Mention how participants were recruited (online vs. offline), e.g., from an open access website or from a clinic or a closed online user group (closed usergroup trial), and clarify if this was a purely web-based trial, or there were face-to-face components (as part of the intervention or for assessment). Clearly say if outcomes were self-assessed through questionnaires (as common in web-based trials). Note: In traditional offline trials, an open trial (open-label trial) is a type of clinical trial in which both the researchers and participants know which treatment is being administered. To avoid confusion, use “blinded” or “unblinded” to indicated the level of blinding instead of “open”, as “open” in web-based trials usually refers to “open access” (i.e. participants can self-enrol). (Note: Only report in the abstract what the main paper is reporting. If this information is missing from the main body of text, consider adding it)

| | 1 | 2 | 3 | 4 | 5 | |
|---|---|---|---|---|---|---|
| subitem not at all important | ○ | ○ | ○ | ○ | ○ | essential |

Does your paper address subitem 1b-iii?

Copy and paste relevant sections from the manuscript abstract (include quotes in quotation marks "like this" to indicate direct quotes from your manuscript), or elaborate on this item by providing additional information not in the ms, or briefly explain why the item is not applicable/relevant for your study

During the initial visit, participants underwent a comprehensive clinical history assessment and physical examination by a physician. Fasting blood sample was collected for the measurement of various biomarkers and anthropometric measurements were taken by a dietitian. A follow-up visit was scheduled 6 months after enrollment, during which the same measurements were reassessed, and a safety evaluation was performed.

1b-iv) RESULTS section in abstract must contain use data

Report number of participants enrolled/assessed in each group, the use/uptake of the intervention (e.g., attrition/adherence metrics, use over time, number of logins etc.), in addition to primary/secondary outcomes. (Note: Only report in the abstract what the main paper is reporting. If this information is missing from the main body of text, consider adding it)

| | 1 | 2 | 3 | 4 | 5 | |
|---|---|---|---|---|---|---|
| subitem not at all important | ○ | ○ | ○ | ○ | ○ | essential |

Does your paper address subitem 1b-iv?

Copy and paste relevant sections from the manuscript abstract (include quotes in quotation marks "like this" to indicate direct quotes from your manuscript), or elaborate on this item by providing additional information not in the ms, or briefly explain why the item is not applicable/relevant for your study

Overall, 207 (84.1%) completed the 6-month visit; both arms achieved a statistically significant absolute and percent loss in body weight after 6-month without significant between groups differences (univariable analysis with generalized linear models: P =.34 and P=.17, respectively).

1b-v) CONCLUSIONS/DISCUSSION in abstract for negative trials

Conclusions/Discussions in abstract for negative trials: Discuss the primary outcome - if the trial is negative (primary outcome not changed), and the intervention was not used, discuss whether negative results are attributable to lack of uptake and discuss reasons. (Note: Only report in the abstract what the main paper is reporting. If this information is missing from the main body of text, consider adding it)

| | 1 | 2 | 3 | 4 | 5 | |
|---|---|---|---|---|---|---|
| subitem not at all important | ○ | ○ | ○ | ○ | ◉ | essential |

Cancella selezione

Does your paper address subitem 1b-v?

Copy and paste relevant sections from the manuscript abstract (include quotes in quotation marks "like this" to indicate direct quotes from your manuscript), or elaborate on this item by providing additional information not in the ms, or briefly explain why the item is not applicable/relevant for your study

This trial demonstrated that while there were no significant overall differences in weight loss between the DTxO and placebo app groups, participants in the DTxO group who adhered to the intervention for at least 40% of the expected time showed significantly greater weight loss compared to the control group.

INTRODUCTION

2a) In INTRODUCTION: Scientific background and explanation of rationale

2a-i) Problem and the type of system/solution

Describe the problem and the type of system/solution that is object of the study: intended as stand-alone intervention vs. incorporated in broader health care program? Intended for a particular patient population? Goals of the intervention, e.g., being more cost-effective to other interventions, replace or complement other solutions? (Note: Details about the intervention are provided in “Methods” under 5)

| | 1 | 2 | 3 | 4 | 5 | |
|---|---|---|---|---|---|---|
| subitem not at all important | ○ | ○ | ○ | ○ | ◉ | essential |

Cancella selezione

Does your paper address subitem 2a-i? *

Copy and paste relevant sections from the manuscript (include quotes in quotation marks "like this" to indicate direct quotes from your manuscript), or elaborate on this item by providing additional information not in the ms, or briefly explain why the item is not applicable/relevant for your study

The treatment of people living with obesity continues to present significant challenges for healthcare professionals. For adults, guidelines recommend managing obesity as a chronic disease through multidisciplinary teams. These guidelines advocate for multicomponent lifestyle interventions—comprising diet, physical activity, and behavioral change strategies—for at least 6 to 12 months [5-7]. The dietary recommendations focus on developing personalized, balanced nutrition plans designed to reduce caloric intake, while fostering sustainable, long-term dietary habits that support weight loss and improve overall health. Physical activity should be customized to an individual's capabilities, preferences, and medical conditions to ensure both adherence and effectiveness, with the recommendation of at least 150 minutes of moderate-intensity aerobic exercise per week, complemented by strength training exercises twice a week [8]. Regarding behavioral strategies, mindfulness-based interventions to address emotional eating, stress, and other psychological barriers to weight management are recommended [8,9]. These interventions help individuals develop coping mechanisms, enhance self-regulation, and improve psychological well-being. Mindful eating practices are emphasized to increase awareness and self-regulation of eating behaviors, thoughts, feelings, and sensations that contribute to maladaptive eating patterns, thus supporting long-term weight maintenance [10]. Together, these interventions offer a comprehensive and holistic approach to obesity management, underscoring the importance of personalized care and continuous support. Recently, the use of gut hormone receptor agonists has emerged as an effective treatment option for obesity in combination with lifestyle interventions [11]. In addition, bariatric surgery may be considered for who have a BMI ≥35 kg/m², especially when nonsurgical interventions have been ineffective and comorbidities are present. However, despite these efforts, a common issue in obesity management—both in the short and long term—is patient adherence to lifestyle changes. Achieving the desired outcomes often requires multiple in-person sessions, which can be time-consuming, costly, and demanding for both patients and healthcare services [12].
In this context, alternative healthcare delivery models may be crucial for more effective obesity management. Given the evolving landscape of digital health, Digital Therapeutics (DTx) could play a pivotal role in improving and scaling healthcare interventions for obesity. DTx refers to "evidence-based therapeutic interventions using high-quality software programs to prevent, manage, or treat medical disorders or diseases," which must be certified by regulatory bodies as medical devices [13]. These interventions can provide scalable, accessible solutions for the management of obesity, representing an important step in the therapeutic algorithm. The algorithms underlying DTx may already be available in the literature or created de novo, incorporating different intervention modalities.

2a-ii) Scientific background, rationale: What is known about the (type of) system

Scientific background, rationale: What is known about the (type of) system that is the object of the study (be sure to discuss the use of similar systems for other conditions/diagnoses, if appropiate), motivation for the study, i.e. what are the reasons for and what is the context for this specific study, from which stakeholder viewpoint is the study performed, potential impact of findings [2]. Briefly justify the choice of the comparator.

| | 1 | 2 | 3 | 4 | 5 | |
|---|---|---|---|---|---|---|
| subitem not at all important | ○ | ○ | ○ | ○ | ◉ | essential |

Cancella selezione

Does your paper address subitem 2a-ii? *

Copy and paste relevant sections from the manuscript (include quotes in quotation marks "like this" to indicate direct quotes from your manuscript), or elaborate on this item by providing additional information not in the ms, or briefly explain why the item is not applicable/relevant for your study

While DTx have emerged as promising tools for treating obesity, there is a limited number of randomized controlled trials exploring their efficacy in this context. Among these studies, two incorporated psychological strategies targeting cognitive, emotional, and behavioral aspects of lifestyle changes [14] while others relied solely on behavioral strategies such as weight self-monitoring or time-restricted eating [15].
Most studies primarily used smartphones as the central intervention tool, with some integrating additional platforms like web-based systems or wearables to enhance the intervention. While many studies assessed clinical efficacy using a narrow range of metrics, fewer incorporated comprehensive, multidimensional assessments that address the full scope of factors influencing weight loss [16]. As a result, the outcomes regarding weight loss varied, highlighting the need for more robust and diverse evaluation methods.
A central focus of these studies was adherence to the intervention, with engagement rates typically exceeding 80%. This high level of adherence is crucial, as it directly correlates with the success of weight loss efforts [16]. A recent meta-analysis reviewing smartphone app-based interventions for weight loss reported a modest average weight loss of 2.03 kg after three months of app use. However, the significant heterogeneity between studies suggests that while these apps can be effective, their success is influenced by various factors, such as the integration of a multidimensional approach [ 17].

2b) In INTRODUCTION: Specific objectives or hypotheses

Does your paper address CONSORT subitem 2b? *

Copy and paste relevant sections from the manuscript (include quotes in quotation marks "like this" to indicate direct quotes from your manuscript), or elaborate on this item by providing additional information not in the ms, or briefly explain why the item is not applicable/relevant for your study

The aim of this prospective, multicenter, randomized, double-arm, single-blind, placebo-controlled trial was to assess the efficacy of an innovative DTx compared to a placebo application (app) in promoting weight loss over six months in people living with obesity. The study sought to improve adherence to treatment while providing continuous guidance and support throughout the therapeutic journey. A secondary objective was to evaluate risk factors and outcomes associated with body weight reduction in this patient population.

METHODS

3a) Description of trial design (such as parallel, factorial) including allocation ratio

Does your paper address CONSORT subitem 3a? *

Copy and paste relevant sections from the manuscript (include quotes in quotation marks "like this" to indicate direct quotes from your manuscript), or elaborate on this item by providing additional information not in the ms, or briefly explain why the item is not applicable/relevant for your study

This prospective, multicenter, randomized, double-arm, single-blind, placebo-controlled trial aimed to evaluate the effectiveness of a novel digital therapy (DTxO) in improving the clinical outcomes of people living with obesity compared to Placebo App (control group). DTxO encompasses a comprehensive set of interventions, including a personalized dietary plan, a tailored exercise program, cognitive-behavioral support, reminders for medication adherence, and online consultations with healthcare professionals. The study recruited adult people living with obesity seeking weight-loss treatment at the participating centers.

3b) Important changes to methods after trial commencement (such as eligibility criteria), with reasons

Does your paper address CONSORT subitem 3b? *

Copy and paste relevant sections from the manuscript (include quotes in quotation marks "like this" to indicate direct quotes from your manuscript), or elaborate on this item by providing additional information not in the ms, or briefly explain why the item is not applicable/relevant for your study

The study design and methods were detailed in a previously published protocol paper [18] and no changes were made to the protocol during the study. The study was prospectively registered to clinicaltrials.gov, identifier, NCT05394779 on 2022-08-23. Below, we provide a summary of the key features of the population and intervention.

3b-i) Bug fixes, Downtimes, Content Changes

Bug fixes, Downtimes, Content Changes: ehealth systems are often dynamic systems. A description of changes to methods therefore also includes important changes made on the intervention or comparator during the trial (e.g., major bug fixes or changes in the functionality or content) (5-iii) and other "unexpected events" that may have influenced study design such as staff changes, system failures/downtimes, etc. [2].

| | 1 | 2 | 3 | 4 | 5 | |
|---|---|---|---|---|---|---|
| subitem not at all important | ○ | ○ | ◉ | ○ | ○ | essential |

Cancella selezione

Does your paper address subitem 3b-i?

Copy and paste relevant sections from the manuscript (include quotes in quotation marks "like this" to indicate direct quotes from your manuscript), or elaborate on this item by providing additional information not in the ms, or briefly explain why the item is not applicable/relevant for your study

For the duration of the trial, we ensured that the DTxO and Placebo apps were maintained with minimal disruptions. Any technical issues, such as bugs or downtimes, were promptly addressed by our technical support team to ensure smooth usage for the participants.

4a) Eligibility criteria for participants

Does your paper address CONSORT subitem 4a? *

Copy and paste relevant sections from the manuscript (include quotes in quotation marks "like this" to indicate direct quotes from your manuscript), or elaborate on this item by providing additional information not in the ms, or briefly explain why the item is not applicable/relevant for your study

Enrolled participants were between the ages of 18 and 65, with a body mass index (BMI) of 30-45 kg/m², and were proficient in using mobile apps, as the digital therapy was app-based. All instructions and guidance were provided in Italian, so participants were required to be fluent in the language.
Exclusion criteria included recent cardiovascular events, severe heart failure, ischemic attack, or stroke within the last 6 months prior to the planned randomization date; chronic kidney failure; type 1 diabetes; previous malignancy within the past 5 years; visual impairments (e.g., complete or nearly complete vision loss, glaucoma); secondary obesity related to endocrinopathies, genetic syndromes, or hypothalamic lesions; advanced obesity disease (stage 4 on the Edmonton Obesity Staging), [19]; uncontrolled psychiatric disorders; active eating disorders or a history of bulimia or anorexia nervosa; active substance abuse; history of bariatric surgery within the previous 2 years or plans for surgery during the study (e.g., sleeve gastrectomy, gastric banding, gastric bypass); changes in pharmacological treatments affecting appetite or metabolism in the last 3-6 months; involvement in other weight-loss programs or trials; referred pain in lower limb joints (hip, knee, ankle) with a Numeric Rating Scale (NRS) score ≥ 5 [20]; or weight loss ≥10% in the 6 months prior to randomization.

Screening during the baseline visit ensured that all participants met the inclusion criteria and did not fall under any of the exclusion categories.

4a-i) Computer / Internet literacy

Computer / Internet literacy is often an implicit “de facto” eligibility criterion - this should be explicitly clarified.

| | 1 | 2 | 3 | 4 | 5 | |
|---|---|---|---|---|---|---|
| subitem not at all important | ○ | ○ | ○ | ○ | ◉ | essential |

Cancella selezione

Does your paper address subitem 4a-i?

Copy and paste relevant sections from the manuscript (include quotes in quotation marks "like this" to indicate direct quotes from your manuscript), or elaborate on this item by providing additional information not in the ms, or briefly explain why the item is not applicable/relevant for your study

Enrolled participants were between the ages of 18 and 65 were proficient in using mobile apps, as the digital therapy was app-based. All instructions and guidance were provided in Italian, so participants were required to be fluent in the language.

4a-ii) Open vs. closed, web-based vs. face-to-face assessments:

Open vs. closed, web-based vs. face-to-face assessments: Mention how participants were recruited (online vs. offline), e.g., from an open access website or from a clinic, and clarify if this was a purely web-based trial, or there were face-to-face components (as part of the intervention or for assessment), i.e., to what degree got the study team to know the participant. In online-only trials, clarify if participants were quasi-anonymous and whether having multiple identities was possible or whether technical or logistical measures (e.g., cookies, email confirmation, phone calls) were used to detect/prevent these.

| | 1 | 2 | 3 | 4 | 5 | |
|---|---|---|---|---|---|---|
| subitem not at all important | ○ | ○ | ○ | ○ | ● | essential |

Cancella selezione

Does your paper address subitem 4a-ii? *

Copy and paste relevant sections from the manuscript (include quotes in quotation marks "like this" to indicate direct quotes from your manuscript), or elaborate on this item by providing additional information not in the ms, or briefly explain why the item is not applicable/relevant for your study

During the initial visit, the participant underwent a clinical history assessment and a physical examination by a physician (either an endocrinologist or a nutrition specialist). Demographic data were collected, including date of birth, age, gender, education, marital status, occupation, family composition, and ethnicity. Additionally, the physician gathered information on the participant's personal and family medical history, including cardiovascular disease, diabetes, and other chronic conditions, as well as current and previous pharmacological treatments, menopausal status, and lifestyle habits such as smoking and structured physical activity. Blood pressure was measured according to international guidelines from the European Society of Hypertension (ESH) and the European Society of Cardiology (ESC) for both clinical and out-of-office blood pressure measurements [21].
A fasting blood sample was drawn between 08:30 and 09:00 AM for the measurement of blood glucose, insulin, triglycerides, total cholesterol, low-density lipoprotein (LDL) cholesterol, high-density lipoprotein (HDL) cholesterol, alanine transaminase (ALT), aspartate transaminase (AST), gamma-glutamyl transferase (GGT), thyroid-stimulating hormone (TSH), and free thyroxine (FT4). The Insulin Resistance index and glomerular filtration rate (GFR) were calculated using validated formulas [22,23].
Finally, a registered dietitian performed anthropometric measurements according to international guidelines [24]. Weight was measured using an electronic scale with 100 g accuracy (Seca 700, Seca Corporation, Hamburg, Germany), and height was measured using a vertical stadiometer with 0.1 cm accuracy. Waist circumference (WC) was measured to the nearest 0.5 cm using a non-elastic tape placed at the midpoint between the last rib and the iliac crest.

4a-iii) Information giving during recruitment

Information given during recruitment. Specify how participants were briefed for recruitment and in the informed consent procedures (e.g., publish the informed consent documentation as appendix, see also item X26), as this information may have an effect on user self-selection, user expectation and may also bias results.

| | 1 | 2 | 3 | 4 | 5 | |
|---|---|---|---|---|---|---|
| subitem not at all important | ○ | ○ | ○ | ○ | ◉ | essential |

Cancella selezione

Does your paper address subitem 4a-iii?

Copy and paste relevant sections from the manuscript (include quotes in quotation marks "like this" to indicate direct quotes from your manuscript), or elaborate on this item by providing additional information not in the ms, or briefly explain why the item is not applicable/relevant for your study

Informed consent descriptions: Informed consent document outlined the clinical trial's purpose, key details, and the rights and responsibilities of participants, ensuring they could make an informed, voluntary decision. Participants were encouraged to ask questions and consult with their doctor or trusted individuals. They were given time to review and discuss the document before deciding, and if they chose not to participate, they still received the best possible care. Informed consent was obtained in writing from each participant prior to their involvement.

4b) Settings and locations where the data were collected

Does your paper address CONSORT subitem 4b? *

Copy and paste relevant sections from the manuscript (include quotes in quotation marks "like this" to indicate direct quotes from your manuscript), or elaborate on this item by providing additional information not in the ms, or briefly explain why the item is not applicable/relevant for your study

The trial was conducted at two Italian obesity care centers: IRCCS Istituto Auxologico Italiano (Center 1) in the North and Policlinico di Bari, Giovanni XXIII Hospital (Center 2) in the South, ensuring geographical and lifestyle diversity among the enrolled participants. Enrolment took place during a visit with an endocrinologist or nutrition specialist, with informed consent obtained before any screening procedures were conducted.

4b-i) Report if outcomes were (self-)assessed through online questionnaires

Clearly report if outcomes were (self-)assessed through online questionnaires (as common in web-based trials) or otherwise.

| | 1 | 2 | 3 | 4 | 5 | |
|---|---|---|---|---|---|---|
| subitem not at all important | ○ | ○ | ○ | ○ | ◉ | essential |

Cancella selezione

Does your paper address subitem 4b-i? *

Copy and paste relevant sections from the manuscript (include quotes in quotation marks "like this" to indicate direct quotes from your manuscript), or elaborate on this item by providing additional information not in the ms, or briefly explain why the item is not applicable/relevant for your study

Follow-up Visit
A face-to-face follow-up visit was scheduled for 6 months after enrollment. During this visit, the same parameters collected at the baseline visit were reassessed. Additionally, concomitant medications were recorded, and a safety assessment was performed.

4b-ii) Report how institutional affiliations are displayed

Report how institutional affiliations are displayed to potential participants [on ehealth media], as affiliations with prestigious hospitals or universities may affect volunteer rates, use, and reactions with regards to an intervention.(Not a required item – describe only if this may bias results)

| | 1 | 2 | 3 | 4 | 5 | |
|---|---|---|---|---|---|---|
| subitem not at all important | ○ | ○ | ○ | ◉ | ○ | essential |

Cancella selezione

Does your paper address subitem 4b-ii?

Copy and paste relevant sections from the manuscript (include quotes in quotation marks "like this" to indicate direct quotes from your manuscript), or elaborate on this item by providing additional information not in the ms, or briefly explain why the item is not applicable/relevant for your study

The study recruited adult people living with obesity seeking weight-loss treatment at the participating centers.

5) The interventions for each group with sufficient details to allow replication, including how and when they were actually administered

5-i) Mention names, credential, affiliations of the developers, sponsors, and owners

Mention names, credential, affiliations of the developers, sponsors, and owners [6] (if authors/evaluators are owners or developer of the software, this needs to be declared in a “Conflict of interest” section or mentioned elsewhere in the manuscript).

| | 1 | 2 | 3 | 4 | 5 | |
|---|---|---|---|---|---|---|
| subitem not at all important | ○ | ○ | ○ | ○ | ◉ | essential |

Cancella selezione

Does your paper address subitem 5-i?

Copy and paste relevant sections from the manuscript (include quotes in quotation marks "like this" to indicate direct quotes from your manuscript), or elaborate on this item by providing additional information not in the ms, or briefly explain why the item is not applicable/relevant for your study

Digital Therapeutics for Obesity: intervention arm
The study intervention, DTxO, is an investigational digital therapy developed by Advice Pharma Group S.r.l. for people living with obesity, designed to support weight loss, maintenance, and overall health improvement. Classified as a Class IIa Medical Device, DTxO is a mobile application that integrates non-pharmacological approaches. The app aims to enhance patient engagement, self-monitoring, and adherence to dietary, exercise, and behavioral programs. It includes tools for tracking diet, exercise, psychological status, and non-vital parameters, providing customizable charts for healthcare professionals.
The DTxO app offers a personalized diet and exercise plan, cognitive-behavioral support, reminders, drug intake tracking, and online communication with healthcare professionals. It also incorporates motivational tools, such as trophies, to further engage patients. The entire therapeutic algorithm has been designed based on guidelines advocating for a multidisciplinary approach, incorporating various healthcare professionals, including nutritionists, endocrinologists, psychologists, and dietitians [6-8]. Patients began using the app after completing psychological questionnaires and inputting food preferences during their baseline visit.
The control arm followed a standardized paper-based diet, as outlined in the Dietary intervention paragraph (referred to as the traditional diet), along with a paper exercise plan. Participants in the control group used the Placebo App solely to complete forms related to diet adherence, exercise, and weight trends, without any personalization, alerts, reminders, or online support. Additionally, participants in the Placebo App group did not have access to the psycho-behavioral program.
The Placebo App was designed by the investigative team to provide a comparable experience to the DTxO group, simplifying data collection and minimizing potential errors.

5-ii) Describe the history/development process

Describe the history/development process of the application and previous formative evaluations (e.g., focus groups, usability testing), as these will have an impact on adoption/use rates and help with interpreting results.

| | 1 | 2 | 3 | 4 | 5 | |
|---|---|---|---|---|---|---|
| subitem not at all important | ○ | ○ | ◉ | ○ | ○ | essential |

Cancella selezione

Does your paper address subitem 5-ii?

Copy and paste relevant sections from the manuscript (include quotes in quotation marks "like this" to indicate direct quotes from your manuscript), or elaborate on this item by providing additional information not in the ms, or briefly explain why the item is not applicable/relevant for your study

We previously evaluated the usability of the app in patients with obesity who participated in an experimental LS-WLp program. In addition, the usability of the Medical Panel was assessed through simulated use during working group sessions and feedback from expert users. The suggestions collected were used to improve the DTxO platform. These data are not presented in the current manuscript.

5-iii) Revisions and updating

Revisions and updating. Clearly mention the date and/or version number of the application/intervention (and comparator, if applicable) evaluated, or describe whether the intervention underwent major changes during the evaluation process, or whether the development and/or content was "frozen" during the trial. Describe dynamic components such as news feeds or changing content which may have an impact on the replicability of the intervention (for unexpected events see item 3b).

| | 1 | 2 | 3 | 4 | 5 | |
|---|---|---|---|---|---|---|
| subitem not at all important | ○ | ○ | ○ | ○ | ◉ | essential |

Cancella selezione

Does your paper address subitem 5-iii?

Copy and paste relevant sections from the manuscript (include quotes in quotation marks "like this" to indicate direct quotes from your manuscript), or elaborate on this item by providing additional information not in the ms, or briefly explain why the item is not applicable/relevant for your study

No revisions were necessary

5-iv) Quality assurance methods

Provide information on quality assurance methods to ensure accuracy and quality of information provided [1], if applicable.

| | 1 | 2 | 3 | 4 | 5 | |
|---|---|---|---|---|---|---|
| subitem not at all important | ○ | ○ | ◉ | ○ | ○ | essential |

Cancella selezione

Does your paper address subitem 5-iv?

Copy and paste relevant sections from the manuscript (include quotes in quotation marks "like this" to indicate direct quotes from your manuscript), or elaborate on this item by providing additional information not in the ms, or briefly explain why the item is not applicable/relevant for your study

The clinical outcomes of the study (nutritional status and laboratory tests) are based on robust data collected by trained personnel during the baseline visit at enrollment and the follow-up visit at 6 months. Laboratory parameters were measured at the recruitment centers.

5-v) Ensure replicability by publishing the source code, and/or providing screenshots/screen-capture video, and/or providing flowcharts of the algorithms used

Ensure replicability by publishing the source code, and/or providing screenshots/screen-capture video, and/or providing flowcharts of the algorithms used. Replicability (i.e., other researchers should in principle be able to replicate the study) is a hallmark of scientific reporting.

| | 1 | 2 | 3 | 4 | 5 | |
|---|---|---|---|---|---|---|
| subitem not at all important | ○ | ○ | ○ | ○ | ◉ | essential |

Cancella selezione

Does your paper address subitem 5-v?

Copy and paste relevant sections from the manuscript (include quotes in quotation marks "like this" to indicate direct quotes from your manuscript), or elaborate on this item by providing additional information not in the ms, or briefly explain why the item is not applicable/relevant for your study

Main DTxO's features

Food Section: upon the first login, patients were able to personalize their assigned dietary program by selecting the number of meals and food preferences from a list of options for each food category (e.g., choosing or excluding pasta, barley, rice, or spelt among cereals). Patients could opt to create their own personalized menu or follow the general diet structure while adhering to prescribed portions and food frequencies. The app also allowed users to organize meals through a shopping list and provided access to supporting recipes. Additionally, the app included educational modules on food, hydration strategies, and uncommon foods (e.g., quinoa or legume derivatives) to encourage variety in food choices.
Physical Activity Section: in this section, each patient followed a prescribed exercise program, including details on the recommended type, duration, frequency, and intensity of activity. After each exercise session, patients rated their perceived fatigue and any pain using a numerical rating scale [12]: 0 = no effort/pain ("too soft"), 3 = moderate effort/pain ("tolerable"), and 10 = maximum effort/pain ("too hard"). Based on this feedback, the app automatically adjusted the type, duration, frequency, and intensity of subsequent exercises. The physical activity carried out was self-reported. Exercise sequences were accompanied by short explanatory videos and educational content tailored to each activity.
Psycho-Behavioural Section: it provided educational content through brief text modules. Patients were offered two exercises per week for each of the five main psycho-behavioral areas. These exercises could be completed at any time during the week and were delivered via text or audio, with the audio recordings guided by a psychologist.

5-vi) Digital preservation

Digital preservation: Provide the URL of the application, but as the intervention is likely to change or disappear over the course of the years; also make sure the intervention is archived (Internet Archive, webcitation.org, and/or publishing the source code or screenshots/videos alongside the article). As pages behind login screens cannot be archived, consider creating demo pages which are accessible without login.

| | 1 | 2 | 3 | 4 | 5 | |
|---|---|---|---|---|---|---|
| subitem not at all important | ○ | ○ | ◉ | ○ | ○ | essential |

Cancella selezione

Does your paper address subitem 5-vi?

Copy and paste relevant sections from the manuscript (include quotes in quotation marks "like this" to indicate direct quotes from your manuscript), or elaborate on this item by providing additional information not in the ms, or briefly explain why the item is not applicable/relevant for your study

At this time, the URL is not available, as access to the platform is currently restricted. The platform is classified as a Class IIa Medical Device, and the CE marking process is currently underway. Public access will be granted upon completion of the evaluation and certification procedures.

5-vii) Access

Access: Describe how participants accessed the application, in what setting/context, if they had to pay (or were paid) or not, whether they had to be a member of specific group. If known, describe how participants obtained "access to the platform and Internet" [1]. To ensure access for editors/reviewers/readers, consider to provide a "backdoor" login account or demo mode for reviewers/readers to explore the application (also important for archiving purposes, see vi).

| | 1 | 2 | 3 | 4 | 5 | |
|---|---|---|---|---|---|---|
| subitem not at all important | ○ | ○ | ◉ | ○ | ○ | essential |

Cancella selezione

Does your paper address subitem 5-vii? *

Copy and paste relevant sections from the manuscript (include quotes in quotation marks "like this" to indicate direct quotes from your manuscript), or elaborate on this item by providing additional information not in the ms, or briefly explain why the item is not applicable/relevant for your study

Once available, access to the application will be granted following a specialist medical visit, during which the digital therapeutic intervention will be prescribed and activated. Access to the platform will be subject to a fee; however, support from the Italian National Health System is considered desirable to ensure broader accessibility.

5-viii) Mode of delivery, features/functionalities/components of the intervention and comparator, and the theoretical framework

Describe mode of delivery, features/functionalities/components of the intervention and comparator, and the theoretical framework [6] used to design them (instructional strategy [1], behaviour change techniques, persuasive features, etc., see e.g., [7, 8] for terminology). This includes an in-depth description of the content (including where it is coming from and who developed it) [1]," whether [and how] it is tailored to individual circumstances and allows users to track their progress and receive feedback" [6]. This also includes a description of communication delivery channels and – if computer-mediated communication is a component – whether communication was synchronous or asynchronous [6]. It also includes information on presentation strategies [1], including page design principles, average amount of text on pages, presence of hyperlinks to other resources, etc. [1].

| | 1 | 2 | 3 | 4 | 5 | |
|---|---|---|---|---|---|---|
| subitem not at all important | ○ | ○ | ○ | ◉ | ○ | essential |

Cancella selezione

Does your paper address subitem 5-viii? *

Copy and paste relevant sections from the manuscript (include quotes in quotation marks "like this" to indicate direct quotes from your manuscript), or elaborate on this item by providing additional information not in the ms, or briefly explain why the item is not applicable/relevant for your study

According to the Italian Guidelines for Dietary Obesity Management [26], the dietary intervention consisted of a personalized Mediterranean-based low-calorie diet, tailored to each participant’s gender, age, physical activity level, food preferences, and eating habits. The diet was prescribed by a nutritional physician during the baseline visit, with a daily caloric intake (kcal/day) calculated based on a standardized method. This method involved a fixed caloric deficit of 800 kcal relative to the participant's total energy expenditure, which was estimated using the Mifflin-St Jeor formula [27] for resting energy expenditure, and the level of physical activity, assessed using the short version of the International Physical Activity Questionnaire (IPAQ) [28].
The dietary program included standardized macronutrient proportions, with 45-50% of total calories from carbohydrates and 30-35% from fats, all derived from typical Mediterranean foods (e.g., nuts, extra virgin olive oil, fish, red wine, whole grains, legumes). Participants were given the option to follow a meal plan consisting of three daily meals (breakfast, lunch, and dinner) or five smaller meals (breakfast, mid-morning snack, lunch, afternoon snack, and dinner), depending on their personal preferences and habitual eating patterns.
Physical Activity Intervention
The physical activity intervention was tailored to each individual's fitness level, based on the results of the IPAQ questionnaire [28], which was self-administered by the patient at baseline.
Psycho-Behavioural intervention
The psycho-behavioral intervention was offered exclusively to the intervention arm, aiming to increase awareness of behaviors and habits related to obesity. It was grounded in the theoretical framework of mindfulness, which refers to the learned ability to remain present in the moment with an open and non-judgmental attitude. Research has shown that mindfulness practice increases awareness of habitual patterns of thoughts, emotions, and behaviors, allowing for more adaptive responses. Clinically, this translates into improved emotional regulation, greater self-compassion, and enhanced self-control—factors essential in addressing dysfunctional eating behaviors [29].
Specifically, in DTxO, mindful eating was implemented as an approach that enhances awareness of both the psychological and physiological aspects of eating. A recent review explored the effectiveness of integrating mindful eating into weight management programs. While the results were sometimes modest compared to traditional methods, mindful eating has been shown to support long-term behavioral change. It increases awareness of hunger and satiety cues, reduces cravings and emotional eating, and fosters greater self-compassion [29,30]
It included multimedia and educational content, self-assessments, and dynamic exercises across five key areas to support control and containment efforts [30]. These areas were: a) commitment and motivation, b) openness and availability, c) awareness and mindfulness, d) emotional eating, and e) self-efficacy. For each of these five areas, patients could complete two exercises at any time during the day or week, allowing for flexibility and personalization.

5-ix) Describe use parameters

Describe use parameters (e.g., intended “doses” and optimal timing for use). Clarify what instructions or recommendations were given to the user, e.g., regarding timing, frequency, heaviness of use, if any, or was the intervention used ad libitum.

| | 1 | 2 | 3 | 4 | 5 | |
|---|---|---|---|---|---|---|
| subitem not at all important | ○ | ○ | ○ | ○ | ◉ | essential |

Cancella selezione

Does your paper address subitem 5-ix?

Copy and paste relevant sections from the manuscript (include quotes in quotation marks "like this" to indicate direct quotes from your manuscript), or elaborate on this item by providing additional information not in the ms, or briefly explain why the item is not applicable/relevant for your study

The proposed dosing times for DTxO were as follows: -Food Section: 10 minutes per week, estimated time for composing the weekly menu and reviewing the proposed content; -Weight Diary Section: 2 minutes every two weeks, estimated time for recording weight, including the time for measurement on the scale;-Physical Activity Section: 35 minutes per day, estimated time needed to complete the prescribed exercises; Psychological Section: 5 minutes per day, estimated time for performing at least one mindfulness exercise from those offered.
When converted to daily usage, the digital device doses were as follows: Food Section: 1.43 minutes/day; Weight Diary Section: 0.14 minutes/day; Physical Activity Section: 35 minutes/day; Psychological Section: 5 minutes/day
The total estimated daily adherence for 100% use of DTxO corresponded to 41.57 minutes/day.

5-x) Clarify the level of human involvement

Clarify the level of human involvement (care providers or health professionals, also technical assistance) in the e-intervention or as co-intervention (detail number and expertise of professionals involved, if any, as well as “type of assistance offered, the timing and frequency of the support, how it is initiated, and the medium by which the assistance is delivered”. It may be necessary to distinguish between the level of human involvement required for the trial, and the level of human involvement required for a routine application outside of a RCT setting (discuss under item 21 – generalizability).

| | 1 | 2 | 3 | 4 | 5 | |
|---|---|---|---|---|---|---|
| subitem not at all important | ○ | ○ | ○ | ◉ | ○ | essential |

Cancella selezione

Does your paper address subitem 5-x?

Copy and paste relevant sections from the manuscript (include quotes in quotation marks "like this" to indicate direct quotes from your manuscript), or elaborate on this item by providing additional information not in the ms, or briefly explain why the item is not applicable/relevant for your study

The level of human involvement in the e-intervention varied depending on the study phase. During the trial, participants received structured support from a multidisciplinary team, including dietitians, psychologists, and physicians specialized in obesity treatment.

The type of assistance provided included an initial in-person medical visit to assess eligibility and prescribe the digital therapeutic intervention (DTxO), followed by remote support as needed. Technical assistance with app use was available on request via email or telephone.
In a routine clinical setting (outside of the RCT), the level of human involvement is expected to be lower. The app is designed for autonomous use, with most of the content delivered digitally. Assistance may also include customer or technical support via digital channels.

5-xi) Report any prompts/reminders used

Report any prompts/reminders used: Clarify if there were prompts (letters, emails, phone calls, SMS) to use the application, what triggered them, frequency etc. It may be necessary to distinguish between the level of prompts/reminders required for the trial, and the level of prompts/reminders for a routine application outside of a RCT setting (discuss under item 21 – generalizability).

| | 1 | 2 | 3 | 4 | 5 | |
|---|---|---|---|---|---|---|
| subitem not at all important | ○ | ○ | ○ | ◉ | ○ | essential |

Cancella selezione

Does your paper address subitem 5-xi? *

Copy and paste relevant sections from the manuscript (include quotes in quotation marks "like this" to indicate direct quotes from your manuscript), or elaborate on this item by providing additional information not in the ms, or briefly explain why the item is not applicable/relevant for your study

During the trial, participants received automated in-app notifications (reminders) to promote regular engagement with the application and adherence to the proposed activities. In addition to reminders, the app included a system of virtual rewards (trophies) to reinforce motivation and recognize user achievements throughout the program. No SMS, phone calls, or letters were used.

5-xii) Describe any co-interventions (incl. training/support)

Describe any co-interventions (incl. training/support): Clearly state any interventions that are provided in addition to the targeted eHealth intervention, as ehealth intervention may not be designed as stand-alone intervention. This includes training sessions and support [1]. It may be necessary to distinguish between the level of training required for the trial, and the level of training for a routine application outside of a RCT setting (discuss under item 21 – generalizability.

| | 1 | 2 | 3 | 4 | 5 | |
|---|---|---|---|---|---|---|
| subitem not at all important | ○ | ○ | ○ | ◉ | ○ | essential |

Cancella selezione

Does your paper address subitem 5-xii? *

Copy and paste relevant sections from the manuscript (include quotes in quotation marks "like this" to indicate direct quotes from your manuscript), or elaborate on this item by providing additional information not in the ms, or briefly explain why the item is not applicable/relevant for your study

In addition to the eHealth intervention (DTxO), participants in both groups received an initial in-person medical visit conducted by a specialist. This visit served as both a clinical evaluation and an opportunity to introduce and explain the use of the digital therapeutic or placebo app. No additional structured training sessions were provided during the trial.

6a) Completely defined pre-specified primary and secondary outcome measures, including how and when they were assessed

Does your paper address CONSORT subitem 6a? *

Copy and paste relevant sections from the manuscript (include quotes in quotation marks "like this" to indicate direct quotes from your manuscript), or elaborate on this item by providing additional information not in the ms, or briefly explain why the item is not applicable/relevant for your study

The aim of this prospective, multicenter, randomized, double-arm, single-blind, placebo-controlled trial was to assess the efficacy of an innovative DTx compared to a placebo application (app) in promoting weight loss over six months in people living with obesity. The study sought to improve adherence to treatment while providing continuous guidance and support throughout the therapeutic journey. A secondary objective was to evaluate risk factors and outcomes associated with body weight reduction in this patient population.

6a-i) Online questionnaires: describe if they were validated for online use and apply CHERRIES items to describe how the questionnaires were designed/deployed

If outcomes were obtained through online questionnaires, describe if they were validated for online use and apply CHERRIES items to describe how the questionnaires were designed/deployed [9].

| | 1 | 2 | 3 | 4 | 5 | |
|---|---|---|---|---|---|---|
| subitem not at all important | ○ | ○ | ○ | ○ | ○ | essential |

Does your paper address subitem 6a-i?

Copy and paste relevant sections from manuscript text

Outcomes were not obtained through online questionnaires in this study. All clinical and behavioral data, including anthropometric measurements, biochemical parameters, and psychological/behavioral assessments, were collected in person by trained healthcare professionals during the baseline and 6-month follow-up visits.

However, some self-monitoring activities and progress tracking were integrated into the DTxO app, including self-assessment exercises related to emotional eating and motivation. These digital tools were not intended for research data collection purposes and were not used as outcome measures. Therefore, no formal online questionnaires were deployed, and CHERRIES criteria were not applicable in this context.

6a-ii) Describe whether and how “use” (including intensity of use/dosage) was defined/measured/monitored

Describe whether and how “use” (including intensity of use/dosage) was defined/measured/monitored (logins, logfile analysis, etc.). Use/adoption metrics are important process outcomes that should be reported in any ehealth trial.

| | 1 | 2 | 3 | 4 | 5 | |
|---|---|---|---|---|---|---|
| subitem not at all important | ○ | ○ | ○ | ● | ○ | essential |

Cancella selezione

Does your paper address subitem 6a-ii?

Copy and paste relevant sections from manuscript text

In this study, "use" of the digital therapeutic application (DTxO) was measured and monitored through in-app tracking features, which recorded key metrics such as logins, session duration, frequency of app usage, and completion of specific activities. The intensity of use was defined by the number of times a participant logged into the app, the duration of each session, and the completion rate of assigned tasks within the app.

Logfile analysis was used to capture data related to participant engagement, including how often users interacted with specific features of the app (e.g., mindfulness exercises, self-assessments). This allowed for a detailed monitoring of app usage over the course of the trial.

These usage metrics were also used to assess participant adherence to the digital intervention, which is a critical process outcome in eHealth trials.

6a-iii) Describe whether, how, and when qualitative feedback from participants was obtained

Describe whether, how, and when qualitative feedback from participants was obtained (e.g., through emails, feedback forms, interviews, focus groups).

| | 1 | 2 | 3 | 4 | 5 | |
|---|---|---|---|---|---|---|
| subitem not at all important | ○ | ○ | ○ | ○ | ● | essential |

Cancella selezione

Does your paper address subitem 6a-iii?

Copy and paste relevant sections from manuscript text

In this study, qualitative feedback from participants was not systematically obtained. No interviews, focus groups, or feedback forms were conducted to gather participant insights or experiences.

6b) Any changes to trial outcomes after the trial commenced, with reasons

Does your paper address CONSORT subitem 6b? *

Copy and paste relevant sections from the manuscript (include quotes in quotation marks "like this" to indicate direct quotes from your manuscript), or elaborate on this item by providing additional information not in the ms, or briefly explain why the item is not applicable/relevant for your study

No changes were made to the trial outcomes after the trial commenced. The outcomes outlined in the study protocol were adhered to throughout the trial, and no modifications were necessary.

7a) How sample size was determined

NPT: When applicable, details of whether and how the clustering by care provides or centers was addressed

7a-i) Describe whether and how expected attrition was taken into account when calculating the sample size

Describe whether and how expected attrition was taken into account when calculating the sample size.

| | 1 | 2 | 3 | 4 | 5 | |
|---|---|---|---|---|---|---|
| subitem not at all important | ○ | ○ | ○ | ○ | ◉ | essential |

Cancella selezione

Does your paper address subitem 7a-i?

Copy and paste relevant sections from manuscript title (include quotes in quotation marks "like this" to indicate direct quotes from your manuscript), or elaborate on this item by providing additional information not in the ms, or briefly explain why the item is not applicable/relevant for your study

The sample size was computed considering the primary endpoint, namely the change in absolute body weight (kg) at 6 months from baseline. A total sample size of 172 patients (86 per group) would allow, with 80% power and a two-sided type I error of 0.05, to detect a 1.5 kg difference between the groups in weight change at 6 months, assuming a standard deviation (SD) of 3.5 kg. The goal of randomizing 246 patients was set to account for a drop-out rate of 30% [25]. The sample size was computed using the “proc power” procedure of SAS software v.9.4 (Cary, NC, USA).
Since in earlier studies, the efficacy of several pharmacotherapies for adults with obesity was measured as a mean difference of less than 2 kg as compared to placebo, in this study, 1.5 kg more weight loss from baseline in the active group (DTXO group) – i.e., without a pharmacological intervention with potential related adverse events – compared to the Control Group, was considered clinically important.

7b) When applicable, explanation of any interim analyses and stopping guidelines

Does your paper address CONSORT subitem 7b? *

Copy and paste relevant sections from the manuscript (include quotes in quotation marks "like this" to indicate direct quotes from your manuscript), or elaborate on this item by providing additional information not in the ms, or briefly explain why the item is not applicable/relevant for your study

No interim analyses or stopping guidelines were applied in this study. The trial proceeded according to the original protocol without any planned or unplanned interim assessments.

8a) Method used to generate the random allocation sequence

NPT: When applicable, how care providers were allocated to each trial group

Does your paper address CONSORT subitem 8a? *

Copy and paste relevant sections from the manuscript (include quotes in quotation marks "like this" to indicate direct quotes from your manuscript), or elaborate on this item by providing additional information not in the ms, or briefly explain why the item is not applicable/relevant for your study

To maintain an overall balance between groups, block randomization was performed (using random block sizes of 8 patients) using the "proc plan" procedure in SAS ( Institute, Cary, NC, release 9.4).

8b) Type of randomisation; details of any restriction (such as blocking and block size)

Does your paper address CONSORT subitem 8b? *

Copy and paste relevant sections from the manuscript (include quotes in quotation marks "like this" to indicate direct quotes from your manuscript), or elaborate on this item by providing additional information not in the ms, or briefly explain why the item is not applicable/relevant for your study

Patients were randomly assigned to the DTxO App or the Placebo App on a 1:1 basis according to a pre-defined, centralized randomization list.

9) Mechanism used to implement the random allocation sequence (such as sequentially numbered containers), describing any steps taken to conceal the sequence until interventions were assigned

Does your paper address CONSORT subitem 9? *

Copy and paste relevant sections from the manuscript (include quotes in quotation marks "like this" to indicate direct quotes from your manuscript), or elaborate on this item by providing additional information not in the ms, or briefly explain why the item is not applicable/relevant for your study

Block randomization was performed (using random block sizes of 8 patients) using the "proc plan" procedure in SAS ( Institute, Cary, NC, release 9.4).

10) Who generated the random allocation sequence, who enrolled participants, and who assigned participants to interventions

Does your paper address CONSORT subitem 10? *

Copy and paste relevant sections from the manuscript (include quotes in quotation marks "like this" to indicate direct quotes from your manuscript), or elaborate on this item by providing additional information not in the ms, or briefly explain why the item is not applicable/relevant for your study

The random allocation sequence was generated by a statistician who was independent of the recruitment and intervention processes.

11a) If done, who was blinded after assignment to interventions (for example, participants, care providers, those assessing outcomes) and how

NPT: Whether or not administering co-interventions were blinded to group assignment

11a-i) Specify who was blinded, and who wasn't

Specify who was blinded, and who wasn't. Usually, in web-based trials it is not possible to blind the participants [1, 3] (this should be clearly acknowledged), but it may be possible to blind outcome assessors, those doing data analysis or those administering co-interventions (if any).

| | 1 | 2 | 3 | 4 | 5 | |
|---|---|---|---|---|---|---|
| subitem not at all important | ○ | ○ | ○ | ● | ○ | essential |

Cancella selezione

Does your paper address subitem 11a-i? *

Copy and paste relevant sections from the manuscript (include quotes in quotation marks "like this" to indicate direct quotes from your manuscript), or elaborate on this item by providing additional information not in the ms, or briefly explain why the item is not applicable/relevant for your study

Blinding to the allocation of physicians, dietitians, and psychologists was not possible, given the nature of the intervention. The physician explained to the participants how to download, access, and use the assigned app.

11a-ii) Discuss e.g., whether participants knew which intervention was the “intervention of interest” and which one was the “comparator”

Informed consent procedures (4a-ii) can create biases and certain expectations - discuss e.g., whether participants knew which intervention was the “intervention of interest” and which one was the “comparator”.

| | 1 | 2 | 3 | 4 | 5 | |
|---|---|---|---|---|---|---|
| subitem not at all important | ○ | ○ | ○ | ◉ | ○ | essential |

Cancella selezione

Does your paper address subitem 11a-ii?

Copy and paste relevant sections from the manuscript (include quotes in quotation marks "like this" to indicate direct quotes from your manuscript), or elaborate on this item by providing additional information not in the ms, or briefly explain why the item is not applicable/relevant for your study

In this study, participants were aware that they were being assigned to either the digital therapeutic (DTxO) app or the placebo app. However, they were not explicitly informed which app was the "intervention of interest" and which one was the "comparator."

11b) If relevant, description of the similarity of interventions

(this item is usually not relevant for ehealth trials as it refers to similarity of a placebo or sham intervention to a active medication/intervention)

Does your paper address CONSORT subitem 11b? *

Copy and paste relevant sections from the manuscript (include quotes in quotation marks "like this" to indicate direct quotes from your manuscript), or elaborate on this item by providing additional information not in the ms, or briefly explain why the item is not applicable/relevant for your study

In this trial, the placebo app was designed to closely resemble the digital therapeutic (DTxO) app in terms of its interface and user experience. However, the placebo app did not provide the active features or content aimed at addressing the targeted health outcomes, such as the tailored guidance or behavioral interventions found in the DTxO app. The main difference between the two apps was that the placebo app lacked the therapeutic functionalities, making it a "sham" intervention that served as a control. While both apps had similar user interfaces and engagement prompts to minimize bias, the active DTxO app contained specific tools and content for improving eating behaviors and promoting lifestyle changes, which were absent from the placebo app. Therefore, although the interventions were designed to be similar in their appearance, they differed significantly in their content and therapeutic purpose.

12a) Statistical methods used to compare groups for primary and secondary outcomes

NPT: When applicable, details of whether and how the clustering by care providers or centers was addressed

Does your paper address CONSORT subitem 12a? *

Copy and paste relevant sections from the manuscript (include quotes in quotation marks "like this" to indicate direct quotes from your manuscript), or elaborate on this item by providing additional information not in the ms, or briefly explain why the item is not applicable/relevant for your study

The characteristics of the enrolled participants were described using median and interquartile range (IQR) or frequency and percentage (%), both overall and in each study arm.
Comparisons among arms were calculated with the Wilcoxon rank-sum test for continuous variables, chi-square test, or Fisher's exact test for categorical ones, as appropriate.
Absolute and percent changes in weight, BMI, and waist circumference were calculated overall and within each study arm and tested by the Wilcoxon signed-rank test.
Pearson's correlation coefficients were calculated to assess the presence of a linear relationship between the 6-month absolute or percent change from baseline in weight with overall adherence.

Univariable and multivariable generalized linear models (GLM) were fitted to determine factors associated with the 6-month absolute (primary endpoint) or percent change (secondary endpoint) in body weight; slopes with the corresponding standard errors or 95% confidence intervals (95%CI) have been estimated. The multivariable models included the study arm, overall adherence, and covariates with a p-value ≤0.10 at univariable regression models, avoiding multicollinearity among the included covariates.

Two-sided p-values <0.05 were considered significant. Statistical analyses were carried out using the SAS software (SAS Institute, Cary, NC, release 9.4).

12a-i) Imputation techniques to deal with attrition / missing values

Imputation techniques to deal with attrition / missing values: Not all participants will use the intervention/comparator as intended and attrition is typically high in ehealth trials. Specify how participants who did not use the application or dropped out from the trial were treated in the statistical analysis (a complete case analysis is strongly discouraged, and simple imputation techniques such as LOCF may also be problematic [4]).

| | 1 | 2 | 3 | 4 | 5 | |
|---|---|---|---|---|---|---|
| subitem not at all important | ○ | ○ | ○ | ◉ | ○ | essential |

Cancella selezione

Does your paper address subitem 12a-i? *

Copy and paste relevant sections from the manuscript (include quotes in quotation marks "like this" to indicate direct quotes from your manuscript), or elaborate on this item by providing additional information not in the ms, or briefly explain why the item is not applicable/relevant for your study

In our study, we applied appropriate imputation techniques to handle missing values due to participant attrition. Specifically, we used the Last Observation Carried Forward (LOCF) method, where the last available data point for a participant was used to replace any missing follow-up values. This approach assumes that the participant's status did not significantly change between the last observed measurement and the missing data point.

12b) Methods for additional analyses, such as subgroup analyses and adjusted analyses

Does your paper address CONSORT subitem 12b? *

Copy and paste relevant sections from the manuscript (include quotes in quotation marks "like this" to indicate direct quotes from your manuscript), or elaborate on this item by providing additional information not in the ms, or briefly explain why the item is not applicable/relevant for your study

In the analyses, overall adherence was stratified into two classes as it was found to have an important effect on weight change: a value at or above the 75th percentile (medium-elevated) for overall adherence; and values lower than the 75th percentile (low-scarce). The 75th percentile was calculated on non-transformed overall adherence value, obtained from the overall enrolled participants.

X26) REB/IRB Approval and Ethical Considerations [recommended as subheading under "Methods"] (not a CONSORT item)

X26-i) Comment on ethics committee approval

| | 1 | 2 | 3 | 4 | 5 | |
|---|---|---|---|---|---|---|
| subitem not at all important | ○ | ○ | ○ | ◉ | ○ | essential |

Cancella selezione

Does your paper address subitem X26-i?

Copy and paste relevant sections from the manuscript (include quotes in quotation marks "like this" to indicate direct quotes from your manuscript), or elaborate on this item by providing additional information not in the ms, or briefly explain why the item is not applicable/relevant for your study

Ethical approval for the study was granted by the Ethical Committees of both centers (Center 1: approval number 2022_04-12_03; Center 2: approval number 7392). These approvals affirm that the study met the required ethical standards for research involving human subjects.

x26-ii) Outline informed consent procedures

Outline informed consent procedures e.g., if consent was obtained offline or online (how? Checkbox, etc.?), and what information was provided (see 4a-ii). See [6] for some items to be included in informed consent documents.

| | 1 | 2 | 3 | 4 | 5 | |
|---|---|---|---|---|---|---|
| subitem not at all important | ○ | ○ | ○ | ◉ | ○ | essential |

Cancella selezione

Does your paper address subitem X26-ii?

Copy and paste relevant sections from the manuscript (include quotes in quotation marks "like this" to indicate direct quotes from your manuscript), or elaborate on this item by providing additional information not in the ms, or briefly explain why the item is not applicable/relevant for your study

Informed consent descriptions: Informed consent document outlined the clinical trial's purpose, key details, and the rights and responsibilities of participants, ensuring they could make an informed, voluntary decision. Participants were encouraged to ask questions and consult with their doctor or trusted individuals. They were given time to review and discuss the document before deciding, and if they chose not to participate, they still received the best possible care. Informed consent was obtained in writing from each participant prior to their involvement.

X26-iii) Safety and security procedures

Safety and security procedures, incl. privacy considerations, and any steps taken to reduce the likelihood or detection of harm (e.g., education and training, availability of a hotline)

| | 1 | 2 | 3 | 4 | 5 | |
|---|---|---|---|---|---|---|
| subitem not at all important | ○ | ○ | ○ | ◉ | ○ | essential |

Cancella selezione

Does your paper address subitem X26-iii?

Copy and paste relevant sections from the manuscript (include quotes in quotation marks "like this" to indicate direct quotes from your manuscript), or elaborate on this item by providing additional information not in the ms, or briefly explain why the item is not applicable/relevant for your study

At follow up visit safety assessment was performed.

RESULTS

13a) For each group, the numbers of participants who were randomly assigned, received intended treatment, and were analysed for the primary outcome

NPT: The number of care providers or centers performing the intervention in each group and the number of patients treated by each care provider in each center

Does your paper address CONSORT subitem 13a? *

Copy and paste relevant sections from the manuscript (include quotes in quotation marks "like this" to indicate direct quotes from your manuscript), or elaborate on this item by providing additional information not in the ms, or briefly explain why the item is not applicable/relevant for your study

The screening was performed on 280 participants (Figure 2); 33 patients were not eligible, and one declined participation. In total, 246 participants were randomly assigned to DTxO or the Placebo App group (N =123, each),

13b) For each group, losses and exclusions after randomisation, together with reasons

Does your paper address CONSORT subitem 13b? (NOTE: Preferably, this is shown in a CONSORT flow diagram) *

Copy and paste relevant sections from the manuscript (include quotes in quotation marks "like this" to indicate direct quotes from your manuscript), or elaborate on this item by providing additional information not in the ms, or briefly explain why the item is not applicable/relevant for your study

Of 246 participants, 207 completed the assessment after 6-month from the end of the intervention [105 in the DTxO group and 102 in the Placebo App group]; 39 participants [18 in the DTxO group and 21 in the Placebo App group] discontinued: 19 withdrew informed consent, 1 started therapy with GLP-1 analogue (Liraglutide) and 19 were lost to follow-up.

13b-i) Attrition diagram

Strongly recommended: An attrition diagram (e.g., proportion of participants still logging in or using the intervention/comparator in each group plotted over time, similar to a survival curve) or other figures or tables demonstrating usage/dose/engagement.

| | 1 | 2 | 3 | 4 | 5 | |
|---|---|---|---|---|---|---|
| subitem not at all important | ○ | ○ | ○ | ○ | ◉ | essential |

Cancella selezione

Does your paper address subitem 13b-i?

Copy and paste relevant sections from the manuscript or cite the figure number if applicable (include quotes in quotation marks "like this" to indicate direct quotes from your manuscript), or elaborate on this item by providing additional information not in the ms, or briefly explain why the item is not applicable/relevant for your study

Figure 2 shows Patient flow diagram

14a) Dates defining the periods of recruitment and follow-up

Does your paper address CONSORT subitem 14a? *

Copy and paste relevant sections from the manuscript (include quotes in quotation marks "like this" to indicate direct quotes from your manuscript), or elaborate on this item by providing additional information not in the ms, or briefly explain why the item is not applicable/relevant for your study

The total duration of the study to evaluate primary outcome was 10 months, consisting of a 4-month recruitment phase followed by a 6-month follow-up period after the initial baseline visit.
Enrolment took place during a visit with an endocrinologist or nutrition specialist, with informed consent obtained before any screening procedures were conducted.

14a-i) Indicate if critical “secular events” fell into the study period

Indicate if critical “secular events” fell into the study period, e.g., significant changes in Internet resources available or “changes in computer hardware or Internet delivery resources”

| | 1 | 2 | 3 | 4 | 5 | |
|---|---|---|---|---|---|---|
| subitem not at all important | ○ | ○ | ◉ | ○ | ○ | essential |

Cancella selezione

Does your paper address subitem 14a-i?

Copy and paste relevant sections from the manuscript (include quotes in quotation marks "like this" to indicate direct quotes from your manuscript), or elaborate on this item by providing additional information not in the ms, or briefly explain why the item is not applicable/relevant for your study

No critical secular events occurred during the study period that could have influenced the outcomes. The trial was conducted in a stable context without major external events (e.g., public health crises, policy changes, or natural disasters) that might have affected participant behavior or the implementation of the intervention.

14b) Why the trial ended or was stopped (early)

Does your paper address CONSORT subitem 14b? *

Copy and paste relevant sections from the manuscript (include quotes in quotation marks "like this" to indicate direct quotes from your manuscript), or elaborate on this item by providing additional information not in the ms, or briefly explain why the item is not applicable/relevant for your study

The trial was not ended or stopped early. It was conducted and completed as originally planned according to the study protocol, with all follow-up and data collection phases carried out as scheduled.

15) A table showing baseline demographic and clinical characteristics for each group

NPT: When applicable, a description of care providers (case volume, qualification, expertise, etc.) and centers (volume) in each group

Does your paper address CONSORT subitem 15? *

Copy and paste relevant sections from the manuscript (include quotes in quotation marks "like this" to indicate direct quotes from your manuscript), or elaborate on this item by providing additional information not in the ms, or briefly explain why the item is not applicable/relevant for your study

yes, Table 1 showed Baseline characteristics

15-i) Report demographics associated with digital divide issues

In ehealth trials it is particularly important to report demographics associated with digital divide issues, such as age, education, gender, social-economic status, computer/Internet/ehealth literacy of the participants, if known.

| | 1 | 2 | 3 | 4 | 5 | |
|---|---|---|---|---|---|---|
| subitem not at all important | ○ | ○ | ○ | ◉ | ○ | essential |

Cancella selezione

Does your paper address subitem 15-i? *

Copy and paste relevant sections from the manuscript (include quotes in quotation marks "like this" to indicate direct quotes from your manuscript), or elaborate on this item by providing additional information not in the ms, or briefly explain why the item is not applicable/relevant for your study

Women were about 2/3 of the study sample. The majority (N=243, 99%) of participants were white and highly educated, reflecting the level of metropolitan areas in which enrolment occurred. Specifically, the educational attainment distribution within the total sample was as follows: 0.4% (N=1) had completed primary school, 70.7% (N=174) had completed high school, 25.2% (N=62) held a university degree, and 3.6% (N=9) had obtained a master's degree or PhD, with no differences between the two groups. Regarding marital status, 61.4% (N=151) of the participants were married, 31.7% (N=78) were single, 6.5% (N=16) were separated or divorced, and 0.4% (N=1) were widowed, with no significant differences between the two groups.

16) For each group, number of participants (denominator) included in each analysis and whether the analysis was by original assigned groups

16-i) Report multiple “denominators” and provide definitions

Report multiple “denominators” and provide definitions: Report N’s (and effect sizes) “across a range of study participation [and use] thresholds” [1], e.g., N exposed, N consented, N used more than x times, N used more than y weeks, N participants “used” the intervention/comparator at specific pre-defined time points of interest (in absolute and relative numbers per group). Always clearly define “use” of the intervention.

| | 1 | 2 | 3 | 4 | 5 | |
|---|---|---|---|---|---|---|
| subitem not at all important | ○ | ○ | ○ | ○ | ◉ | essential |

Cancella selezione

Does your paper address subitem 16-i? *

Copy and paste relevant sections from the manuscript (include quotes in quotation marks "like this" to indicate direct quotes from your manuscript), or elaborate on this item by providing additional information not in the ms, or briefly explain why the item is not applicable/relevant for your study

Multiple denominators were reported and defined to capture the different levels of participant engagement with the intervention.

16-ii) Primary analysis should be intent-to-treat

Primary analysis should be intent-to-treat, secondary analyses could include comparing only "users", with the appropriate caveats that this is no longer a randomized sample (see 18-i).

| | 1 | 2 | 3 | 4 | 5 | |
|---|---|---|---|---|---|---|
| subitem not at all important | ○ | ○ | ○ | ◉ | ○ | essential |

Cancella selezione

Does your paper address subitem 16-ii?

Copy and paste relevant sections from the manuscript (include quotes in quotation marks "like this" to indicate direct quotes from your manuscript), or elaborate on this item by providing additional information not in the ms, or briefly explain why the item is not applicable/relevant for your study

The primary analysis was conducted using an intention-to-treat (ITT) approach, including all participants as originally allocated after randomization, regardless of their level of adherence or app usage. As a secondary analysis, outcomes were also compared between participants with overall adherence levels at or above the 75th percentile (medium-to-high adherence) and those below the 75th percentile (low-to-limited adherence). Appropriate caveats regarding the loss of randomization and potential bias in this analysis are discussed in the manuscript.

17a) For each primary and secondary outcome, results for each group, and the estimated effect size and its precision (such as 95% confidence interval)

Does your paper address CONSORT subitem 17a? *

Copy and paste relevant sections from the manuscript (include quotes in quotation marks "like this" to indicate direct quotes from your manuscript), or elaborate on this item by providing additional information not in the ms, or briefly explain why the item is not applicable/relevant for your study

For each primary and secondary outcome, results for both the intervention and control groups are reported, including the estimated effect sizes and their precision, expressed as 95% confidence intervals. These data are presented in the Results section and detailed in the corresponding tables to ensure clarity, accuracy, and transparency of the findings.

17a-i) Presentation of process outcomes such as metrics of use and intensity of use

In addition to primary/secondary (clinical) outcomes, the presentation of process outcomes such as metrics of use and intensity of use (dose, exposure) and their operational definitions is critical. This does not only refer to metrics of attrition (13-b) (often a binary variable), but also to more continuous exposure metrics such as “average session length”. These must be accompanied by a technical description how a metric like a “session” is defined (e.g., timeout after idle time) [1] (report under item 6a).

| | 1 | 2 | 3 | 4 | 5 | |
|---|---|---|---|---|---|---|
| subitem not at all important | ○ | ○ | ○ | ○ | ○ | essential |

Does your paper address subitem 17a-i?

Copy and paste relevant sections from the manuscript (include quotes in quotation marks "like this" to indicate direct quotes from your manuscript), or elaborate on this item by providing additional information not in the ms, or briefly explain why the item is not applicable/relevant for your study

Process outcomes such as metrics of use and intensity of use are presented in the study. These include measures like frequency of app usage, session duration, and adherence levels.

17b) For binary outcomes, presentation of both absolute and relative effect sizes is recommended

Does your paper address CONSORT subitem 17b? *

Copy and paste relevant sections from the manuscript (include quotes in quotation marks "like this" to indicate direct quotes from your manuscript), or elaborate on this item by providing additional information not in the ms, or briefly explain why the item is not applicable/relevant for your study

For binary outcomes, we reported both absolute and relative effect sizes, as recommended.

18) Results of any other analyses performed, including subgroup analyses and adjusted analyses, distinguishing pre-specified from exploratory

Does your paper address CONSORT subitem 18? *

Copy and paste relevant sections from the manuscript (include quotes in quotation marks "like this" to indicate direct quotes from your manuscript), or elaborate on this item by providing additional information not in the ms, or briefly explain why the item is not applicable/relevant for your study

Additional analyses were performed, including both subgroup analyses and adjusted analyses.

18-i) Subgroup analysis of comparing only users

A subgroup analysis of comparing only users is not uncommon in ehealth trials, but if done, it must be stressed that this is a self-selected sample and no longer an unbiased sample from a randomized trial (see 16-iii).

| | 1 | 2 | 3 | 4 | 5 | |
|---|---|---|---|---|---|---|
| subitem not at all important | ○ | ○ | ○ | ◉ | ○ | essential |

Cancella selezione

Does your paper address subitem 18-i?

Copy and paste relevant sections from the manuscript (include quotes in quotation marks "like this" to indicate direct quotes from your manuscript), or elaborate on this item by providing additional information not in the ms, or briefly explain why the item is not applicable/relevant for your study

A subgroup analysis comparing only users is not uncommon in eHealth trials, and we have conducted such an analysis as part of our secondary outcomes.

19) All important harms or unintended effects in each group
(for specific guidance see CONSORT for harms)

Does your paper address CONSORT subitem 19? *

Copy and paste relevant sections from the manuscript (include quotes in quotation marks "like this" to indicate direct quotes from your manuscript), or elaborate on this item by providing additional information not in the ms, or briefly explain why the item is not applicable/relevant for your study

During the course of the study, we closely monitored for any potential harms or unintended effects in both the intervention and control groups. However, no significant adverse events or unintended effects were reported by participants in either group.

19-i) Include privacy breaches, technical problems

Include privacy breaches, technical problems. This does not only include physical “harm” to participants, but also incidents such as perceived or real privacy breaches [1], technical problems, and other unexpected/unintended incidents. “Unintended effects” also includes unintended positive effects [2].

| | 1 | 2 | 3 | 4 | 5 | |
|---|---|---|---|---|---|---|
| subitem not at all important | ○ | ○ | ○ | ○ | ● | essential |

Cancella selezione

Does your paper address subitem 19-i?

Copy and paste relevant sections from the manuscript (include quotes in quotation marks "like this" to indicate direct quotes from your manuscript), or elaborate on this item by providing additional information not in the ms, or briefly explain why the item is not applicable/relevant for your study

During the study, we closely monitored privacy and technical issues to ensure the security and integrity of participants' data. No privacy breaches were reported, and all participant data were securely stored in accordance with relevant data protection regulations.

19-ii) Include qualitative feedback from participants or observations from staff/researchers

Include qualitative feedback from participants or observations from staff/researchers, if available, on strengths and shortcomings of the application, especially if they point to unintended/unexpected effects or uses. This includes (if available) reasons for why people did or did not use the application as intended by the developers.

| | 1 | 2 | 3 | 4 | 5 | |
|---|---|---|---|---|---|---|
| subitem not at all important | ○ | ○ | ○ | ◉ | ○ | essential |

Cancella selezione

Does your paper address subitem 19-ii?

Copy and paste relevant sections from the manuscript (include quotes in quotation marks "like this" to indicate direct quotes from your manuscript), or elaborate on this item by providing additional information not in the ms, or briefly explain why the item is not applicable/relevant for your study

Qualitative feedback from participants was not formally collected during the study, as no structured interviews or surveys were conducted to gather such insights. However, observations from the research staff indicated that participants generally found the digital therapeutic app user-friendly and engaging.

DISCUSSION

22) Interpretation consistent with results, balancing benefits and harms, and considering other relevant evidence

NPT: In addition, take into account the choice of the comparator, lack of or partial blinding, and unequal expertise of care providers or centers in each group

22-i) Restate study questions and summarize the answers suggested by the data, starting with primary outcomes and process outcomes (use)

Restate study questions and summarize the answers suggested by the data, starting with primary outcomes and process outcomes (use).

| | 1 | 2 | 3 | 4 | 5 | |
|---|---|---|---|---|---|---|
| subitem not at all important | ○ | ○ | ○ | ○ | ◉ | essential |

Cancella selezione

Does your paper address subitem 22-i? *

Copy and paste relevant sections from the manuscript (include quotes in quotation marks "like this" to indicate direct quotes from your manuscript), or elaborate on this item by providing additional information not in the ms, or briefly explain why the item is not applicable/relevant for your study

The interpretation of our results is consistent with the data presented, balancing the observed benefits and any potential harms.

22-ii) Highlight unanswered new questions, suggest future research

Highlight unanswered new questions, suggest future research.

| | 1 | 2 | 3 | 4 | 5 | |
|---|---|---|---|---|---|---|
| subitem not at all important | ○ | ○ | ○ | ○ | ○ | essential |

Does your paper address subitem 22-ii?

Copy and paste relevant sections from the manuscript (include quotes in quotation marks "like this" to indicate direct quotes from your manuscript), or elaborate on this item by providing additional information not in the ms, or briefly explain why the item is not applicable/relevant for your study

Further research is needed to identify the most suitable candidates for each treatment modality (digital, in-person, or hybrid), considering clinical characteristics, demographics, and patient preferences.

20) Trial limitations, addressing sources of potential bias, imprecision, and, if relevant, multiplicity of analyses

20-i) Typical limitations in ehealth trials

Typical limitations in ehealth trials: Participants in ehealth trials are rarely blinded. Ehealth trials often look at a multiplicity of outcomes, increasing risk for a Type I error. Discuss biases due to non-use of the intervention/usability issues, biases through informed consent procedures, unexpected events.

| | 1 | 2 | 3 | 4 | 5 | |
|---|---|---|---|---|---|---|
| subitem not at all important | ○ | ○ | ○ | ◉ | ○ | essential |

Cancella selezione

Does your paper address subitem 20-i? *

Copy and paste relevant sections from the manuscript (include quotes in quotation marks "like this" to indicate direct quotes from your manuscript), or elaborate on this item by providing additional information not in the ms, or briefly explain why the item is not applicable/relevant for your study

This study has several limitations. First, blinding of the researchers was not possible due to the nature of the intervention.

21) Generalisability (external validity, applicability) of the trial findings

NPT: External validity of the trial findings according to the intervention, comparators, patients, and care providers or centers involved in the trial

21-i) Generalizability to other populations

Generalizability to other populations: In particular, discuss generalizability to a general Internet population, outside of a RCT setting, and general patient population, including applicability of the study results for other organizations

| | 1 | 2 | 3 | 4 | 5 | |
|---|---|---|---|---|---|---|
| subitem not at all important | ○ | ○ | ○ | ○ | ◉ | essential |

Cancella selezione

Does your paper address subitem 21-i?

Copy and paste relevant sections from the manuscript (include quotes in quotation marks "like this" to indicate direct quotes from your manuscript), or elaborate on this item by providing additional information not in the ms, or briefly explain why the item is not applicable/relevant for your study

The generalizability of the study may be limited by the recruitment strategy, which targeted individuals with social media accounts and proficient knowledge of the Italian language, as the content was not available in other languages.

21-ii) Discuss if there were elements in the RCT that would be different in a routine application setting

Discuss if there were elements in the RCT that would be different in a routine application setting (e.g., prompts/reminders, more human involvement, training sessions or other co-interventions) and what impact the omission of these elements could have on use, adoption, or outcomes if the intervention is applied outside of a RCT setting.

| | 1 | 2 | 3 | 4 | 5 | |
|---|---|---|---|---|---|---|
| subitem not at all important | ○ | ○ | ○ | ◉ | ○ | essential |

Cancella selezione

Does your paper address subitem 21-ii?

Copy and paste relevant sections from the manuscript (include quotes in quotation marks "like this" to indicate direct quotes from your manuscript), or elaborate on this item by providing additional information not in the ms, or briefly explain why the item is not applicable/relevant for your study

In a routine application setting, several elements of the randomized controlled trial (RCT) would likely differ due to the controlled nature of the trial and the real-world conditions.While the trial setting was designed to maximize control and ensure reliable data, a routine application would likely introduce more variability and potentially fewer resources for monitoring and support. Understanding how these differences influence outcomes is critical for determining how well the intervention can translate to real-world use.

OTHER INFORMATION

23) Registration number and name of trial registry

Does your paper address CONSORT subitem 23? *

Copy and paste relevant sections from the manuscript (include quotes in quotation marks "like this" to indicate direct quotes from your manuscript), or elaborate on this item by providing additional information not in the ms, or briefly explain why the item is not applicable/relevant for your study

clinicaltrials.gov, identifier, NCT05394779. DEMETRA STUDY

24) Where the full trial protocol can be accessed, if available

Does your paper address CONSORT subitem 24? *

Cite a Multimedia Appendix, other reference, or copy and paste relevant sections from the manuscript (include quotes in quotation marks "like this" to indicate direct quotes from your manuscript), or elaborate on this item by providing additional information not in the ms, or briefly explain why the item is not applicable/relevant for your study

Castelnuovo, G., Capodaglio P, De Amicis R, Gilardini L, Mambrini SP. et al. Study protocol of a clinical randomized controlled trial on the efficacy of an innovative digital therapy to promote weight loss in patients with obesity by increasing their adherence to treatment: the DEMETRA study. Front. Digit. Heal.2023; 5, 1–7. DOI: 10.3389/fdgth.2023.1159744

25) Sources of funding and other support (such as supply of drugs), role of funders

Does your paper address CONSORT subitem 25? *

Copy and paste relevant sections from the manuscript (include quotes in quotation marks "like this" to indicate direct quotes from your manuscript), or elaborate on this item by providing additional information not in the ms, or briefly explain why the item is not applicable/relevant for your study

This research was funded by Theras Lifetech Srl Unipersonale. The funder had no role in any aspect of the development, conduct, analysis, or reporting of the study.

X27) Conflicts of Interest (not a CONSORT item)

X27-i) State the relation of the study team towards the system being evaluated

In addition to the usual declaration of interests (financial or otherwise), also state the relation of the study team towards the system being evaluated, i.e., state if the authors/evaluators are distinct from or identical with the developers/sponsors of the intervention.

| | 1 | 2 | 3 | 4 | 5 | |
|---|---|---|---|---|---|---|
| subitem not at all important | ○ | ○ | ○ | ● | ○ | essential |

Cancella selezione

Does your paper address subitem X27-i?

Copy and paste relevant sections from the manuscript (include quotes in quotation marks "like this" to indicate direct quotes from your manuscript), or elaborate on this item by providing additional information not in the ms, or briefly explain why the item is not applicable/relevant for your study

The study team is directly involved in the development, evaluation, and testing of the system being assessed. Specifically, the digital therapeutic (DTxO) and placebo app were both developed by the investigative team. As part of the study, the team not only designed and implemented the intervention but also conducted the clinical trial to evaluate its effectiveness.

About the CONSORT EHEALTH checklist

As a result of using this checklist, did you make changes in your manuscript? *

- ○ yes, major changes
- ◉ yes, minor changes
- ○ no

What were the most important changes you made as a result of using this checklist?

As a result of using this checklist, we made several important changes to enhance the clarity and rigor of our manuscript. First, we ensured that we explicitly described the theoretical foundations guiding our interventions, particularly the use of mindfulness in the psycho-behavioral component. We also revised the description of the intervention process to make clear the involvement of healthcare professionals and the type of support provided to participants. These changes aimed to improve the transparency and comprehensiveness of our study, ensuring that all necessary information is available to reviewers and readers.

How much time did you spend on going through the checklist INCLUDING making changes in your manuscript *

I spent several hours going through the checklist, reviewing each item in detail, and making the necessary changes to the manuscript to ensure compliance with the required guidelines.

As a result of using this checklist, do you think your manuscript has improved? *

- (selected) yes
- no
- Altro:

Would you like to become involved in the CONSORT EHEALTH group?

This would involve for example becoming involved in participating in a workshop and writing an "Explanation and Elaboration" document

- (selected) yes
- no
- Altro:

Cancella selezione

Any other comments or questions on CONSORT EHEALTH

La tua risposta

STOP - Save this form as PDF before you click submit

To generate a record that you filled in this form, we recommend to generate a PDF of this page (on a Mac, simply select "print" and then select "print as PDF") before you submit it.

When you submit your (revised) paper to JMIR, please upload the PDF as supplementary file.

Don't worry if some text in the textboxes is cut off, as we still have the complete information in our database. Thank you!

Final step: Click submit !

Click submit so we have your answers in our database!

Invia Cancella modulo

Non inviare mai le password tramite Moduli Google.

Questi contenuti non sono creati né avallati da Google. - Termini di servizio - Norme sulla privacy

Questo modulo sembra sospetto? Segnala

Google Moduli
